# Measuring psychotherapeutic processes in the context of psychedelic experiences: Validation of the General Change Mechanisms Questionnaire (GCMQ)

**DOI:** 10.1177/02698811241249698

**Published:** 2024-05-14

**Authors:** Max Wolff, Ricarda Evens, Lea J Mertens, Christopher Schmidt, Jessica Beck, Hans Rutrecht, Aaron D Cherniak, Gerhard Gründer, Henrik Jungaberle

**Affiliations:** 1 Department of Psychiatry and Neurosciences, Charité—Universitätsmedizin Berlin, Campus Charité Mitte, Berlin, Germany; 2 MIND Foundation, Berlin, Germany; 3 Department of Psychology, Humboldt-Universität zu Berlin, Berlin, Germany; 4 Department of Molecular Neuroimaging, Central Institute of Mental Health, Medical Faculty Mannheim, University of Heidelberg, Mannheim, Germany; 5 Faculty of Psychology, Technische Universität Dresden, Dresden, Germany; 6 Department of Psychology, Stockholm University, Stockholm, Sweden; 7 Department of Psychology, Reichman University, Herzliya, Israel

**Keywords:** Psychedelics, general change mechanisms, common factors, assessment, use motives

## Abstract

**Background:**

Therapeutic and salutogenic effects of psychedelic drugs have been attributed to psychotherapeutic or psychotherapy-like processes that can unfold during the acute psychedelic experience and beyond. Currently, there are no psychometric instruments available to comprehensively assess psychotherapeutic processes (as conceptualized by empirical psychotherapy research) in the context of psychedelic experiences.

**Aims:**

We report the initial validation of the General Change Mechanisms Questionnaire (GCMQ), a self-report instrument designed to measure five empirically established general change mechanisms (GCMs) of psychotherapy—(1) resource activation, (2) therapeutic relationship, (3) problem actuation, (4) clarification, and (5) mastery—in the context of psychedelic experiences.

**Methods:**

An online survey in a sample of 1153 English-speaking and 714 German-speaking psychedelic users was conducted to evaluate simultaneously developed English- and German-language versions of the GCMQ.

**Results:**

The theory-based factor structure was confirmed. The five GCMQ scales showed good internal consistency. Evidence for convergent validity with external measures was obtained. Significant associations with different settings and with therapeutic, hedonic, and escapist use motives confirmed the hypothesized context dependence of GCM-related psychedelic experiences. Indicating potential therapeutic effects, the association between cumulative stressful life events and well-being was significantly moderated by resource activation, clarification, and mastery. Factor mixture modeling revealed five distinct profiles of GCM-related psychedelic experiences.

**Conclusion:**

Initial testing indicates that the GCMQ is a valid and reliable instrument that can be used in future clinical and nonclinical psychedelic research. The five identified profiles of GCM-related experiences may be relevant to clinical uses of psychedelics and psychedelic harm reduction.

## Introduction

Classical psychedelics, such as psilocybin or lysergic acid diethylamide (LSD), have garnered attention for their potential to induce lasting psychological changes beyond their acute effects. Clinical trials exploring *psychedelic therapy* usually involve a limited number of psychedelic dosing sessions, typically one to three, embedded within a framework of several preparatory and integration-focused psychotherapy sessions (Garcia-Romeu and Richards, 2018). Although sometimes referred to as “psychological support,” these treatment frameworks correspond to standard definitions of psychotherapy (Gründer et al., 2023). Historically and in recent years, clinical trials have shown promising results for various mental health conditions, including depression, anxiety disorders, substance use disorders, and distress associated with chronic or terminal illness (Mitchell and Anderson, 2023; Nichols and Walter, 2021).

Furthermore, the therapeutic and salutogenic effects of psychedelics can extend beyond their clinical use: Experimental studies with healthy volunteers reveal that, under favorable circumstances, psychedelics can promote long-lasting positive changes in various domains of psychosocial functioning, including trait mindfulness, well-being, and life satisfaction (e.g., Griffiths et al., 2018; Madsen et al., 2020; Smigielski et al., 2019; Timmermann et al., 2024). Besides clinical and experimental studies, observational studies have documented the effects of different forms of *naturalistic psychedelic use*, including recreational use for hedonic motives, ceremonial use for spiritual or religious motives, and therapeutic or self-medication use to promote mental or physical health (Roberts et al., 2020). The study of naturalistic use is relevant to clinical applications of psychedelics since it provides insights into psychedelic-occasioned psychological change and its contextual conditions. While psychedelic use in uncontrolled settings can cause harm (Carbonaro et al., 2016; Evans et al., 2023), epidemiological studies reveal that, quite unlike other drug use, psychedelic use is not positively but rather negatively associated with mental health problems (Johnson et al., 2019). Consistent with this, survey studies among psychedelic users have found evidence for positive psychological effects, including enhanced mental well-being, psychological flexibility, and reduced psychopathology (Aday et al., 2020; Davis et al., 2020; Haijen et al., 2018; Nayak et al., 2023; Wolff et al., 2022).

### A contextual-experiential model of psychedelic drug effects

**Figure 1.**
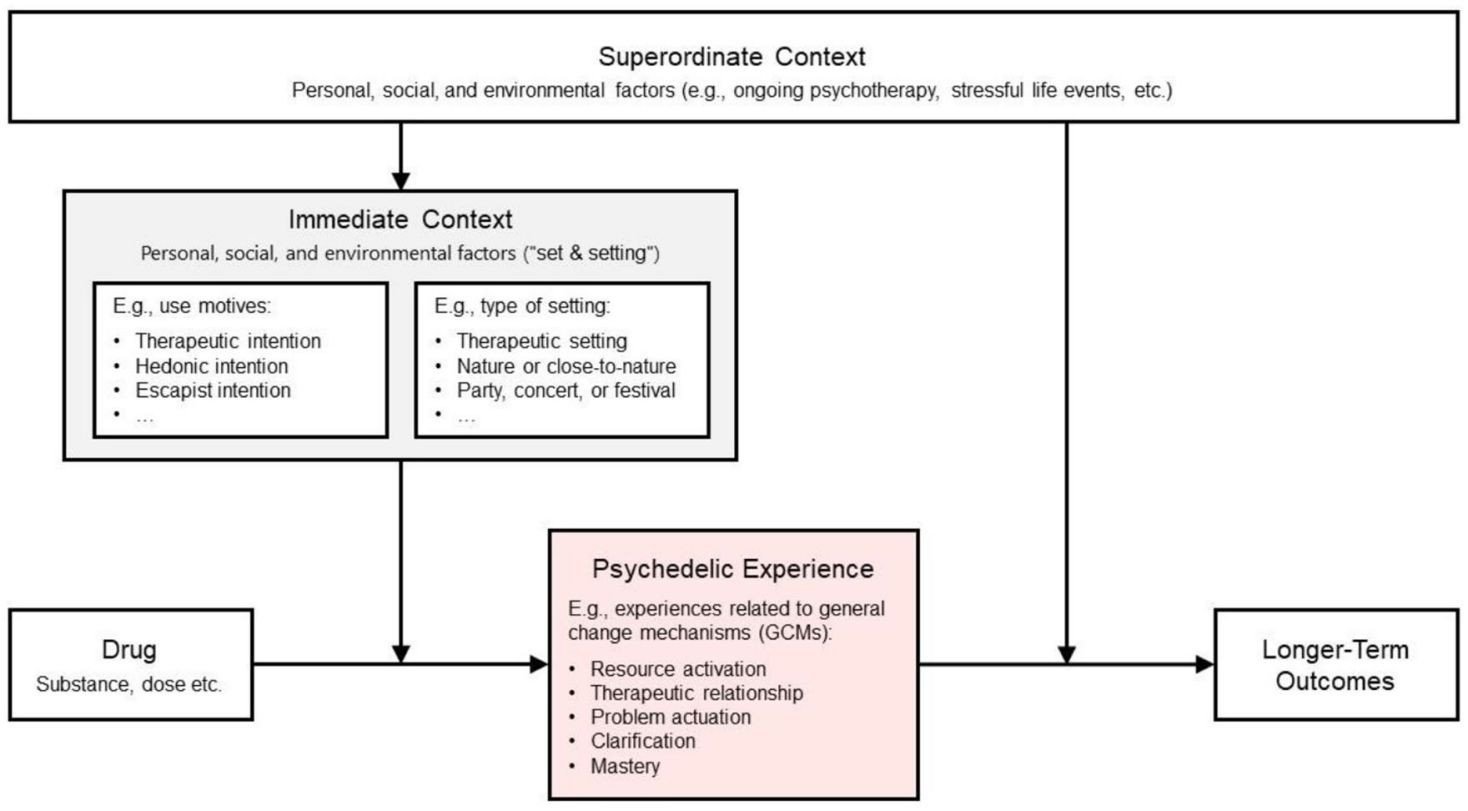
Contextual-experiential model illustrating the assumed context dependence of psychedelic drugs’ acute and longer-term effects (see also Carhart-Harris and Nutt, 2017). The acute psychedelic experience is assumed to mediate longer-term outcomes. Immediate and superordinate context factors exert moderating effects on the acute drug effect and the learning processes (commonly referred to as “integration”) that mediate between the psychedelic experience and longer-term outcomes.

It is widely acknowledged that longer-term psychological changes occasioned by psychedelic drugs depend to a substantial extent on the acute subjective drug effect or *psychedelic experience* (Aday et al., 2021; Yaden and Griffiths, 2021). The psychedelic experience, in turn, strongly depends on context factors, that is, the psychological, social, and environmental situation in which the experience occurs (“set and setting”; Carhart-Harris et al., 2018; Hartogsohn, 2017). Figure 1 illustrates the putative roles of the psychedelic experience and context factors in shaping psychedelic-occasioned psychological change.

Agreeing with others who have pointed out that psychedelic therapy shares substantial “common factors” with other psychotherapies (Aday et al., 2023; Greenway et al., 2020; Nayak and Johnson, 2020), we assume that therapeutically effective psychedelic experiences are similar to those experiences which are purposefully induced in effective psychotherapy (Gründer et al., 2023; Jungaberle et al., 2008; Wolff et al., 2020, 2022). Our contextual-experiential model (Figure 1) suggests that such psychotherapy-like psychedelic experiences are most likely to occur in the context of formal psychotherapy and other social contexts that engender interpersonal trust, emotional openness, introspective attention, and self-inquiry. Two related aspects of immediate context that the present work focuses on are different *types of settings* (including therapeutic settings) and *use motives* (including therapeutic use motives). Survey studies investigating naturalistic psychedelic use have shown that both setting and use motives are associated with therapeutic qualities of the psychedelic experience (e.g., Haijen et al., 2018; Wolff et al., 2022). These context factors thus represent key targets for therapeutic interventions. In psychedelic therapy, much care is taken in controlling the setting for psychedelic dosing sessions, including the setup of the room, the continuous presence of therapists, the selection of music, and the avoidance of disturbances (Garcia-Romeu and Richards, 2018; Johnson et al., 2008). Furthermore, therapists use preparatory sessions to shape therapeutic use motives by discussing patients’ intentions for dosing sessions (Thal et al., 2022b). However, whereas active fostering of approach-motivated therapy goals is among the most studied interventions in psychotherapy research (DeFife and Hilsenroth, 2011; Wollburg and Braukhaus, 2010), the effects of intentions in psychedelic therapy have not yet been systematically investigated.

Our assumption that psychedelic-occasioned therapeutic experiences are similar to those experiences induced in effective psychotherapy is consistent with the view that psychedelics are “psychotherapeutic drugs” (Müller et al., 2020) that can act as “catalysts” of psychotherapeutic processes (e.g., Sandison, 1954). However, reflecting a historically rooted estrangement between psychedelic and psychotherapy research, the psychological constructs and psychometric instruments that are used to characterize experiences in current psychedelic research are not straightforwardly related to psychotherapeutic change mechanisms as conceptualized by empirical psychotherapy research (Wolff et al., under review). Scientific progress and the development of research-informed practices in psychedelic therapy are thus hindered by a lack of transdisciplinary exchange between psychedelic and psychotherapy research. The present work aims to address this gap by introducing a new psychometric instrument to assess psychotherapeutic processes in the context of psychedelic experiences: the General Change Mechanisms Questionnaire (GCMQ).

### General change mechanisms in psychotherapy and psychedelic-occasioned psychological change

A central subject of psychotherapy research is the question of how psychotherapy works. The concept of *general change mechanisms* (GCMs) posits that the efficacy of all effective psychotherapies, irrespective of the specific therapeutic approach, can be attributed to shared underlying principles of change or “common factors” that can be harnessed by superficially distinct methods (Cuijpers et al., 2019; Frank, 1961; Garfield, 1982; Goldfried, 1980; Grawe, 1997; Orlinsky and Howard, 1987; Rosenzweig, 1936; Wampold and Imel, 2015). An influential conceptualization of this idea are the five GCMs that were identified by Grawe (1997, 2004) based on extensive empirical data from controlled therapy trials and naturalistic studies of the process–outcome relationship in psychotherapy: (1) *resource activation*, (2) *therapeutic relationship*, (3) *problem actuation*, (4) *clarification*, and (5) *mastery*. Accordingly, the efficacy of any psychotherapy—and by implication also psychedelic therapy—is mediated by a synergistic interaction of these GCMs. On a theoretical and empirical basis, it has been argued that Grawe’s GCMs are essential to the therapeutic and salutogenic effects of psychedelic experiences (Jungaberle et al., 2008; Passie and Dürst, 2009; Schlichting, 2000; Wolff et al., 2022). In the following, we briefly introduce each GCM, draw important references to relevant constructs and psychometric approaches in psychedelic research, and mention considerations relevant to the development of the GCMQ.

#### Resource activation

The term *resources* broadly refers to all available means a person might use to pursue approach motives and satisfy their psychological needs. Resources include positive goals, related abilities and other positive attributes, positive beliefs about the world and self, possessions, and functioning relationships. In psychotherapy, resource activation is implemented through interventions that focus and build on the patient’s strengths and healthy qualities. Through resource activation, effective therapy fosters and utilizes the patient’s approach-motivational system as a driving force for therapeutic change (Flückiger et al., 2010b; Grawe, 1997, 2004).

In psychedelic therapy, effective implementation of resource activation during preparatory sessions is likely a prerequisite for patients to draw therapeutic benefits from subsequent dosing sessions. Conversely, there is evidence that psychedelic experiences can have resource-activating qualities. For example, psychedelic-occasioned peak experiences, including *mystical-type experiences* (Johnson et al., 2019; Yaden and Griffiths, 2021) and the closely related phenomenon of *oceanic boundlessness* (Dittrich, 1998; Majić et al., 2015) typically involve all hallmarks of resource-activating experiences (Wolff et al., under review): intense positive emotionality and absence of negative affect (indicating a strongly approach-motivated mode of mental functioning and concurrent inactivation of avoidance motives), satisfaction of all four basic psychological needs (orientation, attachment, pleasure, and self-esteem; Grawe, 2004), and positive appraisal of the world and self. Relatedly, certain types of psychedelic-occasioned insights, such as insights associated with positive views of the world and self or insights regarding positive personal goals or values, can be considered resource-activating experiences. Relevant psychometric instruments include the Mystical Experience Questionnaire (Barrett et al., 2015), the Oceanic Boundlessness (OBN) scale of the Altered States of Consciousness Questionnaire (Dittrich, 1998; Studerus et al., 2010), and the Goals and Adaptive Patterns Insights subscale of the Psychedelic Insight Questionnaire (PIQ; Davis et al., 2021). It should be noted, however, that not all resource-activating psychedelic experiences are necessarily related to the phenomena of peak experience, oceanic boundlessness, or psychological insight. Developing the GCMQ, we defined resource-activating experiences as all experiences characterized by an approach-motivated mode of mental functioning, including positive emotionality, positive appraisals of the world and self, and satisfaction of basic psychological needs.

#### Therapeutic relationship

Closely intertwined with the GCM resource activation, the therapeutic relationship is the most reliable predictor of treatment outcomes in psychotherapy (Baier et al., 2020; Flückiger et al., 2018; Norcross and Lambert, 2018; Orlinsky et al., 1994). An effective therapeutic relationship or *therapeutic alliance* comprises a positive emotional bond between the patient and therapist and mutual agreement on goals and tasks (Bordin, 1979; Horvath and Luborsky, 1993).

Although the therapeutic relationship has long been acknowledged as a fundamental aspect of psychedelic therapy (Abramson, 1967; Nayak and Johnson, 2020), psychedelic researchers have only recently started to investigate its role empirically. In a clinical trial testing psilocybin-assisted psychotherapy for depression, therapeutic alliance measured 1 day prior to dosing was found to predict post-treatment reductions in depression symptoms (Murphy et al., 2022). Consistent with the contextual-experiential model shown in Figure 1, the effect of the therapeutic alliance (i.e., a superordinate context factor) on treatment outcome was sequentially mediated via pre-dosing rapport (i.e., an immediate context factor) and emotional breakthrough experiences during dosing sessions, which, in turn, positively predicted the therapeutic alliance weeks later. Existing psychometric instruments (in this case, the Scale To Assess the Therapeutic Relationship; McGuire-Snieckus et al., 2007) can be used to measure the state of the alliance before or after dosing sessions. However, no instrument is yet available to measure retrospectively how a patient has experienced the therapeutic relationship while in the acute psychedelic state. To our knowledge, the GCMQ is the first instrument developed for this purpose. Given the commonly non-directive nature of therapist–patient interaction in psychedelic dosing sessions, and considering that direct interaction during these sessions is typically limited (as patients are commonly encouraged to assume an introspective state while wearing eyeshades and listening to music; Garcia-Romeu and Richards, 2018), the development of the GCMQ was intended to focus on the “positive emotional bond” aspect of the therapeutic alliance but not the “agreement on goals and tasks” aspect.

Furthermore, the GCMQ has been developed not only for clinical studies of psychedelic therapy but also for experimental studies with healthy volunteers and observational studies of naturalistic psychedelic use. In the latter case, there is evidence that the acute experience of one’s relationship with others who are present during a psychedelic experience can predict positive longer-term effects (Kettner et al., 2021). Here, we assume that relationship qualities central to the therapeutic relationship, such as interpersonal trust, connectedness, and support, also contribute to therapeutic and salutogenic effects of psychedelics that may occur outside of formal psychotherapy.

#### Problem actuation

Almost all conceptualizations of psychotherapy share the view that to overcome their problems, patients must directly experience these problems and the associated negative emotions (Frank, 1961; Grawe, 1997; Orlinsky et al., 1994). The GCM problem actuation corresponds to this principle. Problem actuation is assumed to exert therapeutic effects not by itself but only when implemented in synchrony with sufficient levels of resource activation (Gassmann and Grawe, 2006).

A related construct in psychedelic research is the *challenging experience* as operationalized by the Challenging Experience Questionnaire (CEQ; Barrett et al., 2016), which encompasses various affective, cognitive, and sensory aspects of distressful psychedelic experiences. However, there is no straightforward mapping between the challenging experience construct and the GCM problem actuation. First, in psychedelic and ordinary states of consciousness alike, distress and strong negative emotions do not always indicate that problematic aspects of the individual’s mental functioning are being actuated. Second, problem actuation is not necessarily always experienced as markedly challenging or distressful—especially when the associated negative emotions are met with openness and acceptance rather than avoidance (Wolff et al., 2020). The Acceptance/Avoidance-Promoting Experiences Questionnaire (APEQ; Wolff et al., 2022) was developed to assess complementary experiences related to avoidance and acceptance in psychedelic states. Developing the GCMQ, problem-actuating experiences were broadly defined as any experience characterized by negative emotions related to the individual’s psychological problems—irrespective of the experience of distress and irrespective of avoidance or acceptance.

#### Clarification and mastery

The two GCMs clarification and mastery represent closely related but distinguishable types of *corrective experiences* that are assumed to be catalyzed by synergistic interactions between the therapeutic relationship, resource activation, and problem actuation (Grawe, 1997, 2004; Grosse Holtforth and Flückiger, 2012). Clarification (also referred to as *motivational clarification* or *clarification of meaning*) denotes the process of gaining awareness and understanding of hitherto unconscious motives that determine the individual’s experience and behavior. Mastery refers to the concrete experience of learning to handle situations that were previously experienced as too difficult to handle or cope with (Grawe, 1997, 2004). This includes mastering or coping with both challenging external events and problematic mental events, such as intense negative emotions or thoughts.

A related construct in psychedelic research is the *emotional breakthrough experience* as operationalized by the Emotional Breakthrough Inventory (EBI; Roseman et al., 2019). Emotional breakthrough refers to experiences of catharsis or emotional release after facing and overcoming difficult emotional states in the psychedelic state. The emotional breakthrough construct overlaps with all problem-related GCMs, that is, problem actuation, clarification, and mastery. However, the EBI does not differentiate between these GCMs. Conceptually and empirically related to the EBI is the Acceptance scale of the mentioned APEQ (Wolff et al., 2022). This scale comprises subscales differentiating motivational, emotional, and cognitive aspects of acceptance-related psychedelic experiences. However, like the EBI, the APEQ Acceptance scale does not differentiate between clarification and mastery experiences. Two psychometric instruments that were recently developed for psychedelic research and that seem to correspond more specifically to the GCM clarification are the Psychedelic Insight Scale (Peill et al., 2022) and the Avoidance and Maladaptive Patterns Insights (AMP) subscale of the mentioned PIQ (Davis et al., 2021). By contrast, to our knowledge, there are still no psychometric instruments available to assess mastery experiences in the context of psychedelic states. However, psychedelic-occasioned mastery experiences involving emotional coping capabilities such as acceptance (Watts et al., 2017) and self-compassion (e.g., Renelli et al., 2020) have been described in the qualitative literature. Developing the GCMQ, we defined clarification experiences as any experience of gaining awareness and understanding of problem-related patterns of experience and behavior. Mastery experiences were defined as any experience associated with an increase in self-efficacy regarding one’s ability to handle problematic or difficult situations.

### Challenges in assessing GCMs in the context of psychedelic experiences

In psychotherapy research, session-to-session implementation of the five GCMs is commonly assessed using observer ratings of videotaped therapy sessions (e.g., Flückiger et al., 2009; Gassmann and Grawe, 2006) or questionnaires that can be administered to therapists or patients, such as the Scale for the Multiperspective Assessment of GCMs in Psychotherapy (SACiP; Mander et al., 2013) or the Bern Post Session Report (BPSR; Flückiger et al., 2010a). These instruments have limited utility for psychedelic research because the form of psychedelic dosing sessions differs from conventional talk therapy sessions. Following current standard protocols for dosing sessions, the patient (or research volunteer) is encouraged to spend most of the time—about 6 h in the case of psilocybin—in an introspective state while wearing eyeshades and listening to music over headphones. Although one or even two therapists are present for the entire session, verbal patient–therapist interaction is usually limited. Therefore, GCM-related experiences during these sessions are typically neither observable by the therapist nor from a third-person perspective. Observer or therapist ratings are thus of little use in this research context, whereas patient self-reports may be considered more valid. However, since existing self-report instruments were designed to assess GCMs in the context of conventional talk therapy sessions, items often describe experiences that are attributed to some patient–therapist interaction. The following SACiP items exemplify this issue:

Today, the therapist enabled me to view my problems in new contexts. (Item 4; clarification)

Today, the therapist touched my sore spots. (Item 9; problem actuation):

Today, the therapist intentionally used my abilities for therapy. (Item 17; resource activation)

Given that patient–therapist interaction is limited during psychedelic dosing sessions, patients may not necessarily attribute GCM-related experiences to such interactions with the therapist(s), limiting the applicability of existing instruments.

Another issue with existing instruments is that many items do not refer to GCM-related experiences themselves but rather describe post-session outcomes of such experiences, for example:

I have a better understanding of myself and my difficulties after today’s session. (Item 11; clarification)

I have the impression that my capacity to act improved by today’s session. (Item 21; mastery)

That these items refer to present outcomes rather than past experiences may be unproblematic for their use in psychotherapy research, where questionnaires are typically administered immediately following sessions. However, in psychedelic research, experiences of interest often date back hours, days, weeks, or (in the case of survey studies) even longer. A related issue with SACiP and BPSR items is that they often impose a specific frame of reference (e.g., “today”; “in today’s sessions”; “after today’s session”), thereby limiting the scope of possible research scenarios in which these instruments can be applied.

### The present study

The present study was conducted to develop the GCMQ, a self-report measure of GCM-related experiences designed to meet the specific requirements of clinical and nonclinical psychedelic research. To avoid the disadvantages associated with item translation and ensure maximum equivalence between the English and German versions (Tanzer, 2005), the GCMQ has been developed simultaneously in both languages. While we aimed to develop an instrument that could also be used in future clinical trials, this initial validation study was conducted as an online survey in a nonclinical sample of psychedelic users. Previous observational studies of naturalistic psychedelic use have yielded insights relevant to harm reduction and clinical applications of psychedelics, including insights into mechanisms of psychedelic-occasioned psychological change and the role of contextual conditions (e.g., Davis et al., 2020; Haijen et al., 2018; Nayak et al., 2023; Wolff et al., 2022). In addition to assessing the factor structure and psychometric properties of the GCMQ, we aimed to test the following hypotheses regarding associations between GCMs and several constructs that are commonly used to characterize psychedelic experiences:

- Emotional breakthrough experiences (EBI scores) and acceptance-related psychedelic experiences (APEQ Acceptance scores) are most strongly associated with the problem-related GCMs, that is, problem actuation, clarification, and mastery.
- Challenging experiences (CEQ total scores) and avoidance-related psychedelic experiences (APEQ Avoidance scores) are most strongly associated with the GCM problem actuation.
- Peak experiences (OBN scores) are most strongly associated with the GCM resource activation.

Furthermore, we aimed to test two sets of hypotheses derived from the contextual-experiential model (Figure 1): First, it is assumed that the occurrence of GCM-related psychedelic experiences depends on context factors that are conducive to such experiences. In the present study, this assumption is tested by assessing associations of GCMs with use motives and the settings in which psychedelic experiences occur. We hypothesize the following:

- Therapeutic use motives, such as using psychedelics to treat mental health problems, are positively associated with all five GCMs.
- Settings designed for therapeutic purposes are positively associated with all five GCMs.

Second, it is assumed that GCMs are essential mediators of psychedelic-occasioned psychological change. In the present study, limited by the cross-sectional and retrospective design, we approached this challenge by examining associations between GCMs, stressful life events (cumulated over the past 5 years), and mental well-being (at the time of data collection). This approach was inspired in part by preliminary evidence that using psychedelics with therapeutic intent is associated with lower levels of posttraumatic stress symptoms and internalized shame in individuals with a history of child maltreatment (Healy et al., 2021). We expect stressful life events to be negatively associated with mental well-being (Cohen et al., 2019). Moreover, based on the assumption that GCM-related psychedelic experiences can have therapeutic or resilience-enhancing effects, we expect that the association between stressful life events and well-being is moderated by all GCMs, except for problem actuation. The exemption of problem actuation is based on the view that this GCM alone cannot account for successful therapy but must be combined with thorough resource activation to exert therapeutic effects (Gassmann and Grawe, 2006; Mander et al., 2013). We hypothesize the following:

- The negative association between stressful life events and well-being is attenuated among individuals who report psychedelic experiences that are more strongly related to the GCMs resource activation, therapeutic relationship, mastery, and clarification.

## Method

### Development of the GCMQ

The GCMQ was designed to include five scales corresponding to the five GCMs introduced above. Instructions were formulated: “Here is a list of statements that may apply to your psychedelic experience. Please estimate the extent to which these statements apply to your experience.” A six-point Likert-type scale with verbal anchors at each point, (0) *“not at all”*; (1) *“a little”*; (2) *“moderately”*; (3) *“strongly”*; (4) *“very strongly”*; and (5) *“extremely,”* was chosen as the response format.

#### Item formulation

A pool of 44 candidate items was crafted by the authors of the present article following general recommendations for item formulation (Elson, 2017). Some items were inspired by SACiP (Mander et al., 2013) or BPSR items (Flückiger et al., 2010a). However, due to the abovementioned issues, no strong resemblance exists between GCMQ and SACiP or BPSR items. Each GCMQ item was specifically crafted for one of the five scales corresponding to the five GCMs. Item development was guided by the requirement that final versions of the GCMQ should apply to several distinct research scenarios: (1) observational studies of naturalistic psychedelic use (such as the present study), including various forms of recreational use, ceremonial or religious use, and therapeutic or self-medication use in various settings; (2) experimental studies that involve psychedelic dosing sessions with healthy volunteers; and (3) clinical studies of psychedelic therapy, including (3a) psychedelic dosing sessions (which often involve relatively little patient–therapist interaction) and (3b) non-psychedelic sessions such as preparatory and integration-focused psychotherapy sessions (which typically involve extensive patient–therapist interaction). Items for the scales Resource Activation (RA), Problem Actuation (PA), Clarification (CL), and Mastery (MA) were developed to apply to all of these research scenarios. Therefore, these items were not allowed to include references to a therapist or therapists. Items for the scale Relationship (RE) were developed in two versions: A version for clinical research scenarios (scenario 3 above) where at least one therapist is present and extensive patient–therapist interaction may occur (3b) or not occur (3a). Items for the clinical version of the Relationship scale were therefore designed to refer to the therapist(s). However, to apply to scenarios characterized by limited interaction, items were not allowed to include references to therapist behavior or behavioral patient-therapist interaction. For nonclinical research scenarios (scenarios 1 and 2 above), a modified nonclinical version of the Relationship scale was created by replacing the term *“my therapist(s)”* with the term *“the person(s) I was with”*. For instance, the Relationship (clinical) item *“I could trust my therapist(s)”* was reformulated as the Relationship (nonclinical) item *“I could trust the person(s) I was with.”*

All items were crafted, discussed, and revised simultaneously in English and German, following recommendations for simultaneous test development (Tanzer, 2005). The final items were reviewed and approved by a panel of six reviewers proficient in both English and German, including two native English speakers and four native German speakers. The pool of 44 candidate items is presented in the Supplemental Information (Tables S2–S6). Paper-and-pencil versions of the GCMQ in English and German, including clinical and nonclinical versions of the Relationship scale, are also provided in the Supplemental Information.

### Study procedure

The study was approved by the Institutional Review Board of Technische Universität Dresden (SR-EK-147032021). Participants were not compensated. Between August 2021 and April 2023, English- and German-speaking volunteers were invited to complete an anonymous cross-sectional online survey named “Survey on Psychedelic Experiences and Stressful Life Events” via invitations per email newsletters and social media posts. Invitations led to a landing page informing about the survey’s purpose in general terms, that is, “to improve our understanding of the acute and longer-term effects of psychedelics (. . .) focusing on the interplay between psychedelic experiences and stressful life events. Even if you have not experienced any particularly stressful events in the past, we very much welcome your participation.” From here, volunteers were directed to a SoSci-Survey (Leiner, 2019a) server hosted at Dresden University of Technology, Germany. The survey began with a request to choose between participation in English or German, followed by a consent form and assessment of inclusion criteria.

#### Inclusion criteria

To be allowed to complete the survey, participants had to (1) indicate a minimum age of 23 years, (2) endorse the ability to read, write, and speak the respective language fluently, (3) negate previous participation in the survey, and (4) endorse having felt at least once in the past 5 years discernible psychoactive effects following the intake of either LSD, psilocybin or psilocybin-containing mushrooms, mescaline or mescaline-containing cacti, or ayahuasca. Participants who fulfilled the inclusion criteria were allowed to complete the survey.

The minimum age of 23 years was chosen to ensure that the reported psychedelic experiences, which could date back a maximum of 5 years, were made in adulthood (at a minimum age of 18 years). The decision to focus the survey on the most commonly used longer-acting classical psychedelics was guided by the motive to include a reasonable proportion of participants who would report experiences that took place in therapeutic contexts (i.e., with therapeutic intentions and in settings designed for therapeutic purposes). The rationale for excluding shorter-acting classical psychedelics (e.g., inhaled N,N-dimethyltryptamine (DMT)) or atypical psychedelics (e.g., ketamine or 3,4-methylenedioxymethamphetamine (MDMA)) was that naturalistic use of these drugs presumably occurs less often in therapeutic contexts.

### Study measures

#### Demographics

Demographic information was collected by asking participants about their age, gender, and country of residence. The level of education was assessed using the Comparative Analysis of Social Mobility in Industrial Nations (CASMIN) classification (Brauns et al., 2003) to ensure comparability between the English- and German-speaking samples. For conciseness, the two groups are referred to as the English and German samples in the following.

#### Mental health and well-being

To obtain basic information regarding mental health, participants were asked whether they had ever been diagnosed with a mental disorder and, if so, to specify the type of disorder(s).

Mental well-being in the 2 weeks prior to survey participation was assessed using the Warwick-Edinburgh Mental Wellbeing Scale (WEMWBS; Tennant et al., 2007). The WEMWBS comprises 14 items and covers both hedonic and eudaimonic aspects of mental well-being, including positive affect, satisfying interpersonal relationships, and positive functioning. The WEMWBS exhibits high internal consistency (Cronbach’s α = 0.91; Tennant et al., 2007).

#### Cumulative stressful life events

Stressful life events in the past 5 years were assessed using a procedure adapted from Seery et al. (2010). Participants were presented with a list of 36 adverse events and were asked to indicate whether and how many times they had experienced each event in the past 5 years. The list covered various types of adverse events, including illness or injury, violence, discrimination, bereavement, social/environmental stress, relationship stress, and disaster. The complete list of events is provided in the Supplemental Information (Table S1). For each event type, up to four occurrences were counted. The total number of events counted per participant referred to as the cumulative stressful life events score, was treated as a continuous variable.

#### Report of a selected psychedelic experience

Participants were then asked to select one specific memorable psychedelic experience to report on in the remainder of the survey. Only experiences that the participant had undergone at least 1 month ago and no more than 5 years ago were allowed to be selected. After having selected an experience, participants were asked to report which psychedelic they had used, the route of consumption, time elapsed since the experience, the subjective clarity of their memory of the experience, subjective strength of the dose, subjective valence of the acute effects, retrospective appraisal of the experience, concomitant use of other psychoactive substances besides caffeine and nicotine, and approximate number of times having used classical psychedelics prior to the reported psychedelic experience.

##### Setting

To characterize the settings in which the reported experiences were undergone, participants were asked dichotomous (No/Yes) questions referring to specific setting categories (nature or close-to-nature setting; setting designed for a therapeutic purpose; religious, ceremonial, or spiritual setting; party, concert, or festival setting). Participants were then asked to rate the suitability of the setting *(“From today’s perspective, please rate how suitable you think this setting was for having a psychedelic experience.”)* on a five-point scale from 0 (*“not suited at all”*) to 4 (*“very well suited”*). To further characterize the setting, participants were asked to estimate the total number of people present during the experience and to indicate whether at least one supporting person was present (*“Was at least one person present whose task it was to support you during the experience, watch over you, or be there for you? This could be, for example, a trip sitter, a therapist, a shaman, etc.”*).

##### Use motives

Motives for psychedelic use were assessed by presenting participants with a list of 22 possible motives for using psychedelics (e.g., *“to treat psychological problems”; “to have fun,” “out of boredom”*) and asking them to rate the extent to which each item corresponded to their motives for undergoing the reported experience on a four-point Likert scale (*“not at all”; “somewhat”; “moderately”; “very much”*). In a previous survey study (Wolff et al., 2022), responses collected using the same method were suitable for principal component analysis (PCA), allowing the identification of three principal components named “therapeutic intention” (therapeutic use), “hedonic intention” (approach-motivated recreational use), and “escapist intention” (avoidance-motivated recreational use).

### Psychometric assessment of psychedelic experiences

#### General change mechanisms questionnaire

The psychometric assessment of participants’ reported psychedelic experiences began with the preliminary GCMQ, including English- and German-language versions of all 44 candidate items. Since a nonclinical sample was investigated here, the modified (nonclinical) Relationship scale was used instead of the standard Relationship scale. The Relationship scale was administered only to those participants who had indicated that a supporting person was present during their reported experience. The remaining four GCMQ scales (Resource Activation, Problem Actuation, Clarification, and Mastery) were administered to all participants.

#### Acceptance- and avoidance-related experience

The APEQ (Wolff et al., 2022) is a 32-item questionnaire designed to measure acceptance- and avoidance-related psychedelic experiences. The main scales, Acceptance (comprising the subscales Accepting Response, Relief, and Acceptance-Related Insights) and Avoidance (comprising the subscales Avoidant Response, Distress, and Avoidance-Related Insights), capture complementary motivational aspects of the psychedelic experience that are empirically largely independent from each other (Wolff et al., 2022). Furthermore, the APEQ includes two ancillary scales named Introspection (measuring introspective mental states and internally focused attention) and Interaction (measuring interaction with the environment and externally focused attention). The main scales, Acceptance and Avoidance (12 items each), and the ancillary scales, Introspection and Interaction (4 items each), were used in the present study. Internal consistency was excellent for the Acceptance scale (Cronbach’s α = 0.92 in both the English and German sample) and the Avoidance scale (0.91 and 0.93, respectively), good for the Introspection scale (0.85 and 0.88, respectively), and acceptable for the Interaction scale (0.76 and 0.79, respectively).

#### Emotional breakthrough experience

The EBI is a 6-item questionnaire designed to measure the “phenomenon of overcoming challenging emotions/memories and thereby experiencing emotional release or breakthrough” during psychedelic experiences (Roseman et al., 2019). Besides the original English version, we used a German translation that showed high internal consistency in a previous survey study (Wolff et al., 2022). The EBI showed excellent internal consistency in the present study (Cronbach’s α = 0.92 and 0.91 in the English and German samples, respectively).

#### Challenging experience

The CEQ (Barrett et al., 2016; German translation by Dworatzyk et al., 2021) was developed as a multidimensional measure of aversive and psychologically challenging psychedelic experiences. The CEQ comprises 26 items, seven subscales (Fear, Grief, Physical Distress, Insanity, Isolation, Death, and Paranoia), and a total scale. The total scale was used in the present study. Internal consistency of the CEQ total scale was excellent (Cronbach’s α = 0.95 in both the English and German samples).

#### Peak experience

The current version of the Altered States of Consciousness Questionnaire, which consists of 11 subscales (11-ASC; Studerus et al., 2010), is among the most commonly used self-rating instruments to quantify subjective drug effects. In the present survey, participants were administered only those 14 (out of 42) 11-ASC items that belong to the higher-level scale Oceanic Boundlessness (OBN; comprising the four lower-level scales Experience of Unity, Spiritual Experience, Blissful State, and Insightfulness). Internal consistency of the OBN scale was excellent (Cronbach’s α = 0.93 in both the English sample and the German sample).

### Data analysis

#### Characteristics of participants and reported psychedelic experiences

We removed observations that were deemed invalid due to (1) speeding (Leiner, 2019b), (2) using more than one psychedelic during the reported experience, (3) unusual (non-oral) route of consumption (i.e., smoked, inhaled, insufflated, injected, or other), (4) responding to the free-entry feedback request at the end of the survey in ways that raised concerns regarding the validity of reports, (5) reporting on an experience that took place longer than 5 years ago, or (6) indicating poor memory of the reported experience. Characteristics of the remaining participants and their reported psychedelic experiences were then described and compared between the English and German samples.

#### PCA of use motives

Following the same data analysis strategy that was used in previous survey studies (Haijen et al., 2018; Wolff et al., 2022), we used PCA with orthogonal rotation (Varimax) to examine the factor structure underlying reported use motives in the complete cross-language sample of included English- and German-speaking participants. Component scores were then extracted to be entered as independent variables in subsequent regression analyses testing hypotheses regarding associations between use motives and GCMs.

#### Confirmatory factor analyses of GCMQ items

Confirmatory factor analyses (CFAs) were used to select the final GCMQ items (item selection) and assess the factor structure of the GCMQ (model selection and model replication). To account for expected deviations from normality in GCMQ item scores, CFAs were calculated using the robust maximum likelihood (MLR) estimator in Mplus 8.9. Model fit was assessed by evaluating multiple fit indices and comparing models with simpler nested models. Following recommendations by Brown (2015), the Root-Mean Square Error of Approximation (RMSEA), the Confirmatory Fit Index (CFI), and the standardized root mean square residual (SRMR) were calculated as fit indices. Scaled *χ*^2^ difference tests (Satorra and Bentler, 2001) were used for nested model comparisons.

Since each GCMQ item was specifically designed for one theory-derived scale, item selection and model selection were based solely on CFA and involved no exploratory factor analysis (EFA). However, following item/model selection and replication, EFAs were calculated to further explore the factor structure of the final GCMQ items in the total sample and the subsample of experiences that occurred in therapeutic settings.

##### Matched strata for independent item/model selection and model replication

Because the English sample was substantially larger than the German sample, the English sample was used for both item/model selection and subsequent model replication. The German sample was used for model replication only. To obtain independent participant samples for item/model selection and model replication, the English sample was stratified into two sub-samples matched on the type of psychedelic used, subjective dose strength, and presence of a supporting person during the reported experience. The following automated stratification procedure was carried out in Matlab R2022a: Observations from each cell of the factorial model assumed by the stratification variables were randomly assigned in equal parts to a “selection stratum” and a “replication stratum.” To avoid unnecessary discarding of valid data, residual observations from cells containing odd numbers of observations were grouped together and randomly assigned in equal parts to the two strata. Confirming the validity of the stratification procedure, *χ*^2^ independence tests showed no significant differences in the stratification variables between the selection and replication stratum.

##### Item selection

To ensure the applicability of the GCMQ to future research scenarios with high demands for parsimony (e.g., psychometric batteries administered after psychedelic dosing sessions in clinical studies), we decided a priori that the final questionnaire should comprise no more than five items for each of the five scales, that is, 25 items in total. To select the final five items for each scale, separate CFAs, each including one factor and all candidate items of the respective scale, were calculated for the selection stratum. In the following item selection process, preference was given to items with relatively high factor loadings, although no specific cutoff was defined. Furthermore, item selection was guided by the aim that each scale should cover the theoretical construct of the given GCM as broadly as possible. Following item selection, a second CFA was calculated to assess model fit, this time only including the five selected items of the respective scale.

##### Model selection and replication

To test the complete model, a CFA including all selected items and five factors corresponding to the GCMs was calculated for the selection stratum. This baseline model was then compared to more constrained alternative models, and scaled *χ*^2^ difference tests (Satorra and Bentler, 2001) were used to select the most parsimonious model. The selected measurement model was then replicated by repeating the same CFAs in the independent replication stratum of the English sample and the independent German sample.

#### Structural equation models

Hypothesized associations between GCMs, context factors (use motives and settings), various characteristics of participants’ reported psychedelic experiences (introspection and interaction; avoidance- and acceptance-related experience; emotional breakthrough experience; challenging experience; peak experience), stressful life events, and current well-being were examined in the total cross-language sample by calculating structural equation models (SEMs).

##### Associations with context factors

First, the selected and replicated measurement model was calculated for the total (cross-language) sample. The measurement model was then extended to a SEM by regressing the five GCM factors on component scores extracted from the PCA of use motives. Furthermore, a series of separate SEMs regressing the GCM factors on the categorical setting variables was calculated.

##### Moderation of the association between stressful life events and well-being

To examine the hypothesized moderating effects of GCMs on the association between stressful life events and mental well-being, another SEM was calculated separately for each GCM, regressing WEMWBS scores on the respective GCM factor, cumulative stressful life events scores, and the latent interaction term between the GCM factor and stressful life events scores.

#### Correlation analyses

To examine associations with other psychometric scales, correlations between GCMQ scales and APEQ scales, EBI scores, CEQ total scores, and OBN scores were calculated in the total (cross-language) sample.

#### Factor mixture models

Subtypes of GCM-related experiences were explored using factor mixture modeling in Mplus 8.9. Factor mixture models are a combination of CFA with latent profile analysis that incorporates continuous latent variables (latent factors) and categorical latent variables (latent profiles) to identify and characterize distinct subgroups of observations within a dataset (Lubke and Muthén, 2005). Factor mixture models with increasing numbers of latent profiles were calculated based on the selected measurement model for the total cross-language sample. All parameters except the factor means were constrained equally across latent profiles to ensure comparability. Several criteria were inspected to select an appropriate number of latent profiles. The resulting profiles were then characterized and compared based on participants’ assigned class membership.

## Results

### Participants

Of the 4621 volunteers who agreed to participate, 2002 fulfilled the inclusion criteria and completed the survey. Out of these, 135 volunteers were excluded for one or more of the following reasons: 61 volunteers indicated a non-oral route of consumption; 39 volunteers indicated having used more than one psychedelic during their reported experience; 19 volunteers reached scores ⩾2 on the TIME_RSI speeding index; 13 volunteers indicated that their reported psychedelic experience had taken place more than 5 years ago; 10 volunteers indicated that their memory of the reported experience was “not clear at all”; five volunteers’ free-entry responses to the feedback request at the end of the survey raised concerns regarding the validity of their reports.

Of the 3482 volunteers who provided demographic data (age, gender, education level) at the beginning of the survey, 1615 dropped out before completing the survey or were excluded for the reasons mentioned above. Compared to these volunteers, the final sample of 1867 included participants was slightly older (*t*(3,480) = 1.973, *p* = 0.049, Cohen’s *d* = 0.067) and slightly more educated (*χ*^2^(2) = 22.653, *p* < 0.001, Cramer’s *V* = 0.081).

Characteristics of the final sample of 1867 participants (1153 in the English sample and 714 in the German sample) are presented in Table 1. Comparisons between the English and German samples are provided in the Supplemental Information (Table S8). Significant differences between the English and German samples were found for several characteristics, and moderate effect sizes were found for age, level of education, lifetime diagnosis of any mental disorder, lifetime diagnosis of anxiety disorder, cumulative stressful life events, and current well-being. Participants in the English sample reported 62 different countries of residence, and the most frequent mentions were the USA (46.8%), the UK (8.2%), Germany (7.8%), Canada (6.3%), Belgium (2.4%), the Netherlands (2.3%), Australia (2.3%), France (1.9%), Poland (1.6%), Denmark (1.1%), Italy (1.1%), Sweden (1.1%), Finland (1.0%), Ireland (1.0%), South Africa (1.0%), Spain (1.0%), and Switzerland (1.0%). Participants in the German sample reported 12 different countries of residence, and the most frequent mentions were Germany (88.2%), Switzerland (5.6%), and Austria (4.3%). All participants endorsed the ability to read, write, and speak the respective language fluently.

**Table 1.** Characteristics of included participants and comparisons by assigned class membership.

	Total (cross-language) sample (*N* = 1867)	Profile 1: Moderately therapeutic experience (*n* = 560)	Profile 2: Problem-focused experience (*n* = 133)	Profile 3: Resource-focused experience (*n* = 513)	Profile 4: Non-therapeutic experience (*n* = 306)	Profile 5: Highly therapeutic experience (*n* = 355)	*F* or *χ*^2^	*p*	*η2* or Cramer’s *V*
Mean (SD) age	33.5 (9.7)	33.5 (9.5)	32.7 (10.5)	34.0 (10.0)	33.1 (9.9)	33.2 (9.4)	0.824	0.510	0.002
Gender							37.871	<0.001	0.101
Male	64.3%	66.4%	57.1%	59.5%	75.5%	61.1%			
Female	32.5%	30.9%	36.1%	38.0%	23.2%	33.8%			
Other	3.2%	2.7%	6.8%	2.5%	1.3%	5.1%			
CASMIN classification of education level							7.299	0.505	0.044
Tertiary education (highest)	77.0%	78.0%	75.2%	78.2%	72.2%	78.6%			
Secondary education	20.7%	20.4%	22.6%	19.5%	25.2%	18.6%			
Primary education (lowest)	2.2%	1.6%	2.3%	2.3%	2.6%	2.8%			
Lifetime diagnosis of mental disorder	56.1%	57.50%	62.4%	51.5%	52.3%	61.4%	12.940	0.012	0.083
Depression	39.3%	40.7%	46.6%	34.3%	34.3%	45.6%	17.978	<0.001	0.098
Anxiety disorder	25.7%	25.4%	32.3%	19.3%	23.5%	34.9%	30.246	<0.001	0.128
ADHD	15.7%	16.1%	13.5%	13.8%	14.7%	19.7%	6.411	0.170	0.059
PTSD	12.9%	13.0%	19.5%	9.2%	7.5%	20.0%	35.546	0.001	0.138
Addiction	8.9%	8.8%	7.5%	8.2%	7.2%	12.4%	7.064	0.133	0.062
Mania	3.0%	3.4%	7.5%	1.2%	2.0%	4.2%	18.507	<0.001	0.100
Psychosis	1.9%	2.5%	1.5%	1.9%	0.3%	2.3%	5.568	0.234	0.055
Other	11.1%	11.1%	13.5%	9.6%	10.1%	13.2%	3.987	0.408	0.048
*M* (SD) cumulative stressful life events	9.0 (8.8)	9.5 (8.3)	10.3 (9.6)	7.4 (6.7)	7.0 (6.8)	11.8 (11.0)	19.424	<0.001	0.040
*M* (SD) well-being (WEMWBS)	50.0 (8.8)	50.1 (7.9)	44.5 (9.7)	51.8 (7.8)	46.9 (9.7)	52.1 (8.8)	34.901	<0.001	0.070

ADHD: attention deficit hyperactivity disorder; CASMIN: Comparative Analysis of Social Mobility in Industrial Nations (Brauns et al., 2003); PTSD: post-traumatic stress disorder; WEMWBS: Warwick-Edinburgh Mental Wellbeing Scale.

### Characteristics of reported psychedelic experiences

Characteristics of the psychedelic experiences reported by included participants are summarized in Table 2. Comparisons between the English and German samples are provided in the Supplemental Information (Table S9). Experiences reported by the English and German sample differed significantly in several characteristics. Except for the type of psychedelic used, for which a moderate effect size was found, all of these comparisons showed only small effect sizes.

**Table 2.** Characteristics of reported psychedelic experiences and comparisons by assigned class membership.

	Total (cross-language) sample (*N* = 1867)	Profile 1: Moderately therapeutic experience (*n* = 560)	Profile 2: Problem-focused experience (*n* = 133)	Profile 3: Resource-focused experience (*n* = 513)	Profile 4: Non-therapeutic experience (*n* = 306)	Profile 5: Highly therapeutic experience (*n* = 355)	*F* or *χ*^2^	*p*	*η2* or Cramer’s *V*
*M* (SD) months elapsed since experience	17.2 (17.2)	16.2 (17.0)	20.6 (17.9)	17.9 (17.3)	16.5 (18.0)	17.0 (16.4)	2.102	0.078	0.004
Subjective clarity of memory^a^							30.892	0.002	0.074
Completely clear	25.2%	26.3%	21.8%	25.7%	19.3%	29.0%			
Very clear	43.3%	42.9%	38.3%	44.8%	39.9%	46.8%			
Clear	23.2%	22.5%	30.8%	22.6%	28.4%	17.7%			
Somewhat clear	8.3%	8.4%	9.0%	6.8%	12.4%	6.5%			
Psychedelic used							87.329	<0.001	0.125
LSD	45.7%	48.8%	51.1%	48.9%	43.5%	36.3%			
Psilocybin or psilocybin-containing mushrooms	44.2%	40.9%	38.3%	45.0%	52.9%	43.1%			
Ayahuasca	8.6%	8.4%	10.5%	4.7%	2.9%	18.9%			
Mescaline or mescaline-containing cacti	1.4%	2.0%	0.0%	1.4%	0.7%	1.7%			
Subjective dose strength							63.439	<0.001	0.092
Low	4.2%	3.6%	3.0%	4.1%	7.8%	2.5%			
Moderate	36.7%	37.0%	37.6%	38.8%	42.8%	27.9%			
High	38.4%	37.9%	39.8%	42.5%	30.7%	39.2%			
Very high	15.8%	15.7%	12.8%	12.3%	15.4%	22.5%			
Extremely high	4.9%	5.9%	6.8%	2.3%	3.3%	7.9%			
Valence of acute effects							509.817	<0.001	0.302
Rather pleasant	54.5%	50.4%	6.8%	78.4%	63.7%	36.3%			
Rather unpleasant	5.1%	2.3%	33.8%	0.8%	7.2%	3.4%			
Both pleasant and unpleasant	38.5%	45.5%	58.6%	20.5%	25.2%	57.5%			
Neither pleasant nor unpleasant	1.9%	1.8%	0.8%	0.4%	3.9%	2.8%			
Retrospective appraisal							461.548	<0.001	0.249
Very positive	64.2%	67.7%	28.6%	77.2%	35.9%	77.7%			
Positive	27.5%	28.6%	29.3%	21.8%	43.5%	19.4%			
Neutral	5.2%	2.7%	21.1%	0.8%	14.1%	2.0%			
Negative	2.4%	0.9%	15.0%	0.2%	5.9%	0.3%			
Very negative	0.7%	0.2%	6.0%	0.0%	0.7%	0.6%			
Concomitant substance use									
None	59.6%	60.0%	59.4%	58.7%	55.9%	63.4%	4.083	0.395	0.047
Cannabis	29.9%	30.5%	25.6%	31.6%	30.4%	27.6%	2.918	0.571	0.040
Alcohol	10.9%	10.0%	13.5%	11.5%	16.0%	6.2%	17.890	<0.001	0.098
Entactogens	3.7%	3.9%	8.3%	3.1%	4.2%	2.0%	11.613	0.020	0.079
Dissociatives	3.0%	3.2%	3.0%	3.5%	3.6%	1.4%	4.008	0.405	0.046
Stimulants	2.4%	2.0%	2.3%	2.5%	3.3%	2.3%	1.515	0.824	0.028
Benzodiazepines	0.7%	0.4%	2.3%	0.2%	0.7%	1.4%	10.085	0.039	0.073
Opiates/opioids	0.7%	0.5%	0.0%	1.2%	1.0%	0.3%	4.043	0.400	0.047
Other psychoactive substance(s)	2.2%	1.8%	1.5%	1.9%	2.3%	3.4%	3.211	0.533	0.041
Use motives (component scores)^b^									
* M* (SD) therapeutic intention	0.00 (1.00)	0.26 (0.89)	−0.36 (0.95)	−0.08 (0.90)	−0.78 (0.98)	0.52 (0.86)	104.44	<0.001	0.183
* M* (SD) hedonic intention	0.00 (1.00)	−0.04 (1.02)	−0.31 (1.06)	0.33 (0.92)	0.00 (0.92)	−0.29 (0.99)	26.34	<0.001	0.054
* M* (SD) escapist intention	0.00 (1.00)	−0.01 (0.99)	0.51 (1.24)	−0.26 (0.86)	0.14 (0.96)	0.08 (1.04)	20.14	<0.001	0.041
Setting categories									
Nature or close-to-nature setting	57.6%	57.1%	48.9%	67.4%	46.7%	56.6%	39.494	<0.001	0.145
Setting designed for therapeutic purpose	13.4%	13.0%	15.0%	7.4%	4.2%	29.9%	121.279	<0.001	0.255
Ceremonial, religious, or spiritual setting	11.4%	11.6%	9.8%	9.0%	4.2%	21.4%	54.044	<0.001	0.170
Party, concert, or festival	10.4%	11.1%	14.3%	12.7%	10.8%	4.5%	18.469	<0.001	0.099
Retrospective suitability of setting							167.876	<0.001	0.150
Very well suited	45.0%	44.3%	21.1%	53.4%	32.7%	53.5%			
Well suited	34.8%	36.1%	30.1%	34.7%	38.9%	31.3%			
Somewhat suited	14.3%	15.2%	26.3%	10.1%	18.0%	11.3%			
Hardly suited	4.2%	3.0%	15.0%	1.8%	6.5%	3.4%			
Not suited at all	1.7%	1.4%	7.5%	0.0%	3.9%	0.6%			
Presence of other people							72.288	<0.001	0.098
0 (alone)	29.4%	31.3%	27.8%	25.3%	28.4%	33.5%			
1–5 people	55.2%	53.6%	51.1%	62.2%	61.1%	43.9%			
6–15 people	8.9%	9.3%	10.5%	7.0%	5.9%	13.2%			
16–30 people	2.9%	1.8%	4.5%	1.9%	1.3%	7.0%			
31–100 people	1.2%	1.4%	3.8%	1.0%	1.0%	0.6%			
>100 people	2.4%	2.7%	2.3%	2.5%	2.3%	1.7%			
Presence of supporting person(s)	37.4%	38.0%	41.4%	31.6%	28.4%	51.0%	46.916	<0.001	0.159
Psychedelic use prior to reported experience							39.742	0.005	0.073
0 (never used before)	16.3%	15.0%	18.8%	15.8%	19.6%	15.2%			
1–5 times	26.1%	23.6%	33.8%	27.7%	31.4%	20.6%			
6–20 times	23.8%	24.5%	21.1%	25.5%	19.0%	25.4%			
21–50 times	15.3%	17.7%	15.8%	11.9%	16.0%	15.8%			
51–100 times	8.8%	9.6%	5.3%	9.4%	5.9%	10.4%			
>100 times	9.7%	9.6%	5.3%	9.7%	8.2%	12.7%			

a Ten volunteers indicated their memory of the reported experience was “not clear at all”. These volunteers were excluded; hence, frequencies for the response option “not clear at all” are not reported here.

b Component scores were extracted from the principal component analysis (PCA) reported in Table 3.

#### Use motives

Bartlett’s test of sphericity (*χ*^2^(231) = 11,517.466; *p* < 0.001) and the Kaiser–Mayer–Olkin measure of sampling adequacy (0.829) indicated that participants’ responses to the use motives items were suitable for PCA. Six components with eigenvalues greater than 1 were found, but the scree plot suggested that a three-component solution was most appropriate. The three components cumulatively explained 43.1% of the variance. Based on the loadings listed in Table 3, which overall were consistent with previous results (Wolff et al., 2022), the components were named (1) “therapeutic intention,” (2) “hedonic intention,” and (3) “escapist intention.” Component scores of English- and German-speaking participants differed significantly (Supplemental Table S9): On average, the English sample exhibited more pronounced therapeutic (*t*(1,865) = 9.114; *p* < 0.001; Cohen’s *d* = 0.437) and escapist intentions (*t*(1, 865) = 6.895; *p* < 0.001; Cohen’s *d* = 0.334] and less pronounced hedonic intentions (*t*(1,865) = −5.431; *p* < 0.001; Cohen’s *d* = −0.258).

**Table 3.** Item loadings from principal component analysis (PCA) of use motives.

Item	Component 1: Therapeutic intention	Component 2: Hedonic intention	Component 3: Escapist intention
For self-awareness	**0.733**	−0.074	−0.185
For personal growth	**0.727**	−0.150	−0.191
To increase my well-being	**0.668**	−0.012	0.152
To confront difficult feelings	**0.635**	−0.371	0.126
For spiritual reasons	**0.612**	0.074	−0.269
For performance enhancement	**0.589**	0.088	0.166
To treat psychological problems	**0.589**	−0.362	0.303
For religious reasons	**0.371**	0.092	−0.148
To treat physical problems	**0.361**	−0.149	0.140
To have fun	−0.269	**0.770**	0.205
To spend time with friends	−0.193	**0.674**	−0.025
For partying	−0.192	**0.591**	0.242
For relaxation	0.213	**0.579**	0.270
To have an experience of nature	0.329	**0.529**	−0.193
To intoxicate myself	−0.252	**0.523**	0.353
To increase my creativity	0.486	**0.522**	−0.011
To increase sexual pleasure	0.219	**0.378**	0.150
Out of curiosity	−0.055	**0.296**	−0.043
To distract myself from problems	0.030	0.109	**0.799**
To escape from difficult feelings	0.213	−0.144	**0.742**
Out of boredom	−0.076	0.255	**0.562**
To fit in	−0.126	0.160	**0.238**

Items were rated on a four-point Likert scale (“not at all”; “somewhat,” “moderately,” “very much”). The highest loading of each item is written in bold font.

### General change mechanisms questionnaire

#### Item score distributions

Descriptive statistics for candidate items are provided in the Supplemental Information (Tables S2–S7). Several item scores were mildly non-normally distributed, with univariate skewness ranging from −1.31 to 1.47 and kurtosis ranging from −1.38 to 1.30. Two Relationship items showed univariate outliers (i.e., *z*-scores < −3.29) for 1.1% (RE01) and 0.8% of responses (RE02), respectively. Mahalanobis distance procedures (with *p* < 0.001) classified 141 participants (7.6% of the sample) as multivariate outliers. Upon inspection, none of these participants showed clear signs of careless responding, and a majority (63.8%) entered meaningful responses to optional free-text items presented at the end of the survey, in many cases providing detailed accounts of their experiences. Furthermore, the 141 multivariate outliers were unevenly distributed across the five latent profiles identified via factor mixture modeling (*χ*^2^(4) = 20.381, *p* < 0.001, Cramer’s *V* = 0.104), and the largest proportion of outliers (17.3%) was found in the smallest class (Profile 2). Thus, distributions from multivariate normality likely reflect the existence of distinct subgroups of experiences associated with meaningful differences in response patterns. Multivariate outliers were therefore retained for further analyses. All CFAs, SEMs, and factor mixture models were calculated with the robust maximum likelihood (MLR) estimator in Mplus 8.9 to account for nonnormality in the dataset.

#### Item selection

**Figure 2.**
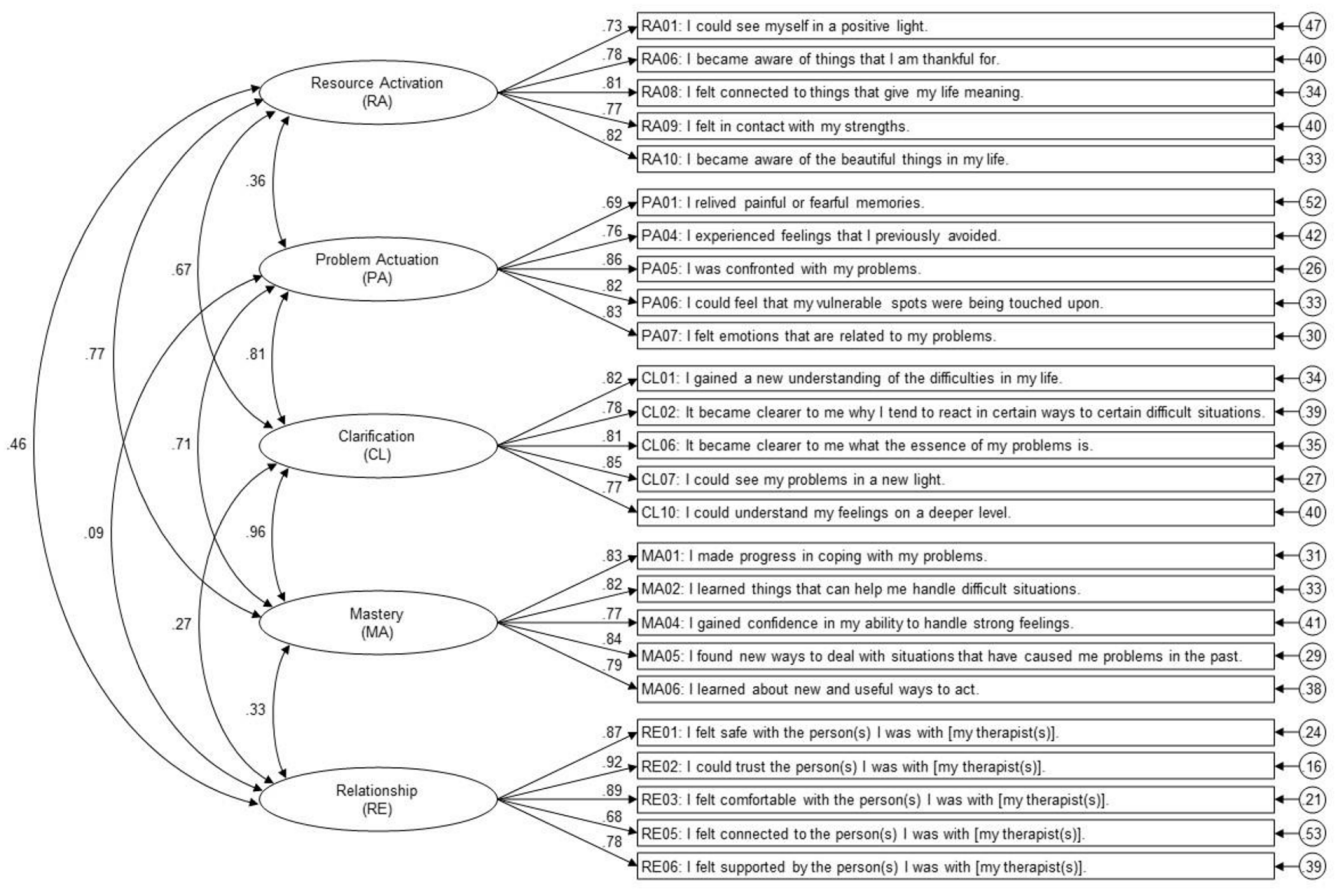
Summary of the selected measurement model in the total (cross-language) sample (*N* = 1867). The wording of the clinical version of the Relationship (RE) scale, which was not used in the present study, is written in square brackets. Paper-and-pencil versions of the GCMQ in English and German, including the final order of items to be used in future studies, are provided in the Supplemental Materials.

Factor loadings and model fit indicators for the CFAs conducted for item selection in the selection stratum of the English sample are summarized in the Supplemental Information (Tables S2–S7). After item selection, model fit was good for all five scales. The final selection of 25 items is presented in Figure 2.

#### Model selection and replication

Summaries of all models calculated for model selection and replication are provided in the Supplemental Information (Figures S1–S3). Table 4 provides model fit indices and model comparisons. First, a CFA including the 25 selected GCMQ items and five factors corresponding to the five scales/GCMs was calculated for the selection stratum of the English sample. This baseline model showed acceptable fit and was replicated with acceptable fit in the independent replication stratum of the English sample and the independent German sample. As expected, correlations between the problem-related GCMs (Problem Actuation, Clarification, and Mastery) were the strongest inter-factor correlations in all baseline models. The five-factor baseline model was therefore compared to two more constrained alternative models: (1) A four-factor model where Clarification and Mastery were collapsed into one single factor and (2) a three-factor model where Problem Actuation, Clarification, and Mastery were collapsed. The four-factor model showed an acceptable fit in all samples. The three-factor model showed poor fit in all samples. Compared to the five-factor baseline model, the more constrained models’ fit to the data was significantly worse in all samples, indicating that the factors Problem Actuation, Clarification, and Mastery were statistically distinguishable. The baseline model with five separate factors was therefore selected for further analyses. Figure 2 summarizes the selected five-factor model in the complete cross-language sample. Since the four-factor model also showed an acceptable fit in all samples, combining the two separable scales Clarification and Mastery into one superordinate scale named “Corrective Experience” appears to be justified.

**Table 4.** Model fit indices and model comparisons.

Sample/model	Model fit						Model fit compared to the baseline model		
	*χ* ^2^	*df*	*p*	RMSEA	CFI	SRMR	*χ* ^2^_*diff*_	*df*	*p*
Selection stratum of English sample (*n* = 577)									
Five-factor (baseline) model	878.5	265	<0.001	0.063	0.923	0.078			
Four-factor model (CL and MA collapsed)	923.9	269	<0.001	0.065	0.918	0.080	40.8	4	<0.001
Three-factor model (PA, CL, and MA collapsed)	1418.9	272	<0.001	0.085	0.856	0.092	426.5	7	<0.001
Replication stratum of English sample (*n* = 576)									
Five-factor (baseline) model	895.0	265	<0.001	0.064	0.920	0.068			
Four-factor model (CL and MA collapsed)	964.4	269	<0.001	0.067	0.912	0.071	48.7	4	<0.001
Three-factor model (PA, CL, and MA collapsed)	1523.3	272	<0.001	0.089	0.842	0.092	381.5	7	<0.001
German sample (*n* = 714)									
Five-factor (baseline) model	956.5	265	<0.001	0.060	0.922	0.069			
Four-factor model (CL and MA collapsed)	1075.6	269	<0.001	0.065	0.909	0.073	104.5	4	<0.001
Three-factor model (PA, CL, and MA collapsed)	1900.7	272	<0.001	0.092	0.816	0.100	745.0	7	<0.001
Total (cross-language) sample (*N* = 1867)									
Five-factor (baseline) model	1982.8	265	<0.001	0.059	0.929	0.065			
Four-factor model (CL and MA collapsed)	2200.5	269	<0.001	0.062	0.920	0.068	174.2	4	<0.001
Three-factor model (PA, CL, and MA collapsed)	4010.4	272	<0.001	0.086	0.846	0.089	1481.9	7	<0.001

CFI: comparative fit index; CL: Clarification; MA: Mastery; PA: Problem Actuation; RMSEA: root mean square error of approximation; SRMR: standardized root mean residual.

In the Supplemental Information, we summarize EFAs of the final 25 GCMQ items in the total sample (Table S10) and the subsample of experiences that occurred in therapeutic settings (Table S11).

#### GCMQ scores in the English and German sample

Descriptive statistics and internal consistencies for the GCMQ scales are shown in Table 5. Compared to the German sample, the English sample scored significantly higher on all scales, including the superordinate Corrective Experience scale. The effect size was small for the Relationship scale and moderate for all other scales.

**Table 5.** GCMQ scores and internal consistencies of scales in the English (*n* = 1153) and German samples (*n* = 714).

Scale	*M* (SD)		*t*	*p*	Effect size (Cohen’s *d*)	Cronbach’s α (McDonald’s *ω*)	
	English sample	German sample				English sample	German sample
Resource Activation (RA)	3.17 (1.35)	2.86 (1.27)	4.871	<0.001	0.232	0.90 (0.90)	0.87 (0.87)
Problem Actuation (PA)	2.21 (1.49)	1.86 (1.40)	4.952	<0.001	0.236	0.89 (0.90)	0.89 (0.90)
Clarification (CL)	2.74 (1.42)	2.34 (1.33)	6.052	<0.001	0.288	0.91 (0.91)	0.89 (0.89)
Mastery (MA)	2.48 (1.42)	2.08 (1.30)	6.176	<0.001	0.294	0.91 (0.91)	0.89 (0.89)
Corrective Experience (CL and MA combined)	2.61 (1.37)	2.21 (1.26)	6.336	<0.001	0.302	0.95 (0.95)	0.94 (0.94)
Relationship (RE)	3.79 (1.14)	3.62 (0.95)	2.039	0.021	0.159	0.92 (0.92)	0.89 (0.89)

### Structural equation models

#### Associations between GCMs and context factors

To examine associations with use motives, the selected measurement model summarized in Figure 2 was extended to an SEM by regressing the five GCM factors on component scores extracted from the PCA of use motives. This model is summarized in Table 6. As hypothesized, therapeutic intention was positively associated with all five GCMs. The hedonic intention was positively associated with resource activation but negatively associated with problem actuation, clarification, and mastery. Escapist intention was positively associated with problem actuation but negatively associated with resource activation and the relationship factor.

**Table 6.** Summary of structural equation model regressing general change mechanism (GCM) factors on use motives.

Regressor	*β* (*p*)				
	Resource Activation	Problem Actuation	Clarification	Mastery	Relationship
Therapeutic intention	0.506 (<0.001)	0.375 (<0.001)	0.521 (<0.001)	0.543 (<0.001)	0.197 (<0.001)
Hedonic intention	0.089 (<0.001)	−0.239 (<0.001)	−0.161 (<0.001)	−0.139 (<0.001)	0.052 (0.145)
Escapist intention	−0.177 (<0.001)	0.131 (<0.001)	0.019 (0.385)	−0.039 (0.083)	−0.142 (0.002)

Component scores extracted from the principal component analysis (PCA) of use motives reported in Table 3 were entered as regressors in a single model. Regression weights are standardized.

SEMs regressing the GCM factors on setting categories are summarized in Table 7. As hypothesized, there was a strong positive association between all five GCMs and settings designed for a therapeutic purpose. Ceremonial, religious, or spiritual settings were positively associated with all GCMs except the Relationship factor. Nature or close-to-nature settings were positively associated with Resource Activation, negatively associated with Problem Actuation, and not significantly associated with the other GCMs. Party, concert, or festival settings were negatively associated with all GCMs except the relationship factor.

**Table 7.** Summary of separate structural equation models regressing general change mechanism (GCM) factors on setting categories.

Model/regressor	*β* (*p*)				
	Resource Activation	Problem Actuation	Clarification	Mastery	Relationship
Nature or close-to-nature setting	0.297 (<0.001)	−0.098 (0.048)	−0.042 (0.403)	0.002 (0.969)	0.089 (0.255)
Setting designed for therapeutic purpose	0.378 (<0.001)	0.754 (<0.001)	0.659 (<0.001)	0.653 (<0.001)	0.211 (0.010)
Ceremonial, religious, or spiritual event	0.388 (<0.001)	0.566 (<0.001)	0.448 (<0.001)	0.538 (<0.001)	−0.020 (0.820)
Party, concert, or festival	−0.248 (0.002)	−0.305 (<0.001)	−0.411 (<0.001)	−0.387 (<0.001)	0.000 (0.998)

Categorical (0/1) variables corresponding to participants’ dichotomous responses (No/Yes) to setting items were entered as regressors in separate models. Regression weights are unstandardized.

#### Moderation of the association between stressful life events and well-being

**Figure 3.**
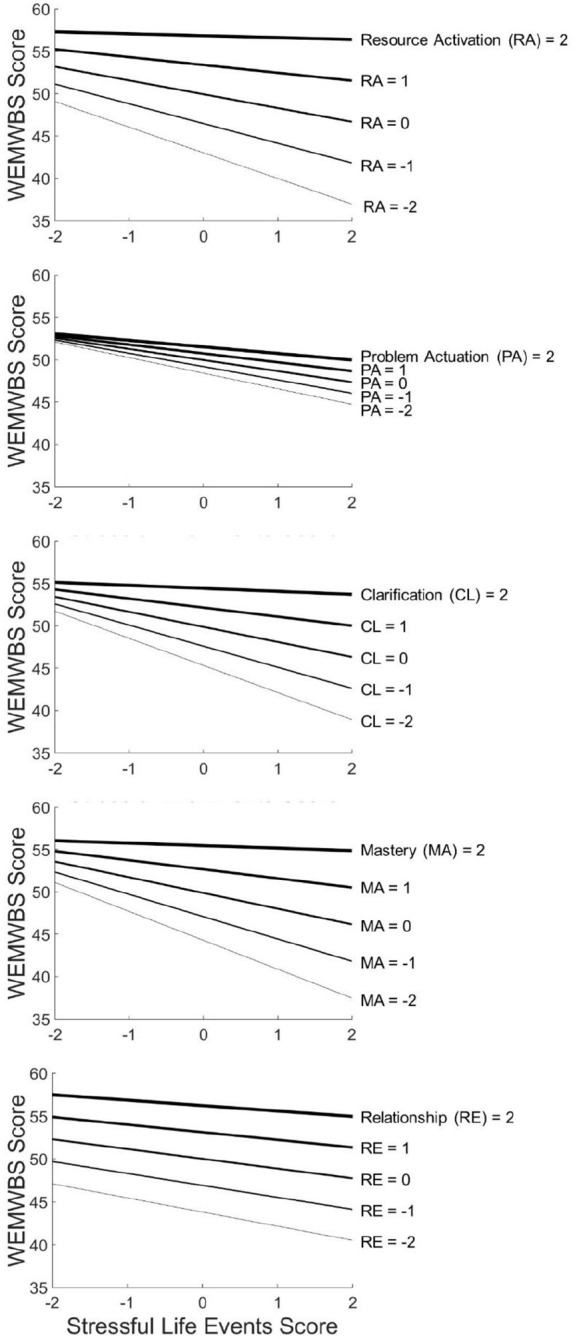
Interaction plots illustrating associations between (standardized) cumulative stressful life events over the past 5 years, current mental well-being (WEMWBS scores), and the five general change mechanism (GCM) factors as estimated by the structural equation models (SEMs) summarized in Table 9. Different levels of each (standardized) GCM factor are shown as plots of varying thickness, with thicker plots representing more strongly GCM-related psychedelic experiences. The overall negative effect of stressful life events on well-being was significantly moderated by the latent factors Resource Activation (RA), Clarification (CL), and Mastery (MA) but not by the latent factors Problem Actuation (PA) and Relationship (RE).

SEMs regressing WEMWBS scores on GCM factors, cumulative stressful life events scores, and the latent interaction term between the respective GCM and stressful life events are summarized in Table 8. Figure 3 shows interaction plots illustrating the same results. As hypothesized, stressful life events were negatively associated with well-being, and all GCM factors were positively associated with well-being. Also as hypothesized, the association between stressful life events and well-being was significantly moderated by Resource Activation, Clarification, and Mastery, but not by Problem Actuation. Contrary to our hypothesis, the Relationship factor did not significantly moderate the association between stressful life events and well-being.

**Table 8.** Summary of separate structural equation models regressing mental well-being (WEMWBS scores) on general change mechanism (GCM) factors and cumulative stressful life events scores.

Model/regressor	*β* (*p*)
Resource Activation (RA)	
Intercept	49.936 (<0.001)
Cumulative stressful life events	−1.627 (<0.001)
RA	3.458 (<0.001)
RA * cumulative stressful life events	0.700 (0.007)
Problem Actuation (PA)	
Intercept	49.969 (<0.001)
Cumulative stressful life events	−1.305 (<0.001)
PA	0.776 (0.001)
PA * cumulative stressful life events	0.266 (0.266)
Clarification (CL)	
Intercept	49.875 (<0.001)
Cumulative stressful life events	−1.778 (<0.001)
CL	2.277 (<0.001)
CL * cumulative stressful life events	0.711 (0.006)
Mastery (MA)	
Intercept	49.872 (<0.001)
Cumulative stressful life events	−1.851 (<0.001)
MA	2.794 (<0.001)
MA * cumulative stressful life events	0.779 (0.002)
Relationship (RE)	
Intercept	50.018 (<0.001)
Cumulative stressful life events	−1.142 (<0.001)
RE	3.106 (<.001)
RE * cumulative stressful life events	0.254 (0.411)

Regression weights are unstandardized, but cumulative stressful life events were standardized, and the scale of RA, PA, CL, MA, and RE was determined by fixing the mean and variance of these factors at zero and one, respectively. Hence, these variables can be treated as standardized.

### Correlations between the GCMQ and other psychometric scales

Correlations between GCMQ scales, APEQ main scales (Acceptance and Avoidance), APEQ ancillary scales (Introspection and Interaction), EBI scores, CEQ total scores, and OBN scores are presented in Table 9. These correlations were largely as hypothesized: APEQ Acceptance was positively correlated with all five GCMQ scales. APEQ Avoidance and CEQ total scores were most strongly positively correlated with the GCMQ scale Problem Actuation. APEQ Introspection was positively correlated with all GCMQ scales (whereas APEQ Interaction showed a mixed pattern of correlations). EBI scores were most strongly positively correlated with the GCMQ scales Problem Actuation, Clarification, and Mastery. 11-ASC OBN scores were most strongly positively correlated with the GCMQ scale Resource Activation.

**Table 9.** Correlations among psychometric scales were used to assess reported psychedelic experiences in the total cross-language sample.

	*r* (*p*)										
	1.	2.	3.	4.	5.	6.	7.	8.	9.	10.	11.
1. GCMQ RA	—										
2. GCMQ PA	0.32 (<0.001)	—									
3. GCMQ CL	0.61 (<0.001)	0.73 (<0.001)	—								
4. GCMQ MA	0.70 (<0.001)	0.63 (<0.001)	0.86 (<0.001)	—							
5. GCMQ RE	0.47 (<0.001)	0.12 (0.002)	0.30 (<0.001)	0.35 (<0.001)	—						
6. APEQ Acceptance	0.65 (<0.001)	0.61 (<0.001)	0.78 (<0.001)	0.79 (<0.001)	0.32 (<0.001)	—					
7. APEQ Avoidance	−0.15 (<0.001)	0.45 (<0.001)	0.18 (<0.001)	0.09 (<0.001)	−0.20 (<0.001)	0.09 (<0.001)	—				
8. APEQ Introspection	0.40 (<0.001)	0.52 (<0.001)	0.57 (<0.001)	0.52 (<0.001)	0.13 (0.001)	0.61 (<0.001)	0.21 (<0.001)	—			
9. APEQ Interaction	0.20 (<0.001)	−0.13 (<0.001)	−0.02 (0.514)	0.01 (0.631)	0.17 (<0.001)	0.06 (0.015)	−0.03 (0.246)	−0.18 (<0.001)	—		
10. EBI	0.51 (<0.001)	0.70 (<0.001)	0.76 (<0.001)	0.73 (<0.001)	0.23 (<0.001)	0.82 (<0.001)	0.19 (<0.001)	0.55 (<0.001)	−0.07 (0.003)	—	
11. CEQ	−0.16 (<0.001)	0.50 (<0.001)	0.19 (<0.001)	0.10 (<0.001)	−0.27 (<0.001)	0.09 (<0.001)	0.75 (<0.001)	0.26 (<0.001)	−0.19 (<0.001)	0.22 (<0.001)	—
12. 11-ASC OBN	0.67 (<0.001)	0.27 (<0.001)	0.48 (<0.001)	0.53 (<0.001)	0.27 (<0.001)	0.59 (<0.001)	−0.03 (0.284)	0.46 (<0.001)	0.15 (<0.001)	0.47 (<0.001)	−0.02 (0.438)

11-ASC OBN: Oceanic Boundlessness scale of the Altered States of Consciousness Questionnaire; APEQ: Acceptance/Avoidance-Promoting Experiences Questionnaire; CEQ: Challenging Experience Questionnaire; CL: Clarification; EBI: Emotional Breakthrough Inventory; GCMQ: General Change Mechanisms Questionnaire; MA: Mastery; PA: Problem Actuation; RA: Resource Cctivation; RE: Relationship.

### Latent profiles of GCM-related experiences

**Figure 4.**
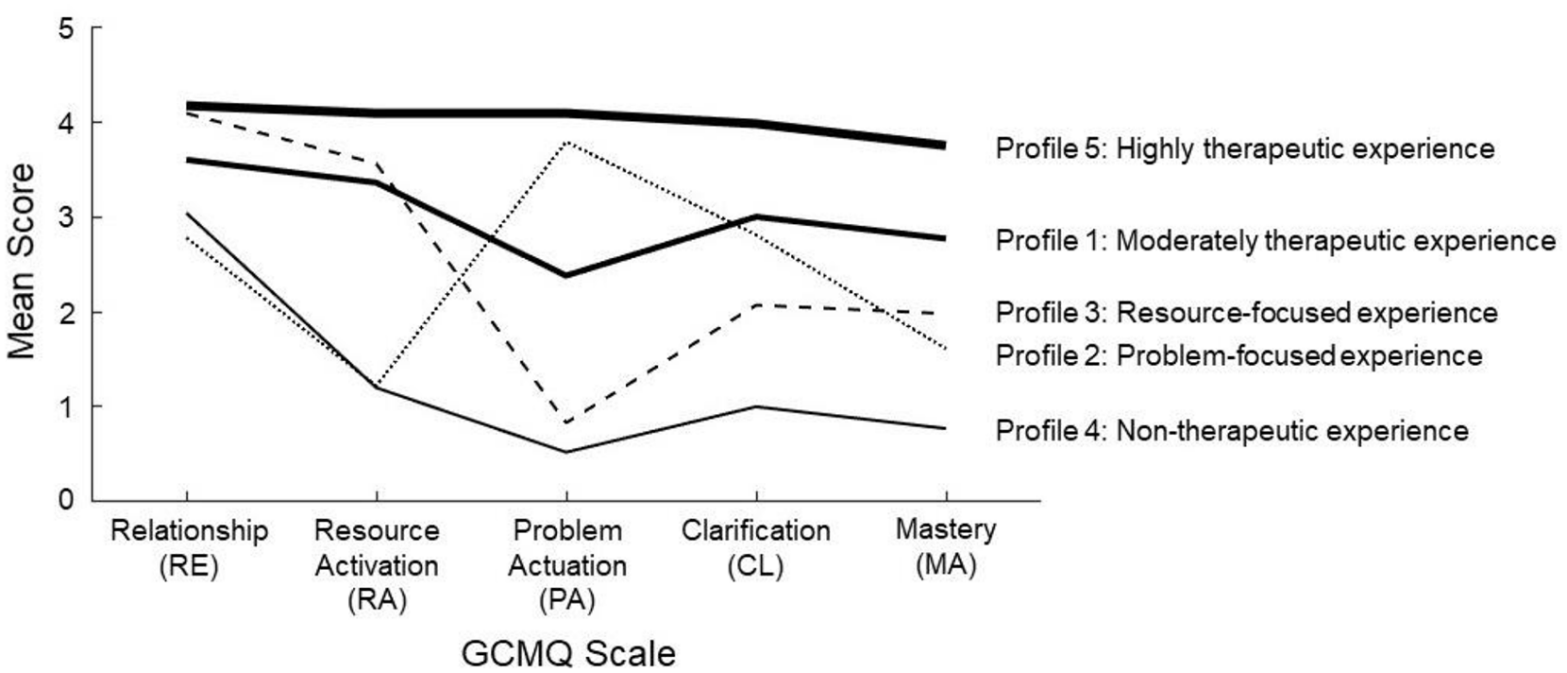
Illustration of the five profiles of general change mechanism (GCM)-related experiences identified via factor mixture modeling. The illustration is based on participants’ assigned class membership and observed GCMQ scores. Estimated factor means are provided in the Supplemental Information (Table S12).

Table 10 summarizes the criteria inspected to select a factor mixture model with an appropriate number of latent profiles. Whereas the Akaike information criterion (AIC) and Bayesian information criterion (BIC) provided no clear indication, the Lo–Mendell–Rubin test (LMRT) indicated that adding a sixth profile did not yield a superior model. The five-profile solution was therefore selected as the most appropriate model. Based on participants’ assigned class membership, the five classes included 560 (30.0%), 133 (7.1%), 513 (27.5%), 306 (16.3%), and 355 participants (19.0%), respectively. Figure 4 shows GCMQ scores for each latent profile based on assigned class membership. Considering these distinct patterns, the latent profiles were named (1) “moderately therapeutic experience,” (2) “problem-focused experience,” (3) “resource-focused experience,” (4) “non-therapeutic experience,” and (5) “highly therapeutic experience.” The Supplemental Information provides estimated means for the latent GCM factors (Table S12) and a psychometric characterization of the profiles, including GCMQ, APEQ, EBI, CEQ, and 11-ASC OBN scores (Table S13). The identified profiles differed in terms of various context factors. Notable differences in participant characteristics (Table 1) include mental disorders, stressful life events, and well-being. Differences regarding the reported experiences (Table 2) include drug factors (substance; dose), external context factors (settings), and internal context factors (use motives). A multinomial logistic model regressing assigned class membership on use motives and setting categories explained 29.6% of the variance in class membership according to Nagelkerke’s pseudo-*R*^2^. Odds ratios estimated by multinomial logistic regression with the reference class Profile 1 (moderately therapeutic experience) are shown in Table 11. Odds ratios with the other profiles as reference class are provided in the Supplemental Information (Tables S14–S17).

**Table 10.** Criteria inspected for factor mixture model selection.

Model	AIC	BIC	LMRT *p*	Entropy	Smallest class
1 profile	86859.19	87329.42	—	—	—
2 profiles	86588.25	87091.67	<0.001	0.75	38.6%
3 profiles	86410.12	86964.73	<0.001	0.81	4.6%
4 profiles	86239.52	86809.33	<0.001	0.76	7.3%
5 profiles	86161.69	86764.69	0.020	0.75	7.1%
6 profiles	86083.98	86720.17	0.372	0.78	4.2%

AIC: Akaike information criterion; BIC: Bayesian information criterion; LMRT: Lo–Mendell–Rubin test.

**Table 11.** Odds ratios estimated by multinomial logistic regression of assigned class membership (compared to the reference class profile 1—moderately therapeutic experience) on context factors.

Predictor	Profile 2: Problem-focused experience		Profile 3: Resource-focused experience		Profile 4: Non-therapeutic experience		Profile 5: Highly therapeutic experience	
	OR (95% CI)	*p*	OR (95% CI)	*p*	OR (95% CI)	*p*	OR (95% CI)	*P*
Use motives								
Therapeutic intention	0.46 (0.37; 0.58)	<0.001	0.64 (0.55; 0.74)	<0.001	0.30 (0.25; 0.36)	<0.001	1.28 (1.08; 1.52)	0.005
Hedonic intention	0.77 (0.62; 0.96)	0.021	1.54 (1.34; 1.76)	<0.001	1.10 (0.92; 1.30)	0.301	0.85 (0.73; 0.99)	0.032
Escapist intention	1.56 (1.31; 1.85)	<0.001	0.75 (0.65; 0.87)	<0.001	1.11 (0.95; 1.29)	0.201	1.13 (0.98; 1.30)	0.097
Setting categories								
Nature or close-to-nature setting	0.98 (0.65; 1.48)	0.907	1.23 (0.94; 1.61)	0.133	0.77 (0.56; 1.06)	0.103	1.05 (0.78; 1.40)	0.751
Setting designed for therapeutic purpose	1.80 (0.95; 3.39)	0.071	0.79 (0.50; 1.26)	0.324	0.74 (0.38; 1.47)	0.392	2.13 (1.45; 3.12)	<0.001
Ceremonial, religious, or spiritual setting	0.91 (0.44; 1.92)	0.808	1.05 (0.67; 1.65)	0.827	0.70 (0.35; 1.40)	0.310	1.27 (0.83; 1.94)	0.277
Party, concert, or festival	0.96 (0.51; 1.80)	0.887	0.62 (0.41; 0.93)	0.020	0.43 (0.26; 0.72)	0.001	0.56 (0.31; 1.03)	0.064

CI: confidence interval; OR: odds ratio.

## Discussion

The present study describes the theory-based development and initial validation of a new psychometric instrument to assess GCM-related psychotherapeutic or psychotherapy-like processes in the context of psychedelic experiences. Besides evaluating the factor structure and psychometric properties of the GCMQ, we examined hypothesized associations between GCMs and context factors (use motives and setting categories) and identified five distinguishable types of GCM-related psychedelic experiences. We also examined the hypothesized moderating effects of GCMs on the association between stressful life events and mental well-being. Furthermore, to demonstrate convergent validity, we assessed associations between GCMQ scores and other scales that are commonly used to characterize psychedelic experiences in current psychedelic research.

### Factor structure and psychometric properties

The theorized factor structure of the GCMQ, including five factors corresponding to the GCMs resource activation, problem actuation, clarification, mastery, and therapeutic relationship, was first confirmed in an English-speaking sample that was used to select the final items for each scale (selection stratum). The factor structure was then independently confirmed in a separate English-speaking sample (replication stratum) and a German-speaking sample. CFAs showed a high degree of convergence between these three samples. This suggests that the present results are fairly generalizable and indicates that the simultaneously developed English and German versions of the GCMQ are equivalent.

Although strong correlations between the “problem-related” scales Problem Actuation, Clarification, and Mastery were found, the five-factor model with separate factors for these scales consistently showed significantly better fit to the data compared to a more constrained four-factor model (collapsing Clarification and Mastery into one single factor) and a three-factor model (collapsing Problem Actuation, Clarification, and Mastery). These results confirm the theory-based distinction between the five GCMs and justify maintaining Problem Actuation, Clarification, and Mastery as separate scales. The three-factor model showed poor model fit, indicating that Problem Actuation, Clarification, and Mastery items should not be combined into one superordinate scale. The necessity to keep Problem Actuation as a separate scale is further supported by several distinctive associations of the Problem Actuation factor with context factors (use motives and settings) and potential longer-term outcomes (well-being; see discussion below).

In contrast to the three-factor model, the four-factor model still showed an acceptable fit, indicating that clarification and mastery are not only separable but also unifiable constructs. The strong association found between the factors Clarification and Mastery is also plausible from a theoretical point of view: Clarification experiences (i.e., gaining insights into problems) and mastery experiences (i.e., gaining abilities for coping with problems) constitute distinguishable but closely related “corrective experiences” that share similar prerequisites and often promote one another (Grawe, 2004, 2007; Grosse Holtforth and Flückiger, 2012). Therefore, based on empirical and theoretical considerations, it seems justified to derive a superordinate scale (named “Corrective Experience”) combining Clarification and Mastery items. This is in line with standard practices in psychotherapy research, where clarification and mastery scales are sometimes combined into a single measure (e.g., Gassmann and Grawe, 2006; Moggia et al., 2023; Rubel et al., 2017; Wrede et al., 2023). However, distinguishing between clarification and mastery may be necessary for certain types of GCM-related psychedelic experiences: Whereas Clarification and Mastery scores were approximately equal for the other types of experiences, problem-focused experiences showed moderate levels of clarification but low levels of mastery (Figure 4; see discussion below).

All five GCMQ scales and the superordinate scale Corrective Experience demonstrated good-to-excellent internal consistency in the English and German samples.

### Convergent validity

Correlations between GCMQ scales and other scales designed to assess psychedelic experiences provided evidence for convergent validity. As expected, EBI and APEQ Acceptance scores were positively correlated with all five GCMs. As hypothesized, emotional breakthrough experiences (EBI scores) were most strongly associated with the problem-related GCMs, that is, problem-actuation, clarification, and mastery. Acceptance-related experiences (APEQ Acceptance scores) were similarly correlated with these GCMs. Acceptance was also relatively strongly correlated with the GCM resource activation (*r* = 0.65), suggesting that tolerance of strong negative emotions requires an approach-motivated mode of mental functioning (Grawe, 2004). “Acceptance-based” models of psychotherapy, such as Acceptance and Commitment Therapy (ACT; Hayes et al., 2020; Luoma et al., 2019), accommodate this necessity by purposefully balancing problem- and resource-focused interventions (i.e., acceptance vs commitment/values). Relatedly, causal links between (resource-activating) peak experiences and acceptance-related psychedelic experiences have been proposed (Wolff et al., 2020).

Also as hypothesized, peak experiences (OBN scores) were most strongly correlated with the GCM resource activation. This is consistent with the view that peak experiences (including mystical-type experiences and experiences of oceanic boundlessness) are often resource-activating. However, the moderate strength of the correlation (*r* = 0.66) also indicates that not all strongly resource-activating psychedelic experiences are peak experiences, nor vice versa. Aspects of psychedelic-occasioned resource activation that are associated with but not bound to the peak experience phenomenon include positive emotionality (McCulloch et al., 2022) and resource-related insights (Davis et al., 2021).

Finally, as hypothesized, challenging psychedelic experiences (CEQ total scores) and (highly correlated; *r* = 0.75) avoidance-related experiences (APEQ Avoidance scores) were positively and more strongly associated with problem actuation than with the other GCMs. The moderate strength of these correlations (*r* = 0.50 and *r* = 0.44, respectively) is consistent with the view that problem-actuating psychedelic experiences are not necessarily always experienced as challenging or distressful—especially when these experiences are met with acceptance rather than avoidance (Wolff et al., 2020, 2022). In line with this, acceptance-related experiences (APEQ Acceptance scores) were also moderately correlated with problem actuation (*r* = 0.61) but only negligibly correlated with CEQ scores (*r* = 0.09).

Exploratory analyses showed that introspective states (APEQ Introspection scores) were positively correlated with all five GCMs. By contrast, interaction with the environment (APEQ Interaction scores) was positively correlated with resource activation and the relationship factor but negatively correlated with problem actuation. This likely reflects the corresponding differences between introspective and more interactive settings (e.g., therapeutic versus nature or close-to-nature settings) discussed below.

### Associations between GCM-related psychedelic experiences and context factors

#### The role of use motives

Following a previous survey with *N* = 1829 participants (Wolff et al., 2022), the present study with *N* = 1867 participants is the second to demonstrate the relevance of therapeutic, hedonic, and escapist use motives for the therapeutic quality of psychedelic experiences. To our knowledge, these two studies comprise the most comprehensive datasets on motives for psychedelic use available to date (for a systematic review, see Basedow and Kuitunen-Paul, 2022). In the present study, as hypothesized based on the contextual-experiential model (Figure 1), therapeutic intentions were positively associated with all five GCMs. This result is consistent with the established finding from psychotherapy research that patients’ treatment goals have a decisive influence on the therapeutic process (DeFife and Hilsenroth, 2011; Michalak and Holtforth, 2006; Wollburg and Braukhaus, 2010). Hedonic intentions were also positively associated with resource activation but negatively associated with the problem-related GCMs, that is, problem actuation, clarification, and mastery. By contrast, escapist intentions were negatively associated with resource activation and the relationship factor but positively associated with problem actuation. Thus, considering that problem actuation without concurrent resource activation is relatively unlikely to yield corrective experiences of clarification or mastery (Flückiger et al., 2009; Gassmann and Grawe, 2006; Grawe, 1997, 2004), escapist use motives can be considered unfavorable from a psychotherapeutic perspective. This view is consistent with the idea that approach-motivated psychedelic use often leads to experiences characterized by positive affectivity, whereas avoidance-motivated use tends to entail challenging experiences (Wolff et al., 2020, 2022). While the present results still need to be replicated in longitudinal and clinical studies, they provide support for the common practice of discussing intentions when preparing patients for psychedelic dosing sessions or providing harm-reduction to psychedelic users (Pilecki et al., 2021; Thal et al., 2022b). A sensible therapeutic approach that seems to be supported by the present results is to reduce avoidance motives (e.g., “I want to get rid of unpleasant feelings”; “I want to leave my problems behind”) and foster approach motives (e.g., “I want to connect more with other people”; “I want to understand my problems better”) when setting intentions for an upcoming psychedelic experience.

#### The role of different settings

The contextual-experiential model (Figure 1) was further supported by the finding that different psychedelic use settings were associated with GCM-related experiences in different ways. As hypothesized, settings designed for a therapeutic purpose were positively associated with all five GCMs. This association was especially strong for the problem-related GCMs, that is, problem actuation, clarification, and mastery. Ceremonial, religious, or spiritual settings were also positively associated with the problem-related GCMs and (less strongly) with resource activation. By contrast, party, concert, or festival settings were negatively associated with these GCMs. This suggests that psychotherapeutic or psychotherapy-like psychedelic experiences also occur outside of explicitly therapeutic contexts but may require a psychosocial context that, like psychotherapy, encourages introspective attention, acceptance of negative emotions, and interpersonal trust and support.

Of potential relevance to the future development of psychedelic therapies, nature or close-to-nature settings were positively associated with resource activation but negatively associated with problem actuation. This result points to promising research avenues related to evidence-based practices established by empirical psychotherapy research: For problem-actuating interventions to have therapeutic effects and lead to corrective experiences, they should be implemented based on activated resources (Flückiger et al., 2009; Gassmann and Grawe, 2006; Grawe, 1997, 2004). Therefore, in psychotherapy, it is recommended to emphasize resource-activating interventions before focusing on patients’ problems. As yet, the practical application of such evidence-based principles to psychedelic dosing sessions is difficult because the intense acute effects that high-dose psychedelics typically have on patients can substantially limit therapists’ ability to target specific GCMs using process-directive verbal interventions. Against this backdrop, the systematic exploration of different therapeutic settings could lead to more feasible approaches for targeting therapeutic processes during psychedelic interventions (Carhart-Harris et al., 2018). The use of nature settings in psychedelic therapy has been proposed (Gandy et al., 2020) but remains to be tested experimentally. The present results suggest that future clinical studies could explore a sequential approach: In the early therapy process or the early phase of dosing sessions, nature or close-to-nature settings could be used to induce resource-activating psychedelic experiences. At later stages, more introspective settings conforming to current standard protocols (i.e., indoors, lying down, wearing eyeshades and headphones; Garcia-Romeu and Richards, 2018) could be introduced to induce more problem-actuating experiences. Based on a similar rationale, sequential variations of therapeutic technique could be explored which, within one continuous setting, bring about a gradual transition from interaction to introspection. Such studies, which are inspired by applying established insights of empirical psychotherapy research to the special case of psychedelic therapy, could use the GCMQ to test whether experimental manipulations of context factors exert the intended effects on the therapy process.

#### A typology of GCM-related psychedelic experiences

Although various measures of specific aspects of psychedelic experiences exist, no empirically based typology of therapeutically relevant psychedelic experiences has been proposed to date. Here, we used factor mixture modeling to identify five distinct profiles of GCM-related psychedelic experiences (Figure 4). Consistent with the underlying theoretical model (Grawe, 1997, 2004), these profiles confirm that GCMs are best understood as interrelated processes. Pointing to the potential usefulness of a GCM-based typology for research and clinical practice, the present results provide broad evidence of associations between distinguishable types of GCM-related experiences and modifiable context factors such as use motives and setting factors. Based on the contextual-experiential model (Figure 1), we assume that the identified types of experiences are likely also predictive of longer-term outcomes, such as different patterns of symptom change. This should be tested in future longitudinal and clinical studies. If replicated in clinical studies and shown to have predictive value, the identified typology could inform the development of personalized treatment strategies and enhance the scientific understanding of both therapeutic and harmful effects of psychedelics.

##### Highly therapeutic experiences

Highly therapeutic experiences were characterized by high levels of all five GCMs. Our results provide extensive evidence that such experiences are closely linked with the intentionally therapeutic use of psychedelics: First, highly therapeutic experiences were relatively often reported by participants with a lifetime diagnosis of a mental disorder (affective disorder, anxiety disorder, or PTSD). Furthermore, these experiences were associated with therapeutic use motives. They also occurred relatively often (but far from exclusively) in settings designed for therapeutic purposes, ceremonial settings, and settings where a supporting person was present. Correspondingly, highly therapeutic experiences were relatively often occasioned by ayahuasca, that is, a psychedelic that is often used in a ritualistic manner, in carefully designed settings, and with therapeutic intentions (Perkins et al., 2021). Further emphasizing the context dependence of highly therapeutic experiences, the majority of them (84.7%) occurred in settings that participants deemed “well suited” or “very well suited” for psychedelic use. High associated levels of introspection suggest that these experiences depend on prolonged introspective states. In line with this, the relatively high levels of both avoidance and acceptance associated with this profile may suggest that highly therapeutic experiences often involve a process of learning to accept and engage with unpleasant emotions that the individual initially attempts to avoid (“learning to let go”; Wolff et al., 2020). Compared to the other identified profiles, highly therapeutic experiences were associated with higher levels of well-being despite also higher numbers of stressful life events. While this result must be interpreted with caution due to the cross-sectional and retrospective design of the present study, it is consistent with the idea that these experiences can exert therapeutic or resilience-enhancing effects.

##### Moderately therapeutic experiences

Moderately therapeutic experiences were also characterized by relatively high levels of all five GCMs. Compared to highly therapeutic experiences, all GCMs—especially problem actuation—were less pronounced. Multinomial logistic regression revealed that, compared to highly therapeutic experiences, these experiences were associated with less therapeutic use motives and more hedonic use motives. Settings not designed for therapeutic purposes were also associated with an increased likelihood of reporting a moderately rather than highly therapeutic experience. Further notable differences include smaller doses of psychedelics, more concomitant substance use, less presence of supporting persons, less introspection, and less acceptance. These results are consistent with the view that willingness and preparedness to confront unpleasant problem-related emotions are key factors determining whether an experience will be strongly or only moderately therapeutic.

##### Resource-focused experiences

Resource-focused experiences were characterized by a combination of high levels of resource activation with low levels of problem actuation. Associated high levels of hedonic intention, as well as low levels of escapist intention, suggest that these experiences tend to occur in strongly approach-motivated modes of mental functioning. Consistent with this interpretation, resource-focused experiences were overwhelmingly (99.0%) regarded as “positive” or “very positive” and were associated with particularly low levels of avoidance, low levels of challenging experience, and low numbers of cumulative stressful life events. Resource-focused experiences occurred particularly often in nature settings and relatively rarely in settings designed for therapeutic purposes. A large majority of participants (88.1%) rated the setting as “well suited” or “very well suited.” Relatively, low levels of introspection and high levels of interaction may suggest that resource-focused experiences are promoted by active social exchange and engagement with positively valued external stimuli (e.g., as encountered in suitable nature settings). Participants assigned to this class reported relatively high levels of current well-being. This association may be explained by a tendency to access positive memories when in a positive mood (recall bias), but is also consistent with the view that resource-focused experiences can have positive longer-term effects on well-being.

##### Problem-focused experiences

Contrary to resource-focused experiences, problem-focused experiences were characterized by a combination of low levels of resource activation with high levels of problem actuation. These experiences were associated with particularly high levels of escapist intention and low levels of therapeutic and hedonic intention, suggesting that they tend to occur in strongly avoidance-motivated modes of mental functioning. Further supporting the contextual-experiential model (Figure 1), a comparatively large proportion of problem-focused experiences (48.8%) occurred in settings that participants deemed only “somewhat suited,” “hardly suited,” or even “not suited at all” for psychedelic use. Relatively high levels of concomitant benzodiazepine use suggest that participants tended to use these tranquilizing substances, when available, to mitigate or prevent anxiety or other forms of distress. Entactogens, which were also relatively often used in the context of problem-focused experiences, may have been used for similar purposes. On the other hand, entactogen use may have causally contributed to the occurrence of problem-focused experiences. Of note, co-administration of the entactogen MDMA has been hypothesized to decrease psychedelic-induced anxiety but did not do so in a recent experimental study (Straumann et al., 2023; Zeifman et al., 2023). Although problem-focused experiences were characterized as disproportionately avoidance-related and challenging, they were still often (57,9%) regarded as “positive” or “very positive.” These positive appraisals may be due to the fact that problem-focused experiences also showed fairly high levels of clarification. Together with the observed low levels of mastery, this suggests that problem-focused experiences can often be described as overwhelmingly confronting and, at least temporarily, overextending the individual’s coping abilities—but still potentially insightful. However, participants who reported problem-focused experiences exhibited relatively poor well-being, perhaps indicating that these experiences exert only limited therapeutic effects or may even cause psychological harm. This interpretation is consistent with previous research indicating that strongly avoidance-related experiences can have adverse effects if not accompanied by complementary acceptance-related experiences that promote psychological flexibility (Wolff et al., 2022).

##### Non-therapeutic experiences

Non-therapeutic experiences were characterized by relatively low levels of all five GCMs. Compared to other experiences, non-therapeutic experiences occurred less often in therapeutic or ceremonial settings and were associated with particularly low levels of therapeutic intention, acceptance, and introspection. Taken together, these results may suggest that non-therapeutic experiences tend to occur when psychedelics are used without any particular interest in self-inquiry or self-transformation. In such a psychological context, it can be expected that positive feelings and changes in perspective are more readily attributed to a “drug experience” than to qualities of one’s self. Consistent with this interpretation, these experiences were in many cases (63.7%) described as “rather pleasant” and were often (79.4%) deemed “positive” or “very positive” despite low levels of resource activation. Remarkably, participants assigned to this class showed low levels of current well-being despite reporting particularly low numbers of stressful life events. This result is consistent with the view that, compared to therapeutic and resource-focused experiences, non-therapeutic experiences exert fewer positive effects on well-being. Another possible explanation for the combination of relatively poor well-being with low numbers of reported stressful life events in this group of participants is the potential presence of causative factors that were not measured here, such as alexithymia, low trait introspection, or cognitive impairments that affect autobiographical memory.

#### Differences between English- and German-speaking participants

The English sample scored significantly higher on all five GCMQ scales than the German sample. Differences were also observed regarding the identified subtypes of GCM-related experiences: The English sample reported more highly therapeutic experiences, whereas the German sample reported more resource-focused experiences. These differences likely reflect the influence of several context factors that can be classified according to the contextual-experiential model (Figure 1). Relevant immediate context factors include a higher prevalence of therapeutic settings and a lower prevalence of party, context, or festival settings in the English sample. Consistent with this, the English sample exhibited more therapeutic use motives and less hedonic motives (but also more escapist motives). Relevant superordinate context factors include the higher prevalence of mental disorders and the higher number of stressful life events in the English sample, which might, to some degree, explain the mentioned differences in settings and use motives.

### GCM-related experiences moderate the association between stressful life events and mental well-being

To investigate potential longer-term psychological changes occasioned by GCM-related psychedelic experiences as implied by the contextual-experiential model (Figure 1), we examined associations between GCMs, stressful life events cumulated over the past 5 years, and mental well-being at the time of data collection. Unsurprisingly, stressful life events were negatively associated with well-being. As hypothesized, this association was significantly attenuated among participants whose reported psychedelic experiences were characterized by high levels of resource activation, clarification, or mastery. Also as hypothesized, this moderating effect was not found for problem actuation. Given the cross-sectional, retrospective design of the present study and considering that causal relationships between stressful events and well-being are complex (Cohen et al., 2019), these results must be interpreted with caution. One possible interpretation is that GCM-related psychedelic experiences counteract the adverse effects of stressful events on well-being in a protective or curative manner. Another compatible possibility, and equally consistent with the contextual-experiential model (Figure 1), is that a substantial portion of participants who were exposed to many stressful life events did not suffer lasting adverse effects on their well-being due to protective or curative superordinate context factors (e.g., resilience or psychotherapy) that led to more strongly GCM-related experiences when these participants used a psychedelic (e.g., via therapeutic intentions). These broad possibilities could be explored using the GCMQ in future longitudinal studies under controlled experimental conditions. That no moderating effect of problem actuation was found is consistent with prior findings from psychotherapy research and can be interpreted in the sense that whether problem-actuating experiences have therapeutic effects depends especially on the synergistic action of other GCMs (e.g., Gassmann and Grawe, 2006; Mander et al., 2013).

Contrary to our hypothesis, psychedelic experiences related to the GCM therapeutic relationship did not significantly moderate the association between stressful life events and mental well-being. Here, it must be mentioned that the relationship factor measured by the nonclinical version of the Relationship scale in the present nonclinical survey study should not be interpreted as entirely equivalent to the GCM therapeutic relationship in formal psychotherapeutic treatments. Responding to the Relationship items, some participants of the present survey (e.g., reporting on an experience undergone in the context of a clinical trial) may have referred to a relationship with an actual psychotherapist. However, many likely referred to some other type of role, such as a shaman, retreat facilitator, or “trip-sitter.” Characteristics of such helpers as preferred by people who use psychedelics in nonclinical settings overlap with the desirable characteristics of psychedelic therapists described in the clinical literature (Thal et al., 2022a). However, relationships with them should be assumed to differ from patient–therapist relationships in formal psychotherapy. Furthermore, it should be noted that the Relationship scale focuses on the “positive emotional bond” aspect of the therapeutic alliance while omitting the other aspects of “agreement on goals” and “agreement on tasks” (Bordin, 1979; Horvath and Luborsky, 1993).

### Limitations and future directions

This study has several limitations. Since data collection was conducted through an anonymous online survey, participants’ responses cannot be verified, and duplicate participants cannot be ruled out. Furthermore, the sample is likely subject to various selection effects, including variations in accessibility and digital competencies. A potential selection effect is that the sample was overwhelmingly composed of highly educated individuals. Likewise, younger and less educated individuals were more likely to drop out of the survey. Further studies with more representative samples are needed to test whether the GCMQ can be applied to less educated populations. Although the GCMQ was developed for both nonclinical and clinical psychedelic research, the present study did not include clinical samples. This limitation applies in particular to the Relationship scale: The clinical version of this scale was not used here and thus remains to be validated in future clinical studies. Another limitation is the retrospective, cross-sectional study design, which is susceptible to recall biases and does not allow for causal inferences. To further investigate the assumed role of GCM-related experiences as mediators of psychedelic-occasioned psychological change, prospective and longitudinal studies are required (Kangaslampi, 2020).

The present survey focused on GCM-related experiences induced by the most commonly used longer-acting classical psychedelics. Shorter-acting classical psychedelics such as inhaled DMT and atypical psychedelics such as MDMA (see Passie and Dürst, 2009) or ketamine may also induce GCM-related experiences. The exclusion of these substances limits the generalizability of the findings to a specific subset of classical psychedelics. Further research is needed to comprehensively understand the range of GCM-related experiences induced by a broader spectrum of psychedelic compounds. However, it should be noted that survey studies of naturalistic use may not allow for disentangling psychopharmacological differences between substances from the effects of the specific contexts in which different substances are used.

#### Future applications of the GCMQ in psychedelic research

In the future, the GCMQ may be applied in various areas of psychedelic and psychotherapy research. First, studies of naturalistic psychedelic use may include the GCMQ to further investigate the conditions under which GCM-related psychedelic experiences tend to occur. Moreover, naturalistic studies could help identify context factors that promote or impede successful integration and beneficial longer-term outcomes of GCM-related experiences. The present work shows that naturalistic observations of GCM-related experiences can be interpreted based on existing frameworks established by empirical psychotherapy research. Hence, results can be used to inform clinical research as well as clinical practice in psychedelic therapy and harm reduction (e.g., see our discussion of use motives and setting factors above).

Second, experimental studies, including clinical studies and studies with healthy volunteers, may apply the GCMQ directly after psychedelic dosing sessions. This will allow researchers to investigate associations between GCM-related psychedelic experiences and observed or experimentally manipulated context factors. As mentioned, such research can be informed by observational studies of naturalistic psychedelic use and guided by empirical psychotherapy research. Following our contextual-experiential model (Figure 1), relevant variables to investigate may include both immediate context factors (e.g., use motives/intentions, setting factors) and superordinate context factors, such as more enduring patient features (e.g., related to specific personality traits, psychopathological patterns, or personal abilities) or the type of psychotherapeutic framework in which psychedelic dosing sessions take place.

An essential domain of superordinate context factors to be explored in future clinical studies is the ongoing psychotherapy process itself. In a clinical trial testing psilocybin for depression, Murphy et al. (2022) found that the therapeutic relationship established before dosing sessions predicted therapeutically effective psilocybin experiences. These experiences, in turn, predicted the therapeutic relationship later in the therapy process. These results emphasize the importance of understanding psychedelic therapy as one continuous therapy process that unfolds across preparatory, dosing, and integration sessions. Consistent with this view, and in line with the contextual-experiential model (Figure 1), is the general hypothesis that a psychotherapeutic framework that successfully implements GCMs is conducive to the occurrence and effective integration of GCM-related psychedelic experiences. Conversely, it can be hypothesized that GCM-related psychedelic experiences facilitate the successful implementation of GCMs in subsequent (non-psychedelic) therapy sessions. Using the GCMQ, future clinical studies may test these hypotheses and investigate related research questions both observationally and experimentally. For instance, can psychedelic experiences associated with specific GCMs (e.g., clarification experiences) be purposefully promoted by emphasizing these GCMs in preparatory sessions (e.g., using clarification-oriented interventions)? The GCMQ enables investigation of such questions with a unique feature: Unlike other self-report instruments that are commonly used to characterize patients’ psychedelic experiences, the GCMQ has been designed to be applied not only to psychedelic dosing sessions but also to non-psychedelic therapy sessions. Thus, the same psychological constructs can describe psychotherapeutically relevant experiences in normal and altered waking consciousness.

The GCMQ was designed to measure the same or similar constructs as other instruments used in psychotherapy research, such as the SACiP or the BPSR. Convergent validity with these instruments remains to be examined in future clinical studies. As explained, the applicability of the SACiP and the BPSR to psychedelic dosing sessions is limited. Therefore, convergence between these measures and the GCMQ can only be tested in the context of conventional (non-psychedelic) talk therapy sessions, including preparatory and integration-focused sessions in psychedelic therapy.

## Conclusion

The GCMQ is a new theory-based psychometric instrument that was simultaneously developed in English and German language to assess psychotherapeutic or psychotherapy-like processes in the context of psychedelic experiences. Following the present initial validation study in a nonclinical sample, the GCMQ remains to be validated in future clinical studies.

The present study confirms that the psychological change processes that underlie the efficacy of psychotherapy are not confined to formal clinical treatments. Experiences that promote adaptive psychological change are a natural aspect of healthy human life (Bridges and Bridges, 2019; Lazarus and Folkman, 1984). Consistent with previous studies (e.g., Haijen et al., 2018; Nayak et al., 2023), our results provide evidence that naturalistic psychedelic use can, under favorable conditions, contribute to such experiences. Psychedelic therapy can be understood as the systematic effort to use a formal treatment framework to establish an optimal context for therapeutic experiences with psychedelics. A related current debate with important regulatory implications revolves around the question of what psychedelic therapy is: A medical treatment delivered with “psychological support” (Goodwin et al., 2023)? Or a psychotherapy augmented with pharmacological methods (Gründer et al., 2023)? The present results support the view that psychotherapeutic or psychotherapy-like experiences play an essential role in psychedelic-occasioned psychological change. At least from a mechanistic perspective, any mental health treatment that works by inducing such experiences can be considered a form of psychotherapy—irrespective of the specific methods involved. Future clinical studies may use the GCMQ to more directly explore the role of GCMs in mediating the efficacy of psychedelic therapy. Such efforts do not challenge the therapeutic significance of mystical-type experiences, emotional breakthrough experiences, and other experiences that may seem to distinguish psychedelic therapy from other forms of psychotherapy. Instead, as we have shown here, examining these seemingly exceptional experiences through the lens of GCMs can help clarify how they contribute to beneficial psychological change.

As psychedelic research advances, it is crucial to critically examine the pitfall of *psychedelic exceptionalism*—including the idea that psychedelic experiences are somehow too special to be accommodated by the same theoretical frameworks that can be used to understand other psychological phenomena (Johnson, 2021). Current debates about the role of psychotherapy in psychedelic treatments have, in part, been shaped by such ideas. The GCMQ is intended to make relevant concepts established by empirical psychotherapy research more accessible to psychedelic research. We hope the GCMQ can thereby contribute to the transdisciplinary exchange that is needed for the further development of an evidence-based, safe, and effective psychedelic-augmented psychotherapy.

## Supplemental Material

sj-pdf-1-jop-10.1177_02698811241249698 – Supplemental material for Measuring psychotherapeutic processes in the context of psychedelic experiences: Validation of the General Change Mechanisms Questionnaire (GCMQ) Supplemental material, sj-pdf-1-jop-10.1177_02698811241249698 for Measuring psychotherapeutic processes in the context of psychedelic experiences: Validation of the General Change Mechanisms Questionnaire (GCMQ) by Max Wolff, Ricarda Evens, Lea J Mertens, Christopher Schmidt, Jessica Beck, Hans Rutrecht, Aaron D Cherniak, Gerhard Gründer and Henrik Jungaberle in Journal of Psychopharmacology

sj-pdf-2-jop-10.1177_02698811241249698 – Supplemental material for Measuring psychotherapeutic processes in the context of psychedelic experiences: Validation of the General Change Mechanisms Questionnaire (GCMQ) Supplemental material, sj-pdf-2-jop-10.1177_02698811241249698 for Measuring psychotherapeutic processes in the context of psychedelic experiences: Validation of the General Change Mechanisms Questionnaire (GCMQ) by Max Wolff, Ricarda Evens, Lea J Mertens, Christopher Schmidt, Jessica Beck, Hans Rutrecht, Aaron D Cherniak, Gerhard Gründer and Henrik Jungaberle in Journal of Psychopharmacology

## References

[bibr1-02698811241249698] Abramson HA (1967) The Use of LSD in Psychotherapy and Alcoholism. Indianapolis, IN: Bobbs-Merrill.

[bibr2-02698811241249698] Aday J Horton D Fernandes-Osterhold G , et al. (2023) Psychedelic-assisted psychotherapy: Where is the psychotherapy research? [Preprint]. PsyArXiv. DOI: 10.31234/osf.io/s3yjd. 38782821

[bibr3-02698811241249698] Aday JS Davis AK Mitzkovitz CM , et al. (2021) Predicting reactions to psychedelic drugs: A systematic review of states and traits related to acute drug effects. ACS Pharmacol Transl Sci 4: 424–435. 33860172 10.1021/acsptsci.1c00014 PMC8033773

[bibr4-02698811241249698] Aday JS Mitzkovitz CM Bloesch EK , et al. (2020) Long-term effects of psychedelic drugs: A systematic review. Neurosci Biobehav Rev 113: 179–189. 32194129 10.1016/j.neubiorev.2020.03.017

[bibr5-02698811241249698] Baier AL Kline AC Feeny NC (2020) Therapeutic alliance as a mediator of change: A systematic review and evaluation of research. Clin Psychol Rev 82: 101921. 33069096 10.1016/j.cpr.2020.101921

[bibr6-02698811241249698] Barrett FS Bradstreet MP Leoutsakos JMS , et al. (2016) The challenging experience questionnaire: Characterization of challenging experiences with psilocybin mushrooms. J Psychopharmacol 30: 1279–1295. 27856683 10.1177/0269881116678781 PMC5549781

[bibr7-02698811241249698] Barrett FS Johnson MW Griffiths RR (2015) Validation of the revised mystical experience questionnaire in experimental sessions with psilocybin. J Psychopharmacol 29: 1182–1190. 26442957 10.1177/0269881115609019 PMC5203697

[bibr8-02698811241249698] Basedow LA Kuitunen-Paul S (2022) Motives for the use of serotonergic psychedelics: A systematic review. Drug Alcohol Rev 41: 1391–1403. 35668698 10.1111/dar.13480

[bibr9-02698811241249698] Bordin ES (1979) The generalizability of the psychoanalytic concept of the working alliance. Psychother Theory Res Pract 16: 252.

[bibr10-02698811241249698] Brauns H Scherer S Steinmann S (2003) The CASMIN educational classification in international comparative research. In: Hoffmeyer-Zlotnik JHP Wolf C (eds.) Advances in Cross-National Comparison. Berlin, Germany: Springer Science & Business Media, pp. 221–244.

[bibr11-02698811241249698] Bridges W Bridges S (2019) Transitions: Making Sense of Life’s Changes. London, UK: Hachette UK.

[bibr12-02698811241249698] Brown TA (2015) Confirmatory Factor Analysis for Applied Research. New York, NY: Guilford Publications.

[bibr13-02698811241249698] Carbonaro TM Bradstreet MP Barrett FS , et al. (2016) Survey study of challenging experiences after ingesting psilocybin mushrooms: Acute and enduring positive and negative consequences. J Psychopharmacol 30: 1268–1278. 27578767 10.1177/0269881116662634 PMC5551678

[bibr14-02698811241249698] Carhart-Harris RL Nutt DJ (2017) Serotonin and brain function: A tale of two receptors. J Psychopharmacol 31: 1091–1120. 28858536 10.1177/0269881117725915 PMC5606297

[bibr15-02698811241249698] Carhart-Harris RL Roseman L Haijen E , et al. (2018) Psychedelics and the essential importance of context. J Psychopharmacol 32: 725–731. 29446697 10.1177/0269881118754710

[bibr16-02698811241249698] Cohen S Murphy ML Prather AA (2019) Ten surprising facts about stressful life events and disease risk. Ann Rev Psychol 70: 577–597. 29949726 10.1146/annurev-psych-010418-102857 PMC6996482

[bibr17-02698811241249698] Cuijpers P Reijnders M Huibers MJH (2019) The role of common factors in psychotherapy outcomes. Ann Rev Clin Psychol 15: 207–231. 30550721 10.1146/annurev-clinpsy-050718-095424

[bibr18-02698811241249698] Davis AK Barrett FS Griffiths RR (2020) Psychological flexibility mediates the relations between acute psychedelic effects and subjective decreases in depression and anxiety. J Context Behav Sci 15: 39–45. 10.1016/j.jcbs.2019.11.004 PMC7451132 32864325

[bibr19-02698811241249698] Davis AK Barrett FS So S , et al. (2021) Development of the psychological insight questionnaire among a sample of people who have consumed psilocybin or LSD. J Psychopharmacol 35: 437–446. 33427007 10.1177/0269881120967878 PMC8056708

[bibr20-02698811241249698] DeFife JA Hilsenroth MJ (2011) Starting off on the right foot: Common factor elements in early psychotherapy process. J Psychother Integr 21: 172.

[bibr21-02698811241249698] Dittrich A (1998) The standardized psychometric assessment of altered states of consciousness (ASCs) in humans. Pharmacopsychiatry 31: 80–84. 9754838 10.1055/s-2007-979351

[bibr22-02698811241249698] Dworatzyk K Jansen T Schmidt TT (2021) Phenomenological assessment of psychedelic induced experiences: Translation and validation of the German Challenging Experience Questionnaire (CEQ) and Ego-Dissolution Inventory (EDI). PLoS One 17: e0264927. 10.1371/journal.pone.0264927 PMC8926265 35294453

[bibr23-02698811241249698] Elson M (2017) Question wording and item formulation. In: Matthes J Potter R Davis CS (eds.) International Encyclopedia of Communication Research Methods. Hoboken, NJ: Wiley-Blackwell, pp. 1–8.

[bibr24-02698811241249698] Evans J Robinson OC Argyri EK , et al. (2023) Extended difficulties following the use of psychedelic drugs: A mixed methods study. PLoS One 18: e0293349. 37874826 10.1371/journal.pone.0293349 PMC10597511

[bibr25-02698811241249698] Flückiger C Caspar F Holtforth MG , et al. (2009) Working with patients’ strengths: A microprocess approach. Psychother Res 19: 213–223. 19396652 10.1080/10503300902755300

[bibr26-02698811241249698] Flückiger C Del Re AC Wampold BE , et al. (2018) The alliance in adult psychotherapy: A meta-analytic synthesis. Psychotherapy 55: 316–340. 29792475 10.1037/pst0000172

[bibr27-02698811241249698] Flückiger C Regli D Zwahlen D , et al. (2010a). Der Berner Patienten-und Therapeutenstundenbogen 2000. Zeitschrift für Klinische Psychol Psychother 39: 71–79.

[bibr28-02698811241249698] Flückiger C Wüsten G Zinbarg R , et al. (2010b). Resource Activation: Using Clients’ Own Strengths in Psychotherapy and Counseling. Boston, MA: Hogrefe Publishing.

[bibr29-02698811241249698] Frank JD (1961) Persuasion and Healing: A Comparative Study of Psychotherapy. Baltimore, MD: Johns Hopkins University Press.

[bibr30-02698811241249698] Gandy S Forstmann M Carhart-Harris RL , et al. (2020) The potential synergistic effects between psychedelic administration and nature contact for the improvement of mental health. Health Psychol Open 7: 2055102920978123. 33335742 10.1177/2055102920978123 PMC7724423

[bibr31-02698811241249698] Garcia-Romeu A Richards WA (2018) Current perspectives on psychedelic therapy: Use of serotonergic hallucinogens in clinical interventions. Int Rev Psychiatry 30: 291–316. 30422079 10.1080/09540261.2018.1486289

[bibr32-02698811241249698] Garfield SL (1982) Electicism and integration in psychotherapy. Behav Ther 13: 610–623.

[bibr33-02698811241249698] Gassmann D Grawe K (2006) General change mechanisms: The relation between problem activation and resource activation in successful and unsuccessful therapeutic interactions. Clin Psychol Psychother 13: 1–11.

[bibr34-02698811241249698] Goldfried MR (1980) Toward the delineation of therapeutic change principles. Am Psychol 35: 991–999. 7436119 10.1037//0003-066x.35.11.991

[bibr35-02698811241249698] Goodwin GM Malievskaia E Fonzo GA , et al. (2023) Must psilocybin always “Assist Psychotherapy”? Am J Psychiatry 181: 20–25. 37434509 10.1176/appi.ajp.20221043

[bibr36-02698811241249698] Grawe K (1997) Research-informed psychotherapy. Psychother Res 7: 1–19.

[bibr37-02698811241249698] Grawe K (2004). Psychological Therapy. Boston, MA: Hogrefe Publishing.

[bibr38-02698811241249698] Grawe K (2007) Neuropsychotherapy: How the Neurosciences Inform Effective Psychotherapy. London, UK: Routledge. 10.1037/0033-3204.44.1.118 22122176

[bibr39-02698811241249698] Greenway KT Garel N Jerome L , et al. (2020) Integrating psychotherapy and psychopharmacology: Psychedelic-assisted psychotherapy and other combined treatments. Exp Rev Clin Pharmacol 13: 655–670. 10.1080/17512433.2020.1772054 32478631

[bibr40-02698811241249698] Griffiths RR Johnson MW Richards WA , et al. (2018) Psilocybin-occasioned mystical-type experience in combination with meditation and other spiritual practices produces enduring positive changes in psychological functioning and in trait measures of prosocial attitudes and behaviors. J Psychopharmacol 32: 49–69. 29020861 10.1177/0269881117731279 PMC5772431

[bibr41-02698811241249698] Grosse Holtforth M Flückiger C (2012) The stream of corrective experiences in action: Big bang and constant dripping. In: Castonguay LG Hill CE (eds.) Transformation in Psychotherapy: Corrective Experiences Across Cognitive Behavioral, Humanistic, and Psychodynamic Approaches. Washington, DC: American Psychological Association, pp. 317–333.

[bibr42-02698811241249698] Gründer G Brand M Mertens LJ , et al. (2023) Treatment with psychedelics is psychotherapy: Beyond reductionism. Lancet Psychiatry 11: S2215036623003632. 10.1016/S2215-0366(23)00363-2 38101439

[bibr43-02698811241249698] Haijen ECHM Kaelen M Roseman L , et al. (2018) Predicting responses to psychedelics: A prospective study. Front Pharmacol 9: 1–20. 30450045 10.3389/fphar.2018.00897 PMC6225734

[bibr44-02698811241249698] Hartogsohn I (2017) Constructing drug effects: A history of set and setting. Drug Sci Policy Law 3: 205032451668332.

[bibr45-02698811241249698] Hayes SC Law S Malady M , et al. (2020) The centrality of sense of self in psychological flexibility processes: What the neurobiological and psychological correlates of psychedelics suggest. J Context Behav Sci 15: 30–38.

[bibr46-02698811241249698] Healy CJ Lee KA D’Andrea W (2021) Using psychedelics with therapeutic intent is associated with lower shame and complex trauma symptoms in adults with histories of child maltreatment. Chronic Stress 5: 1–12. 10.1177/24705470211029881 PMC8278461 34291179

[bibr47-02698811241249698] Horvath AO Luborsky L (1993) The role of the therapeutic alliance in psychotherapy. J Consult Clin Psychol 61: 561. 8370852 10.1037//0022-006x.61.4.561

[bibr48-02698811241249698] Johnson MW (2021) Consciousness, religion, and gurus: Pitfalls of psychedelic medicine. ACS Pharmacol Transl Sci 4: 578–581. 33860187 10.1021/acsptsci.0c00198 PMC8033601

[bibr49-02698811241249698] Johnson MW Hendricks PS Barrett FS , et al. (2019) Classic psychedelics: An integrative review of epidemiology, therapeutics, mystical experience, and brain network function. Pharmacol Therap 197: 83–102. 30521880 10.1016/j.pharmthera.2018.11.010

[bibr50-02698811241249698] Johnson MW Richards W Griffiths R (2008) Human hallucinogen research: Guidelines for safety. J Psychopharmacol 22: 603–620. 18593734 10.1177/0269881108093587 PMC3056407

[bibr51-02698811241249698] Jungaberle H Gasser P Weinhold J , et al. (2008). Therapie mit Psychoaktiven Substanzen. Berner Verlag: Verlag Hans Huber.

[bibr52-02698811241249698] Kangaslampi S (2020) Uncovering psychological mechanisms mediating the effects of drugs: Some issues and comments using the example of psychedelic drugs. Psychopharmacology 237: 3799–3802. 33151375 10.1007/s00213-020-05703-9 PMC7683478

[bibr53-02698811241249698] Kettner H Rosas FE Timmermann C , et al. (2021) Psychedelic communitas: Intersubjective Experience During Psychedelic Group sessions predicts enduring changes in psychological wellbeing and social connectedness. Front Pharmacol 12: 1–20. 10.3389/fphar.2021.623985 PMC8114773 33995022

[bibr54-02698811241249698] Lazarus RS Folkman S (1984) Stress, Appraisal, and Coping. Berlin, Germany: Springer Publishing Company.

[bibr55-02698811241249698] Leiner DJ (2019a). SoSci Survey (Version 3.1.06) [Computer Software]. https://www.soscisurvey.de

[bibr56-02698811241249698] Leiner DJ (2019b) Too fast, too straight, too weird: Non-reactive indicators for meaningless data in internet surveys. Survey Res Methods 13: 229–248.

[bibr57-02698811241249698] Lubke GH Muthén B (2005) Investigating population heterogeneity with factor mixture models. Psychol Methods 10: 21. 15810867 10.1037/1082-989X.10.1.21

[bibr58-02698811241249698] Luoma JB Sabucedo P Eriksson J , et al. (2019) Toward a contextual psychedelic-assisted therapy: Perspectives from acceptance and commitment therapy and contextual behavioral science. J Context Behav Sci 14: 136–145.

[bibr59-02698811241249698] Madsen MK Fisher PMD Stenbæk DS , et al. (2020). A single psilocybin dose is associated with long-term increased mindfulness, preceded by a proportional change in neocortical 5-HT2A receptor binding. Eur Neuropsychopharmacol 33: 71–80. 32146028 10.1016/j.euroneuro.2020.02.001

[bibr60-02698811241249698] Majić T Schmidt TT Gallinat J (2015) Peak experiences and the afterglow phenomenon: When and how do therapeutic effects of hallucinogens depend on psychedelic experiences? J Psychopharmacol 29: 241–253. 25670401 10.1177/0269881114568040

[bibr61-02698811241249698] Mander JV Wittorf A Schlarb A , et al. (2013) Change mechanisms in psychotherapy: Multiperspective assessment and relation to outcome. Psychother Res 23:105–116. 10.1080/10503307.2012.744111 23194587

[bibr62-02698811241249698] McCulloch DE-W Grzywacz MZ Madsen MK , et al. (2022). Psilocybin-induced mystical-type experiences are related to persisting positive effects: A quantitative and qualitative report. Front Pharmacol 13: 1–17. 10.3389/fphar.2022.841648 PMC8959755 35355714

[bibr63-02698811241249698] McGuire-Snieckus R McCabe R Catty J , et al. (2007) A new scale to assess the therapeutic relationship in community mental health care: STAR. Psychol Med 37: 85–95. 17094819 10.1017/S0033291706009299

[bibr64-02698811241249698] Michalak J Holtforth MG (2006) Where do we go from here? The goal perspective in psychotherapy. Clin Psychol Sci Pract 13: 346–365.

[bibr65-02698811241249698] Mitchell JM Anderson BT (2023) Psychedelic therapies reconsidered: Compounds, clinical indications, and cautious optimism. Neuropsychopharmacology 49: 96–103. 37479859 10.1038/s41386-023-01656-7 PMC10700471

[bibr66-02698811241249698] Moggia D Bennemann B Schwartz B , et al. (2023) Process-based psychotherapy personalization: Considering causality with continuous-time dynamic modeling. Psychother Res 33: 1076–1095. 37306112 10.1080/10503307.2023.2222892

[bibr67-02698811241249698] Müller F Johnson MW Borgwardt S (2020). Editorial: Hallucinogens and entactogens: Establishing a new class of psychotherapeutic drugs? Front Psychiatry 11: 497. 32547435 10.3389/fpsyt.2020.00497 PMC7270170

[bibr68-02698811241249698] Murphy R Kettner H Zeifman R , et al. (2022) Therapeutic alliance and rapport modulate responses to psilocybin assisted therapy for depression. Front Pharmacol 12: 1–19. 10.3389/fphar.2021.788155 PMC9009076 35431912

[bibr69-02698811241249698] Nayak S Johnson MW (2020) Psychedelics and psychotherapy. Pharmacopsychiatry 54: 167–175. 33285578 10.1055/a-1312-7297

[bibr70-02698811241249698] Nayak SM Jackson H Sepeda ND , et al. (2023) Naturalistic psilocybin use is associated with persisting improvements in mental health and wellbeing: Results from a prospective, longitudinal survey. Front Psychiatry 14: 1199642. 37795509 10.3389/fpsyt.2023.1199642 PMC10545967

[bibr71-02698811241249698] Nichols DE Walter H (2021) The history of psychedelics in psychiatry. Pharmacopsychiatry 54: 151–166. 33285579 10.1055/a-1310-3990

[bibr72-02698811241249698] Norcross JC Lambert MJ (2018) Psychotherapy relationships that work III. Psychotherapy 55: 303–315. 30335448 10.1037/pst0000193

[bibr73-02698811241249698] Orlinsky DE Grawe K Parks BK (1994) Process and outcome in psychotherapy: Noch einmal. In: Bergin AE Garfield SL (eds.) Handbook of Psychotherapy and Behavior Change, 4th ed. New York, NY: John Wiley & Sons, pp. 270–376.

[bibr74-02698811241249698] Orlinsky DE Howard KI (1987) A generic model of psychotherapy. J Integr Eclect Psychother 6: 6–27.

[bibr75-02698811241249698] Passie T Dürst T (2009) Heilungsprozesse im Veränderten Bewusstsein [Healing Processes in Altered Consciousness]. Berlin: VWB - Verlag für Wissenschaft und Bildung.

[bibr76-02698811241249698] Peill JM Trinci KE Kettner H , et al. (2022) Validation of the psychological insight scale: A new scale to assess psychological insight following a psychedelic experience. J Psychopharmacol 36: 31–45. 34983255 10.1177/02698811211066709 PMC8801624

[bibr77-02698811241249698] Perkins D Schubert V Simonová H , et al. (2021) Influence of context and setting on the mental health and wellbeing outcomes of Ayahuasca drinkers: Results of a large international survey. Front Pharmacol 12: 623979. 33967757 10.3389/fphar.2021.623979 PMC8097729

[bibr78-02698811241249698] Pilecki B Luoma JB Bathje GJ , et al. (2021) Ethical and legal issues in psychedelic harm reduction and integration therapy. Harm Reduct J 18: 40. 33827588 10.1186/s12954-021-00489-1 PMC8028769

[bibr79-02698811241249698] Renelli M Fletcher J Tupper KW , et al. (2020) An exploratory study of experiences with conventional eating disorder treatment and ceremonial ayahuasca for the healing of eating disorders. Eating Weight Disord 25: 437–444. 10.1007/s40519-018-0619-6 30474794

[bibr80-02698811241249698] Roberts CA Osborne-Miller I Cole J , et al. (2020) Perceived harm, motivations for use and subjective experiences of recreational psychedelic ‘magic’ mushroom use. J Psychopharmacol 34: 999–1007. 32674668 10.1177/0269881120936508

[bibr81-02698811241249698] Roseman L Haijen E Idialu-Ikato K , et al. (2019). Emotional breakthrough and psychedelics: Validation of the emotional breakthrough inventory. J Psychopharmacol 33: 1076–1087. 31294673 10.1177/0269881119855974

[bibr82-02698811241249698] Rosenzweig S (1936) Some implicit common factors in diverse methods of psychotherapy. Am J Orthopsychiatr 6: 412.

[bibr83-02698811241249698] Rubel JA Rosenbaum D Lutz W (2017) Patients’ in-session experiences and symptom change: Session-to-session effects on a within- and between-patient level. Behavi Res Ther 90: 58–66. 10.1016/j.brat.2016.12.007 27998800

[bibr84-02698811241249698] Sandison RA (1954) Psychological aspects of the LSD treatment of the neuroses. J Mental Sci 100: 508–515. 10.1192/bjp.100.419.508 13175012

[bibr85-02698811241249698] Satorra A Bentler PM (2001) A scaled difference chi-square test statistic for moment structure analysis. Psychometrika 66: 507–514. 10.1007/s11336-009-9135-y PMC2905175 20640194

[bibr86-02698811241249698] Schlichting M (2000) Wirkfaktoren der psycholytischen therapie. In Schlichting M (Hrsg.), Welten des Bewusstseins. Berlin: VWB - Verlag für Wissenschaft und Bildung, pp. 67–76.

[bibr87-02698811241249698] Seery MD Holman EA Silver RC (2010) Whatever does not kill us: Cumulative lifetime adversity, vulnerability, and resilience. J Personal Soc Psychol 99: 1025–1041. 10.1037/a0021344 20939649

[bibr88-02698811241249698] Smigielski L Kometer M Scheidegger M , et al. (2019) Characterization and prediction of acute and sustained response to psychedelic psilocybin in a mindfulness group retreat. Scient Rep 9: 1–13. 10.1038/s41598-019-50612-3 PMC6813317 31649304

[bibr89-02698811241249698] Straumann I Ley L Holze F , et al. (2023) Acute effects of MDMA and LSD co-administration in a double-blind placebo-controlled study in healthy participants. Neuropsychopharmacology 48: 1840–1848. 37258715 10.1038/s41386-023-01609-0 PMC10584820

[bibr90-02698811241249698] Studerus E Gamma A Vollenweider FX (2010) Psychometric evaluation of the altered states of consciousness rating scale (OAV). PLoS One 5: e12412. 20824211 10.1371/journal.pone.0012412 PMC2930851

[bibr91-02698811241249698] Tanzer NK (2005) Developing tests for use in multiple languages and cultures: A plea for simultaneous development. In: Hambleton RK Merenda PF Spielberger CD (Hrsg.) Adapting Educational and Psychological Tests for Cross-Cultural Assessment. Erlbaum, NJ: Erlbaum, pp. 235–263.

[bibr92-02698811241249698] Tennant R Hiller L Fishwick R , et al. (2007). The Warwick-Edinburgh mental well-being scale (WEMWBS): Development and UK validation. Health Qual Life Outcomes 5: 1–13. 18042300 10.1186/1477-7525-5-63 PMC2222612

[bibr93-02698811241249698] Thal SB Engel LB Bright SJ (2022a) Presence, trust, and empathy: Preferred characteristics of psychedelic carers. J Human Psychol 89: 1–24. DOI: 10.1177/00221678221081380.

[bibr94-02698811241249698] Thal SB Wieberneit M Sharbanee JM , et al. (2022b) Therapeutic (Sub) stance: Current practice and therapeutic conduct in preparatory sessions in substance-assisted psychotherapy—A systematized review. J Psychopharmacol 36: 1191–1207. 36263882 10.1177/02698811221127954

[bibr95-02698811241249698] Timmermann C Zeifman RJ Erritzoe D , et al. (2024) Effects of DMT on mental health outcomes in healthy volunteers. Scient Rep 14: 3097. 10.1038/s41598-024-53363-y PMC10850177 38326357

[bibr96-02698811241249698] Wampold BE Imel ZE (2015) The Great Psychotherapy Debate: The Evidence for What Makes Psychotherapy Work. London, UK: Routledge.

[bibr97-02698811241249698] Watts R Day C Krzanowski J , et al. (2017) Patients’ accounts of increased “connectedness” and “acceptance” after psilocybin for treatment-resistant depression. J Human Psychol 57: 520–564.

[bibr98-02698811241249698] Wolff M Dziobek I Gründer G , et al. (under review) Psychedelic therapy is psychotherapy: Understanding psychedelic-occasioned psychological change through the lens of Grawe’s general change mechanisms. 10.1037/rev0000589 41066253

[bibr99-02698811241249698] Wolff M Evens R Mertens LJ , et al. (2020). Learning to let go: A cognitive-behavioral model of how psychedelic therapy promotes acceptance. Front Psychiatry 11: 5. 32153433 10.3389/fpsyt.2020.00005 PMC7046795

[bibr100-02698811241249698] Wolff M Mertens LJ Walter M , et al. (2022). The Acceptance/Avoidance-Promoting Experiences Questionnaire (APEQ): A theory-based approach to psychedelic drugs’ effects on psychological flexibility. J Psychopharmacol 36: 387–408. 35253518 10.1177/02698811211073758 PMC8902683

[bibr101-02698811241249698] Wollburg E Braukhaus C (2010) Goal setting in psychotherapy: The relevance of approach and avoidance goals for treatment outcome. Psychother Res 20: 488–494. 20665341 10.1080/10503301003796839

[bibr102-02698811241249698] Wrede N Töpfer NF Wilz G (2023) Between- and within-person effects of affective experiences on coping in CBT: Direct effects and interplay with therapeutic alliance and resource activation. Psychother Res 1–15. 10.1080/10503307.2023.2277290 37922397

[bibr103-02698811241249698] Yaden DB Griffiths RR (2021) The subjective effects of psychedelics are necessary for their enduring therapeutic effects. ACS Pharmacol Transl Sci 4: 568–572. 33861219 10.1021/acsptsci.0c00194 PMC8033615

[bibr104-02698811241249698] Zeifman RJ Kettner H Pagni BA , et al. (2023) Co-use of MDMA with psilocybin/LSD may buffer against challenging experiences and enhance positive experiences. Scient Rep 13: 13645. 10.1038/s41598-023-40856-5 PMC10444769 37608057

