# Supplementary material for: Measuring psychotherapeutic processes in the context of psychedelic experiences: Validation of the General Change Mechanisms Questionnaire (GCMQ)
Source: J Psychopharmacol. 2024 May 14;38(5):432–57. doi: 10.1177/02698811241249698 (PMC11102652; doi:10.1177/02698811241249698)
Supplement: sj-pdf-1-jop-10.1177_02698811241249698 – Supplemental material for Measuring psychotherapeutic processes in the context of psychedelic experiences: Validation of the General Change Mechanisms Questionnaire (GCMQ) [file sj-pdf-1-jop-10.1177_02698811241249698.pdf]

Supplemental Information

Measuring Psychotherapeutic Processes in the Context of Psychedelic Experiences: Validation of the General Change Mechanisms Questionnaire (GCMQ)

Table S1. *Stressful Life Events Counted in the Complete Bilingual Sample (N=1,867).*

Item #	Item text	Mean (SD) # of occurrences counted per participant
1.	I suffered a serious accident or serious injury.	0.38 (0.75)
2.	A person close to me suffered a serious accident or injury.	0.61 (0.99)
3.	I suffered a serious illness.	0.43 (0.86)
4.	A person close to me suffered a serious illness.	0.98 (1.15)
5.	I witnessed a family member being seriously injured or killed.	0.11 (0.43)
6.	I witnessed someone being seriously injured or killed.	0.22 (0.70)
7.	I was coerced or blackmailed with threats of harm to myself or my family.	0.15 (0.58)
8.	I experienced a forced separation from family members.	0.26 (0.75)
9.	I experienced a forced separation from another person close to me.	0.43 (0.90)
10.	My partner died.	0.01 (0.14)
11.	My mother or father died.	0.11 (0.33)
12.	My brother or sister died.	0.02 (0.17)
13.	My grandmother or grandfather died.	0.50 (0.79)
14.	My child died.	0.01 (0.11)
15.	Another person that I was close to died.	0.49 (0.90)
16.	Someone close to me committed suicide.	0.18 (0.52)
17.	My partner broke up with me.	0.57 (0.85)
18.	My marriage got divorced.	0.06 (0.26)
19.	My parents separated from each other.	0.05 (0.24)
20.	I lost my job.	0.48 (0.86)
21.	I experienced very serious financial difficulties (e.g., no money for food or shelter).	0.53 (1.11)
22.	I experienced a major fire, flood, earthquake, or any natural disaster in my community.	0.18 (0.55)
23.	I experienced a tragedy or disaster in my community that was intentionally caused by people (a shooting, bombing, etc.).	0.16 (0.61)
24.	I was exposed to dangerous chemicals or biological agents.	0.14 (0.64)
25.	I had combat experience within an armed conflict.	0.03 (0.27)
26.	I lived in a dangerous neighborhood.	0.22 (0.68)
27.	I was mugged or robbed.	0.10 (0.39)
28.	I was physically attacked or assaulted.	0.25 (0.70)
29.	I was discriminated against because of my ethnicity, religious background, or sexual orientation.	0.46 (1.11)
30.	I was sexually abused or raped.	0.15 (0.57)
31.	Someone close to me was sexually abused or raped.	0.22 (0.68)
32.	I was hit or pushed by my partner.	0.22 (0.73)
33.	I was unwantedly in a forced sexual relationship.	0.09 (0.45)
34.	I or my partner was unwantedly pregnant.	0.07 (0.31)
35.	I or my partner had an abortion.	0.07 (0.29)
36.	I or my partner had a miscarriage.	0.05 (0.30)

Note: Up to four occurrences over the past five years were counted per participant.
SD = standard deviation.

Table S2. *Candidate Items for the Resource Activation (RA) Scale, Descriptive Statistics, and Factor Loadings for Confirmatory Factor Analyses (CFAs) Calculated for Item Selection.*

Item	Item text		Mean (SD)		Skewness		Kurtosis		Factor loadings	
	English	German	English	German	English	German	English	German	Before selection	After selection
RA01	**I could see myself in a positive light.**	**Ich konnte mich selbst in einem positiven Licht sehen.**	3.21 (1.53)	2.72 (1.52)	-.591	-.304	-.643	-.856	.777	.745
RA02	I felt connected to important people in my life.	Ich habe mich wichtigen Menschen in meinem Leben verbunden gefühlt.	3.12 (1.70)	3.02 (1.63)	-.515	-.531	-1.011	-.889	.717	—
RA03	I had positive feelings that I hadn't felt in a while, or ever before.	Ich hatte positive Gefühle, die ich schon lange nicht mehr oder noch nie zuvor hatte.	3.17 (1.66)	2.64 (1.73)	-.509	-.133	-.944	-1.283	.624	—
RA04	I could see positive aspects of things I have been struggling with in the past.	Ich konnte positive Aspekte an Dingen sehen, mit denen ich in der Vergangenheit zu kämpfen hatte.	2.69 (1.72)	2.09 (1.66)	-.158	.229	-1.244	-1.189	.689	—
RA05	I noticed things that I like about myself.	Ich habe Dinge bemerkt, die ich an mir mag.	2.43 (1.58)	2.09 (1.52)	.072	.194	-1.084	-.992	.759	—
RA06	**I became aware of things that I am thankful for.**	**Mir wurden Dinge bewusst, für die ich dankbar bin.**	3.30 (1.65)	3.33 (1.61)	-.657	-.746	-.768	-.514	.829	.837
RA07	I realized what is really important to me in life.	Mir wurde klar, was mir wirklich wichtig ist im Leben.	2.98 (1.72)	2.59 (1.66)	-.312	-.134	-1.197	-1.162	.758	—
RA08	**I felt connected to things that give my life meaning.**	**Ich habe mich Dingen verbunden gefühlt, die meinem Leben Sinn geben.**	3.30 (1.60)	2.88 (1.62)	-.590	-.423	-.793	-.944	.826	.835
RA09	**I felt in contact with my strengths.**	**Ich fühlte mich im Kontakt mit meinen Stärken.**	2.61 (1.63)	2.35 (1.53)	-.075	-.078	-1.114	-.998	.793	.759
RA10	**I became aware of the beautiful things in my life.**	**Ich bin mir der schönen Dinge in meinem Leben bewusst geworden.**	3.42 (1.59)	3.04 (1.60)	-.691	-.489	-.656	-.839	.827	.863
RA11	I was able to forgive myself.	Ich konnte mir selbst vergeben.	2.48 (1.70)	2.09 (1.59)	.006	.157	-1.220	-1.071	.710	—
RA12	My existence felt meaningful.	Meine Existenz hat sich sinnvoll angefühlt.	3.41 (1.69)	3.05 (1.69)	-.745	-.545	-.711	-.908	.755	—

Note. Descriptive statistics are reported for the complete English sample (n=1,153) and the German sample (n=714). Confirmatory factor analyses (CFAs) for item selection were calculated in the selection stratum (n=577) of the English Sample. The five items that were selected for the final GCMQ are written in bold font.

Table S3. *Candidate Items for the Problem Actuation (PA) Scale, Descriptive Statistics, and Factor Loadings for Confirmatory Factor Analyses (CFAs) Calculated for Item Selection.*

	Item text		Mean (SD)		Skewness		Kurtosis		Factor loadings	
Item	English	German	English	German	English	German	English	German	Before selection	After selection
PA01	**I relived painful or fearful memories.**	**Ich habe schmerzhafte oder angstvolle Erinnerungen wiedererlebt.**	1.64 (1.87)	1.22 (1.60)	.733	1.179	-.889	.116	.690	.686
PA02	My typical problematic responses to certain things were activated.	Meine typischen problematischen Reaktionen auf bestimmte Dinge wurden aktiviert.	0.99 (1.39)	1.04 (1.43)	1.471	1.279	1.303	.561	.431	—
PA03	I became painfully aware of my difficulties.	Meine Schwierigkeiten wurden mir schmerzhaft bewusst.	1.74 (1.72)	1.42 (1.69)	.609	.879	-.963	-.466	.694	—
PA04	**I experienced feelings that I previously avoided.**	**Ich habe Gefühle erlebt, die ich zuvor vermieden habe.**	2.17 (1.80)	1.82 (1.71)	.303	.505	-1.313	-1.078	.760	.769
PA05	**I was confronted with my problems.**	**Ich wurde mit meinen Problemen konfrontiert.**	2.17 (1.76)	1.96 (1.67)	.299	.441	-1.253	-1.058	.869	.859
PA06	**I could feel that my vulnerable spots were being touched upon.**	**Ich konnte spüren, dass meine verletzlichen Stellen berührt wurden.**	2.55 (1.80)	2.23 (1.74)	-.047	.162	-1.380	-1.306	.796	.816
PA07	**I felt emotions that are related to my problems.**	**Ich spürte Emotionen, die mit meinen Problemen zusammenhängen.**	2.51 (1.76)	2.09 (1.69)	.044	.260	-1.332	-1.261	.834	.835
PA08	I experienced negative emotions.	Ich habe negative Emotionen erlebt.	1.71 (1.64)	1.83 (1.66)	.753	.621	-.643	-.837	.647	—

Note. Descriptive statistics are reported for the complete English sample (n=1,153) and the German sample (n=714). Confirmatory factor analyses (CFAs) for item selection were calculated in the selection stratum (n=577) of the English Sample. The five items that were selected for the final GCMQ are written in bold font.

Table S4. *Candidate Items for the Clarification (CL) Scale, Descriptive Statistics, and Factor Loadings for Confirmatory Factor Analyses (CFAs) Calculated for Item Selection.*

Item	Item text: English	Item text: German	Mean (SD): English	Mean (SD): German	Skewness: English	Skewness: German	Kurtosis: English	Kurtosis: German	Factor loadings: Before selection	Factor loadings: After selection
CL01	**I gained a new understanding of the difficulties in my life.**	**Ich habe ein neues Verständnis für die Schwierigkeiten in meinem Leben gewonnen.**	3.07 (1.62)	2.61 (1.53)	-.432	-.163	-1.007	-1.021	.813	.820
CL02	**It became clearer to me why I tend to react in certain ways to certain difficult situations.**	**Mir ist klarer geworden, warum ich in bestimmten schwierigen Situationen dazu neige, auf bestimmte Weise zu reagieren.**	2.66 (1.70)	2.32 (1.67)	-.109	.035	-1.245	-1.251	.833	.831
CL03	I became more aware of certain fears or wishes that underlie my feelings and behavior.	Ich bin mir bestimmter Ängste oder Wünsche bewusster geworden, die meinen Gefühlen und meinem Verhalten zugrunde liegen	2.72 (1.68)	2.47 (1.61)	-.152	-.061	-1.212	-1.149	.795	—
CL04	I became more aware of the role that certain formative experiences play in my life.	Mir ist bewusster geworden, welche Rolle bestimmte prägende Erfahrungen in meinem Leben spielen.	2.68 (1.75)	2.21 (1.72)	-.136	.156	-1.280	-1.279	.758	—
CL05	I became aware of previously unconscious inner conflicts.	Mir sind zuvor unbewusste innere Konflikte bewusst geworden.	2.17 (1.73)	1.80 (1.63)	.246	.515	-1.273	-.969	.713	—
CL06	**It became clearer to me what the essence of my problems is.**	**Es wurde mir klarer, was der Kern meiner Probleme ist.**	2.24 (1.64)	1.84 (1.56)	.226	.425	-1.113	-.960	.802	.817
CL07	**I could see my problems in a new light.**	**Ich konnte meine Probleme in einem neuen Licht sehen.**	2.65 (1.69)	2.23 (1.60)	-.095	.071	-1.231	-1.183	.852	.861
CL08	I felt that I could understand myself better.	Ich hatte das Gefühl, mich selbst besser verstehen zu können.	3.23 (1.58)	2.88 (1.56)	-.507	-.385	-.888	-.893	.767	—
CL09	I became aware of certain patterns that keep showing up in my relationships with other people.	Mir sind bestimme Muster bewusst geworden, die sich in meinen Beziehungen mit anderen Personen immer wieder zeigen.	2.35 (1.76)	2.10 (1.65)	.100	.194	-1.324	-1.169	.726	—
CL10	**I could understand my feelings on a deeper level.**	**Ich konnte meine Gefühle auf einer tieferen Ebene verstehen.**	3.11 (1.64)	2.73 (1.63)	-.468	-.246	-.980	-1.100	.817	.796

Note. Descriptive statistics are reported for the complete English sample (n=1,153) and the German sample (n=714). Confirmatory factor analyses (CFAs) for item selection were calculated in the selection stratum (n=577) of the English Sample. The five items that were selected for the final GCMQ are written in bold font.

Table S5. *Candidate Items for the Mastery (MA) Scale, Descriptive Statistics, and Factor Loadings for Confirmatory Factor Analyses (CFAs) Calculated for Item Selection.*

	Item text		Mean (SD)		Skewness		Kurtosis		Factor loadings	
Item	English	German	English	German	English	German	English	German	Before selection	After selection
MA01	**I made progress in coping with my problems.**	**Ich habe Fortschritte bei der Bewältigung meiner Probleme gemacht.**	2.93 (1.67)	2.37 (1.59)	-.368	.004	-1.067	-1.093	.816	.812
MA02	**I learned things that can help me handle difficult situations.**	**Ich habe Dinge gelernt, die mir dabei helfen können, mit schwierigen Situationen umzugehen.**	2.63 (1.65)	2.33 (1.55)	-.124	.001	-1.157	-1.089	.822	.843
MA03	I felt empowered to face the difficulties in my life.	Ich habe mich befähigt gefühlt, mich den Schwierigkeiten in meinem Leben zu stellen.	2.93 (1.62)	2.46 (1.58)	-.311	-.128	-1.055	-1.063	.793	—
MA04	**I gained confidence in my ability to handle strong feelings.**	**Ich habe Vertrauen in meine Fähigkeit gewonnen, mit starken Gefühlen umzugehen.**	2.52 (1.66)	2.16 (1.58)	-.052	.111	-1.183	-1.102	.798	.770
MA05	**I found new ways to deal with situations that have caused me problems in the past.**	**Ich habe neue Wege gefunden, mit Situationen umzugehen, die mir in der Vergangenheit Probleme gemacht haben.**	2.10 (1.67)	1.75 (1.53)	.274	.423	-1.156	-.947	.809	.833
MA06	**I learned about new and useful ways to act.**	**Ich habe nützliche neue Handlungsmöglichkeiten kennengelernt.**	2.24 (1.67)	1.77 (1.49)	.187	.393	-1.192	-.923	.800	.799
MA07	I felt more able to cope with stress.	Ich habe mich besser in der Lage gefühlt, mit Stress umzugehen.	2.68 (1.67)	1.96 (1.57)	-.161	.248	-1.161	-1.033	.803	—

Note. Descriptive statistics are reported for the complete English sample (n=1,153) and the German sample (n=714). Confirmatory factor analyses (CFAs) for item selection were calculated in the selection stratum (n=577) of the English Sample. The five items that were selected for the final GCMQ are written in bold font.

Table S6. *Candidate Items for the Relationship (RE) Scale, Descriptive Statistics, and Factor Loadings for Confirmatory Factor Analyses (CFAs) Calculated for Item Selection.*

	Item text		Mean (SD)		Skewness		Kurtosis		Factor loadings	
Item	English	German	English	German	English	German	English	German	Before selection	After selection
RE01	**I felt safe with the person(s) I was with.**	**Ich habe mich sicher gefühlt mit der Person/den Personen, die bei mir war(en).**	4.01 (1.19)	3.76 (1.05)	-1.263	-.821	1.202	.692	.815	.841
RE02	**I could trust the person(s) I was with.**	**Ich konnte der Person/den Personen, die bei mir war(en), vertrauen.**	4.06 (1.18)	3.88 (0.99)	-1.310	-.658	1.259	-.081	.913	.939
RE03	**I felt comfortable with the person(s) I was with.**	**Ich habe mich mit der Person/den Personen, die bei mir war(en), wohl gefühlt.**	3.94 (1.31)	3.78 (1.07)	-1.148	-.841	.425	.749	.883	.883
RE04	I felt appreciated by the person(s) I was with.	Ich habe mich von der Person/den Personen, die bei mir war(en), wertgeschätzt gefühlt.	3.20 (1.50)	3.42 (1.21)	-.481	-.665	-.780	.283	.638	—
RE05	**I felt connected to the person(s) I was with.**	**Ich habe mich mit der Person/den Personen, die bei mir war(en), verbunden gefühlt.**	3.35 (1.46)	3.29 (1.33)	-.585	-.620	-.599	-.146	.690	.634
RE06	**I felt supported by the person(s) I was with.**	**Ich habe mich von der Person/den Personen, die bei mir war(en), unterstützt gefühlt.**	3.60 (1.41)	3.39 (1.19)	-.774	-.566	-.374	-.060	.832	.801
RE07	There was an emotional bond between myself and the person(s) I was with.	Es gab eine emotionale Bindung zwischen mir und der Person/den Personen, die bei mir war(en).	3.22 (1.61)	3.20 (1.38)	-.512	-.559	-.890	-.373	.614	—

Note. Items of the Relationship (RE) scale were only administered to the (n=699) participants who indicated that a supporting person was present during their reported experience. Descriptive statistics are reported for the complete English sample (n=430) and the German sample (n=269). Confirmatory factor analyses (CFAs) for item selection were calculated in the selection stratum (n=216) of the English Sample. The five items that were selected for the final GCMQ are written in bold font.

Table S7. *Fit Indices for All Confirmatory Factor Analysis (CFA) Models Calculated in the Item Selection Process (Reported in Tables S2-S6).*

	Model fit before item selection			Model fit after item selection		
Model/Scale	RMSEA	CFI	SRMR	RMSEA	CFI	SRMR
Resource Activation (RA)	.078	.940	.036	.074	.985	.020
Problem Actuation (PA)	.051	.981	.029	.023	.998	.010
Clarification (CL)	.080	.954	.030	.048	.994	.013
Mastery (MA)	.091	.963	.026	.090	.978	.020
Relationship (RE)	.191	.801	.076	.113	.953	.034

Note. RMSEA = Root Mean Square Error of Approximation; CFI = Comparative Fit Index; SRMR = Standardized Root Mean Residual; Eng = English sample; Ger = German sample. Note that the RMSEA is a poor indicator of fit in models with small degrees of freedom when applying conventional cutoff values (Kenny et al., 2015).

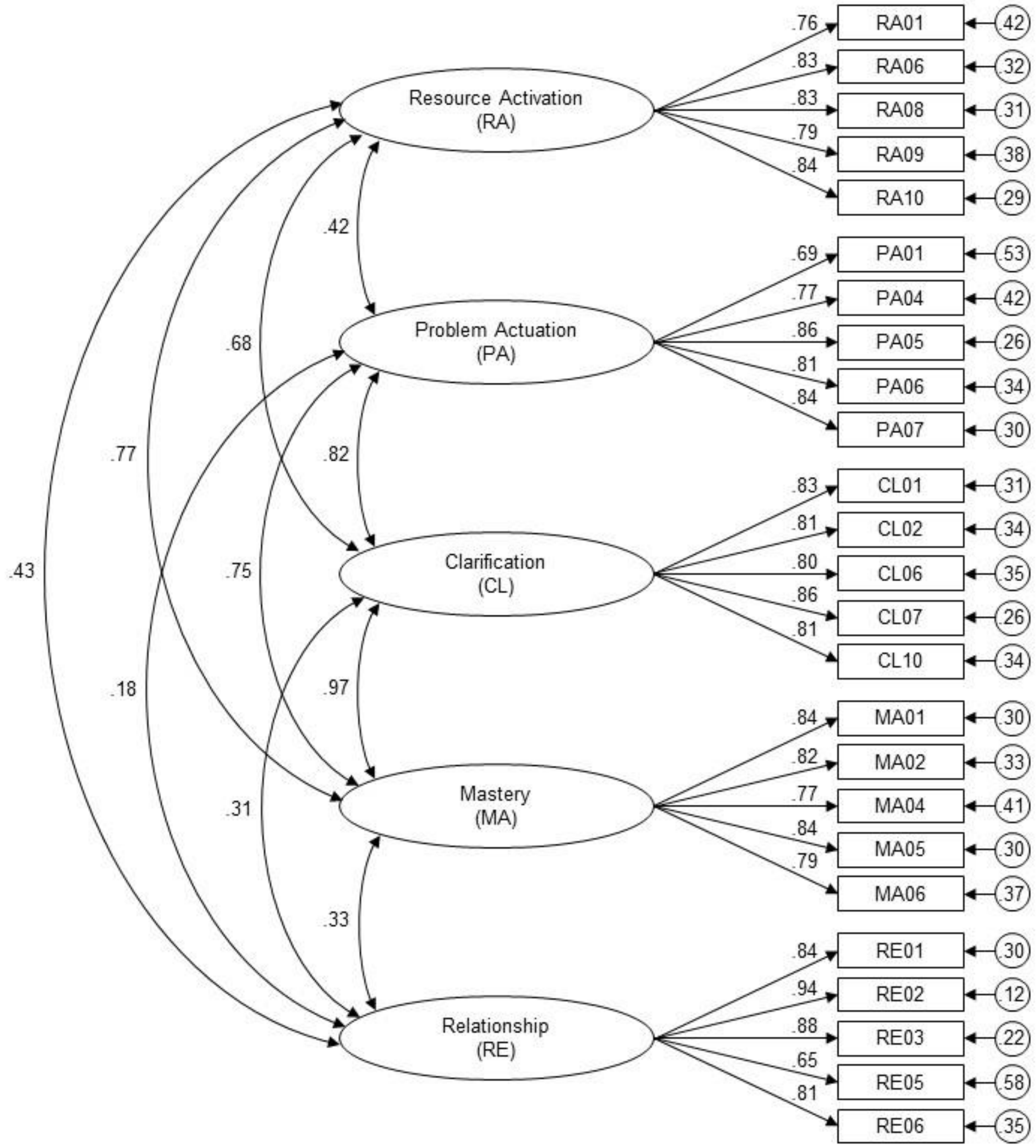

Figure S1. Summary of the selected measurement model in the selection stratum of the English sample (n = 577). All coefficients are standardized. This baseline model showed acceptable fit (RMSEA = 0.063; CFI = 0.923; SRMR = 0.078) and significantly better fit compared to a more constrained alternative model where the two factors CL and MA were collapsed into one single factor ($\chi 2^{diff}$ = 40.840; p < .001).

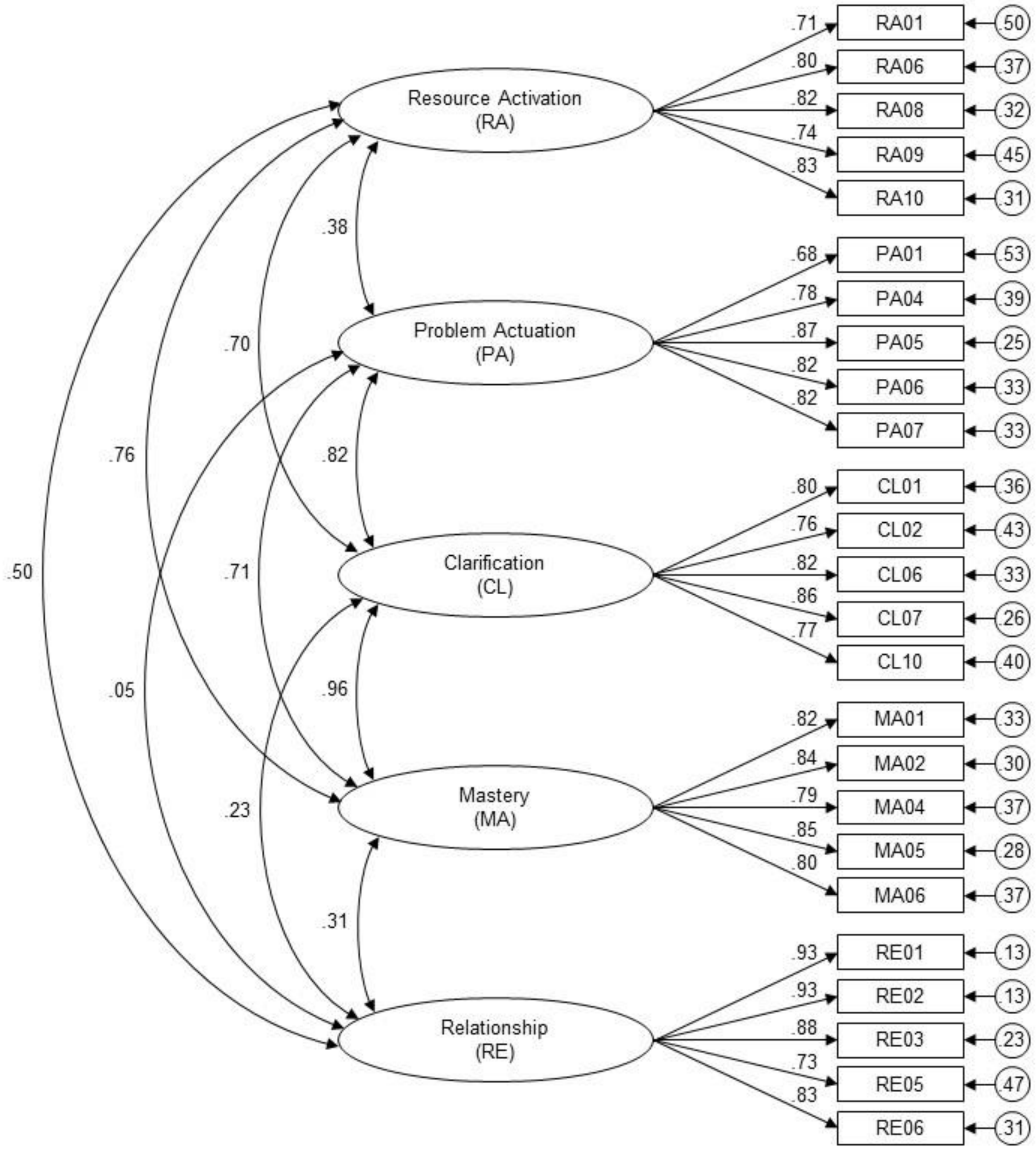

Figure S2. Summary of the selected measurement model in the replication stratum of the English sample (n = 576). All coefficients are standardized. This baseline model showed acceptable fit (RMSEA = 0.064; CFI = 0.920; SRMR = 0.068) and significantly better fit compared to a more constrained alternative model where the two factors CL and MA were collapsed into one single factor ($\chi 2^{diff}$ = 48.658; p <.001.

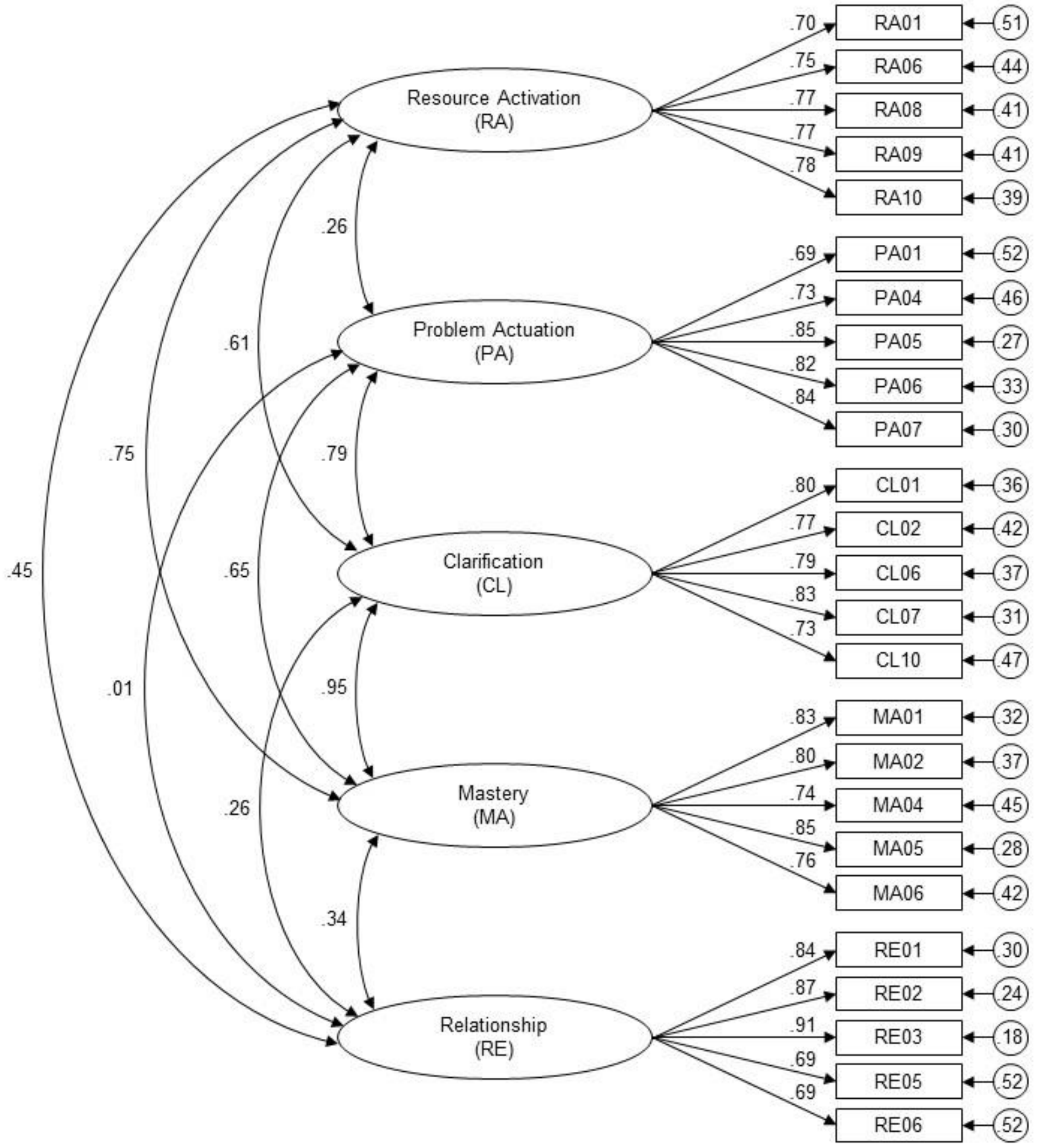

Figure S3. Summary of the selected measurement model in the complete German sample (n = 714). All coefficients are standardized. This baseline model showed acceptable fit (RMSEA = 0.067; CFI = 0.907; SRMR = 0.068) and significantly better fit compared to a more constrained alternative model where the two factors CL and MA were collapsed into one single factor ($\chi 2^{diff}$ = 79.757; p <.001).

Table S8. *Characteristics of Included Participants and Comparisons Between the English and German Sample.*

	Total (cross-language) sample (*N*=1,867)	English sample (*N*=1,153)	German sample (*N*=714)	*t* or χ^2	*p*	Effect size (Cohen's *d* or Cramer's *V*/φ)
Mean (*SD*) age	33.5 (9.7)	34.2 (10.0)	32.2 (9.3)	4.179	<.001	.207
Gender				15.784	<.001	.092
Male	64.3%	64,8%	63.6%			
Female	32.5%	30.9%	35.2%			
Other	3.2%	4.3%	1.3%			
CASMIN classification of education level				136.051	<.001	.270
Tertiary education (highest)	77.0%	85.6%	63.2%			
Secondary education	20.7%	12.1%	34.6%			
Primary education (lowest)	2.2%	2.3%	2.2%			
Lifetime diagnosis of mental disorder	56.1%	64.0%	43.3%	76.930	<.001	.203
Depression	39.3%	45.1%	29.8%	43.103	<.001	.152
Anxiety disorder	25.7%	34.9%	10.9%	132.326	<.001	.266
ADHD	15.7%	20.7%	7.7%	56.387	<.001	.174
PTSD	12.9%	16.7%	6.6%	40.602	<.001	.147
Addiction	8.9%	10.3%	6.7%	7.009	.008	.061
Mania	3.0%	4.2%	1.0%	16.199	<.001	.093
Psychosis	1.9%	2.0%	1.7%	.237	.627	.011
Other	11.1%	10.9%	11.3%	.078	.781	-.006
Mean (SD) cumulative stressful life events	9.0 (8.8)	10.7 (9.7)	6.3 (6.3)	10.620	<.001	.538
Mean (SD) well-being (WEMWBS)	50.0 (8.8)	49.0 (9.2)	51.7 (8.0)	-6.330	<.001	-.313

Note. CASMIN = Comparative Analysis of Social Mobility in Industrial Nations (Brauns et al., 2003); ADHD = Attention deficit hyperactivity disorder; PTSD = Post-traumatic stress disorder; WEMWBS = Warwick-Edinburgh Mental Wellbeing Scale.

Table S9. *Characteristics of Reported Psychedelic Experiences and Comparisons Between the English and German Sample.*

	Total (cross-language) sample (N=1,867)	English sample (n=1,153)	German sample (n=714)	t or χ^2	p	Effect size (Cohen's d or Cramer's V/φ)
M (*SD*) months elapsed since experience	17.2 (17.2)	16.0 (17.1)	19.2 (17.2)	-3.893	<.001	-.187
Subjective clarity of memory[a]				11.276	.010	.078
Completely clear	25.2%	26.9%	22.4%			
Very clear	43.3%	43.0%	43.8%			
Clear	23.2%	21.1%	26.6%			
Somewhat clear	8.3%	9.0%	7.1%			
Psychedelic used				105.376	<.001	.238
LSD	45.7%	37.0%	59.8%			
Psilocybin or psilocybin-containing mushrooms	44.2%	53.2%	29.8%			
Ayahuasca	8.6%	8.2%	9.2%			
Mescaline or mescaline-containing cacti	1.4%	1.6%	1.1%			
Subjective dose strength				18.547	<.001	.100
Low	4.2%	5.0%	2.8%			
Moderate	36.7%	35.0%	39.5%			
High	38.4%	36.8%	40.9%			
Very high	15.8%	17.4%	13.2%			
Extremely high	4.9%	5.7%	3.6%			
Valence of acute effects				22.711	<.001	.110
Rather pleasant	54.5%	53.9%	55.5%			
Rather unpleasant	5.1%	3.5%	7.8%			
Both pleasant and unpleasant	38.5%	40.3%	35.6%			
Neither pleasant nor unpleasant	1.9%	2.3%	1.1%			
Retrospective appraisal				14.548	.006	.088
Very positive	64.2%	66.1%	61.2%			
Positive	27.5%	25.8%	30.3%			
Neutral	5.2%	5.6%	4.5%			
Negative	2.4%	1.6%	3.6%			
Very negative	0.7%	0.9%	0.4%			

Table S9 (continued).

	Total (cross-language) sample (*N*=1,867)	English sample (*n*=1,153)	German sample (*n*=714)	*t* or χ^2	*p*	Effect size (Cramer's *V*/*φ*)
Concomitant substance use						
None	59.6%	58.4%	61.5%	1.777	.183	-.031
Cannabis	29.9%	32.4%	25.9%	8.727	.003	.068
Alcohol	10.9%	8.9%	14.1%	12.309	<.001	-.081
Entactogens	3.7%	2.8%	5.2%	7.176	.007	-.062
Dissociatives	3.0%	2.4%	3.9%	3.379	.066	-.043
Stimulants	2.4%	1.8%	3.4%	4.446	.035	-.049
Benzodiazepines	0.7%	1.0%	0.3%	2.896	.089	.089
Opiates/opioids	0.7%	0.7%	0.7%	.000	.987	.000
Other psychoactive substance(s)	2.2%	2.5%	1.7%	1.430	.232	.028
Use motives (component scores)[b]						
M (*SD*) therapeutic intention	0.00 (1.00)	0.16 (1.00)	-0.26 (0.94)	9.114	<.001	.434
M (*SD*) hedonic intention	0.00 (1.00)	-0.10 (0.98)	0.16 (1.01)	-5.431	<.001	-.259
M (*SD*) escapist intention	0.00 (1.00)	0.12 (1.04)	-0.20 (0.90)	6.895	<.001	.360
Setting categories						
Nature or close-to-nature setting	57.6%	53.3%	64.6%	23.106	<.001	-.111
Setting designed for therapeutic purpose	13.4%	16.0%	9.2%	17.142	<.001	.096
Ceremonial, religious, or spiritual setting	11.4%	10.1%	13.4%	4.745	.029	-.050
Party, concert, or festival	10.4%	8.0%	14.4%	19.591	<.001	-.102
Retrospective suitability of setting				2.799	.562	.040
Very well suited	44.7%	45.5%	45.0%			
Well suited	34.1%	36.0%	34.8%			
Somewhat suited	14.8%	13.4%	14.3%			
Hardly suited	4.7%	3.4%	4.2%			
Not suited at all	1.7%	1.7%	1.7%			
Presence of other people				56.227	<.001	.174
0 (alone)	29.4%	34.9%	20.4%			
1-5 people	55.2%	52.8%	59.0%			
6-15 people	8.9%	7.6%	11.1%			
16-30 people	2.9%	2.2%	4.2%			
31-100 people	1.2%	1.0%	1.7%			
>100 people	2.4%	1.6%	3.6%			
Presence of supporting person(s)	37.4%	37.3%	37.5%	.011	.917	-.002

Table S9 (continued).

	Total (cross-language) sample (N=1,867)	English sample (n=1,153)	German sample (n=714)	t or χ^2	p	Effect size (Cramer's V/φ)
Psychedelic use prior to reported experience				43.628	<.001	.153
0 (never used before)	16.3%	16.3%	16.2%			
1-5 times	26.1%	22.7%	31.7%			
6-20 times	23.8%	22.1%	26.5%			
21-50 times	15.3%	16.8%	12.9%			
51-100 times	8.8%	10.0%	6.9%			
>100 times	9.7%	12.1%	5.9%			
Assigned class membership				20.981	<.001	.106
Profile 1: Moderately therapeutic experience	30.00%	30.80%	28.70%			
Profile 2: Problem-focused experience	7.10%	6.20%	8.50%			
Profile 3: Resource-focused experience	27.50%	25.60%	30.50%			
Profile 4: Non-therapeutic experience	16.40%	15.60%	17.60%			
Profile 5: Highly therapeutic experience	19.00%	21.80%	14.60%			

Note. [a] There were ten volunteers who indicated their memory of the reported experience was "not clear at all". These volunteers were excluded, hence frequencies for the response option "not clear at all" are not reported here. [b] Component scores were extracted from the principal component analysis (PCA) reported in Table 3.

Exploratory Factor Analyses (EFAs) of GCMQ Items

Exploratory factor analyses (EFAs) were calculated to further explore the factor structure of the final 25 GCMQ items. EFA results were largely aligned with CFA results. In the total (cross-language) sample, EFA yielded a three-factor structure (Table S10). That Problem Actuation (PA), Clarification (CL), and Mastery (MA) items loaded strongly on a common factor reflects the strong correlations between the factors Problem Actuation, Clarification, and Mastery found with CFA in the theorized five-factor model (see Figure 2). Note that with CFA, the three-factor model where Problem Actuation, Clarification, and Mastery were collapsed into one single factor showed poor model fit and significantly worse fit compared to the five-factor model. Furthermore, a four-factor model were Clarification and Mastery were collapsed into a "Corrective Experience" factor showed acceptable model fit but still significantly worse fit compared to the theorized five-factor model (see Table 5).

An additional EFA in the subsample of experiences that took place in therapeutic settings yielded a four-factor structure suggesting separate factors Corrective Experience (i.e., Clarification and Mastery) and Problem Actuation (Table S11). This suggests that future clinical studies might yield more differentiated factor structures. Likewise, considering that clarification experiences often entail mastery experiences and vice versa (Grawe, 2004), it can be expected that administering the GCMQ immediately after dosing sessions will yield more separable Clarification and Mastery factors.

Table S10. *Item Loadings from Exploratory Factor Analysis (EFA) of GCMQ Items in the Total (Cross-Language) Sample (N=1,867).*

Item	Factor 1	Factor 2	Factor 3
PA05	**.844**	-.040	.268
PA07	**.798**	-.133	.350
CL06	**.796**	-.185	.516
PA06	**.779**	-.079	.387
CL07	**.767**	-.231	.656
CL01	**.752**	-.216	.591
MA05	**.735**	-.245	.677
PA04	**.729**	-.048	.285
MA01	**.708**	-.338	.675
CL02	**.700**	-.210	.595
PA01	**.670**	-.077	.216
RE02	.096	**-.920**	.317
RE03	.046	**-.886**	.302
RE01	.052	**-.874**	.304
RE06	.228	**-.781**	.393
RE05	.215	**-.682**	.537
RA10	.259	-.355	**.784**
RA09	.346	-.346	**.774**
RA08	.337	-.377	**.772**
RA06	.365	-.342	**.743**
MA06	.588	-.234	**.738**
RA01	.287	-.383	**.717**
MA02	.648	-.243	**.711**
MA04	.607	-.323	**.706**
CL10	.649	-.319	**.703**

Note. The highest loading of each item is written in bold font. EFA was conducted using maximum likelihood factor extraction and oblimin rotation. The Scree plot and Kaiser's criterion suggested that a three-factor solution was appropriate. The three factors cumulatively explained 67.7% of the variance.

Table S11. *Item Loadings from Exploratory Factor Analysis (EFA) of GCMQ Items in the Cross-Language Subsample of Experiences that Occurred in Settings Designed for Therapeutic Purposes (n=250).*

Item	Factor 1	Factor 2	Factor 3	Factor 4
CL07	**.822**	-.167	-.521	.539
MA01	**.817**	-.308	-.415	.446
CL01	**.803**	-.316	-.498	.325
MA05	**.778**	-.251	-.469	.600
CL10	**.775**	-.217	-.521	.552
CL06	**.754**	-.191	-.593	.368
MA02	**.728**	-.323	-.365	.508
CL02	**.720**	-.146	-.525	.346
MA04	**.609**	-.310	-.433	.523
RE02	.278	**-.951**	-.037	.279
RE03	.237	**-.843**	.011	.311
RE01	.239	**-.828**	.061	.175
RE06	.297	**-.792**	-.166	.387
RE05	.392	**-.728**	-.181	.628
PA05	.583	-.084	**-.861**	.234
PA07	.529	-.186	**-.813**	.301
PA06	.538	-.079	**-.785**	.281
PA04	.374	.003	**-.688**	.233
PA01	.434	-.007	**-.615**	.017
RA10	.480	-.321	-.147	**.814**
RA08	.451	-.305	-.241	**.783**
RA06	.428	-.260	-.252	**.731**
RA09	.486	-.361	-.225	**.726**
MA06	.616	-.243	-.386	**.725**
RA01	.522	-.415	-.043	**.558**

Note. The highest loading of each item is written in bold font. EFA was conducted using maximum likelihood factor extraction and oblimin rotation. The Scree plot and Kaiser's criterion suggested that a four-factor solution was appropriate. The four factors cumulatively explained 68.7% of the variance.

Table S12. Estimated Factor Means for the Latent Profiles Identified with Factor Mixture Modeling.

Factor	Estimated standardized factor mean relative to Profile 5 (*p*)				
	Profile 1: Moderately therapeutic experience	Profile 2: Problem-focused experience	Profile 3: Resource-focused experience	Profile 4: Non-therapeutic experience	Profile 5: Highly therapeutic experience
Resource Activation (RA)	-0.91 (<.001)	-3.74 (<.001)	-0.66 (<.001)	-3.63 (<.001)	0.00 (—)
Problem Actuation (PA)	-3.66 (<.001)	-0.42 (.101)	-7.14 (<.001)	-7.82 (<.001)	0.00 (—)
Clarification (CL)	-1.00 (<.001)	-1.19 (<.001)	-1.97 (<.001)	-2.97 (<.001)	0.00 (—)
Mastery (MA)	-1.00 (<.001)	-2.16 (<.001)	-1.85 (<.001)	-2.97 (<.001)	0.00 (—)
Relationship (RE)	-0.49 (<.001)	-1.30 (<.001)	-0.05 (.619)	-0.85 (.010)	0.00 (—)

Table S13. *Psychometric Characterization of Latent Profiles Based on Assigned Class Membership.*

Scale	Total (cross-language) sample (*N*=1,867)	Profile 1: Moderately therapeutic experience (*n*=560)	Profile 2: Problem-focused experience (*n*=133)	Profile 3: Resource-focused experience (*n*=513)	Profile 4: Non-therapeutic experience (*n*=306)	Profile 5: Highly therapeutic experience (*n*=355)	*F*	*p*	*η2*
	M (*SD*)								
GCMQ									
Resource Activation (RA)	3.05 (1.33)	3.36 (0.90)	1.22 (0.82)	3.56 (0.78)	1.20 (0.71)	4.09 (0,73)	806.36	<.001	.634
Problem Actuation (PA)	2.08 (1.47)	2.38 (0,54)	3.79 (0,86)	0.83 (0,55)	0.52 (0,55)	4.09 (0,55)	2,592.90	<.001	.848
Clarification (CL)	2.59 (1.40)	3.00 (0.96)	2.81 (1.30)	2.07 (1.12)	1.00 (0.99)	3.98 (0.86)	404.49	<.001	.465
Mastery (MA)	2.33 (1.39)	2.77 (1.00)	1.61 (1.15)	1.98 (1.14)	0.77 (0.82)	3.75 (0.92)	414.99	<.001	.471
Relationship (RE)	3.73 (1.07)	3.60 (1.00)	2.78 (1.37)	4.09 (0.80)	3.04 (1.10)	4.17 (0.85)	37.64	<.001	.187
APEQ									
Acceptance	55.3 (24.5)	63.6 (16.2)	45.5 (24.4)	50.2 (20.1)	26.4 (19.0)	78.1 (14.6)	381.79	<.001	.451
Avoidance	22.0 (21.8)	21.9 (19.0)	53.2 (24.1)	11.2 (12.9)	16.6 (19.9)	30.7 (22.5)	158.14	<.001	.254
Introspection	70.3 (24.8)	76.4 (18.7)	79.1 (18.8)	64.8 (23.7)	46.2 (27.5)	86.0 (14.5)	174.58	<.001	.273
Interaction	59.4 (25.4)	58.7 (25.2)	47.2 (25.6)	67.4 (22.8)	56.0 (24.7)	56.7 (26.7)	23.92	<.001	.049
EBI	49.0 (30.9)	59.3 (22.1)	49.9 (27.6)	34.8 (25.4)	17.2 (20.8)	80.3 (18.6)	399.37	<.001	.462
CEQ	1.04 (0.97)	1.07 (0.84)	2.52 (1.05)	0.50 (0.46)	0.74 (0.81)	1.49 (1.02)	207.26	<.001	.308
11-ASC OBN	58.9 (25.1)	63.9 (21.8)	37.6 (23.5)	64.4 (20.5)	34.0 (21.7)	72.5 (19.5)	192.94	<.001	.293

Note. GCMQ = General Change Mechanisms Questionnaire; APEQ = Acceptance/Avoidance-Promoting Experiences Questionnaire; EBI = Emotional Breakthrough Inventory; CEQ = Challenging Experience Questionnaire; 11-ASC OBN = 11 Oceanic Boundlessness Scale of the Altered States of Consciousness Questionnaire.

Table S14. *Odds Ratios Estimated by Multinomial Logistic Regression of Assigned Class Membership (Compared to the Reference Class Profile 2 – Problem-Focused Experience) on Context Factors.*

	Profile 1: Moderately therapeutic experience		Profile 3: Resource-focused experience		Profile 4: Non-therapeutic experience		Profile 5: Highly therapeutic experience	
Predictor	OR (95% CI)	*p*	OR (95% CI)	*p*	OR (95% CI)	*p*	OR (95% CI)	*p*
Use motives								
Therapeutic intention	2.16 (1.73; 2.69)	<.001	1.38 (1.11; 1.72)	.004	0.65 (0.51; 0.81)	<.001	2.76 (2.17; 3.52)	<.001
Hedonic intention	1.30 (1.04; 1.62)	.021	1.99 (1.59; 2.50)	<.001	1.42 (1.12; 1.81)	0.004	1.10 (0.87; 1.39)	0.444
Escapist intention	0.64 (0.54; 0.77)	<.001	0.48 (0.40; 0.58)	<.001	0.71 (0.59; 0.85)	<.001	0.73 (0.60; 0.87)	<.001
Setting categories								
Nature or close-to-nature setting	1.03 (0.68; 1.55)	.907	1.26 (0.83; 1.93)	.284	0.79 (0.50; 1.22)	.283	1.07 (0.69; 1.67)	.749
Setting designed for therapeutic purpose	0.56 (0.30; 1.05)	.071	0.44 (0.22; 0.88)	.019	0.41 (0.18; 0.94)	.036	1.18 (0.63; 2.23)	.604
Ceremonial, religious, or spiritual setting	1.10 (0.52; 2.30)	.808	1.15 (0.54; 2.48)	.716	0.77 (0.31; 1.90)	.565	1.39 (0.66; 2.93)	.390
Party, concert, or festival	1.05 (0.56; 1.97)	.887	0.64 (0.34; 1.21)	.174	0.45 (0.23; 0.89)	.021	0.59 (0.27; 1.28)	.183

Note. OR = odds ratio; CI = confidence interval.

Table S15. *Odds Ratios Estimated by Multinomial Logistic Regression of Assigned Class Membership (Compared to the Reference Class Profile 3 – Resource-Focused Experience) on Context Factors.*

	Profile 1: Moderately therapeutic experience		Profile 2: Problem-focused experience		Profile 4: Non-therapeutic experience		Profile 5: Highly therapeutic experience	
Predictor	OR (95% CI)	*p*	OR (95% CI)	*p*	OR (95% CI)	*p*	OR (95% CI)	*p*
Use motives								
Therapeutic intention	1.57 (1.36; 1.81)	<.001	0.73 (0.58; 0.91)	.004	0.47 (0.40; 0.56)	<.001	2.01 (1.68; 2.41)	<.001
Hedonic intention	0.65 (0.57; 0.75)	<.001	0.50 (0.40; 0.63)	<.001	0.71 (0.60; 0.85)	<.001	0.55 (0.47; 0.65)	<.001
Escapist intention	1.33 (1.15; 1.54)	<.001	2.07 (1.71; 2.50)	<.001	1.47 (1.25; 1.74)	<.001	1.50 (1.27; 1.76)	<.001
Setting categories								
Nature or close-to-nature setting	0.81 (0.62; 1.07)	.133	0.79 (0.52; 1.21)	.284	0.62 (0.45; 0.86)	.004	0.85 (0.62; 1.17)	.319
Setting designed for therapeutic purpose	1.26 (0.80; 2.00)	.324	2.27 (1.14; 4.49)	.019	0.94 (0.46; 1.93)	.858	2.68 (1.68; 4.26)	<.001
Ceremonial, religious, or spiritual setting	0.95 (0.61; 1.49)	.827	0.87 (0.40; 1.87)	.716	0.66 (0.33; 1.35)	.256	1.20 (0.75; 1.94)	.446
Party, concert, or festival	1.63 (1.08; 2.45)	.020	1.55 (0.82; 2.92)	.174	0.70 (0.43; 1.16)	.164	0.92 (0.50; 1.69)	.779

Note. OR = odds ratio; CI = confidence interval.

Table S16. *Odds Ratios Estimated by Multinomial Logistic Regression of Assigned Class Membership (Compared to the Reference Class Profile 4 – Non-Therapeutic Experience) on Context Factors.*

	Profile 1: Moderately therapeutic experience		Profile 2: Problem-focused experience		Profile 3: Resource-focused experience		Profile 5: Highly therapeutic experience	
Predictor	OR (95% CI)	*p*	OR (95% CI)	*p*	OR (95% CI)	*p*	OR (95% CI)	*p*
Use motives								
Therapeutic intention	3.34 (2.81; 3.98)	<.001	1.55 (1.23; 1.95)	<.001	2.13 (1.80; 2.52)	<.001	4.28 (3.48; 5.27)	<.001
Hedonic intention	0.91 (0.77; 1.09)	.301	0.70 (0.55; 0.90)	.004	1.40 (1.18; 1.67)	<.001	0.77 (0.64; 0.94)	0.009
Escapist intention	0.90 (0.78; 1.06)	.201	1.41 (1.17; 1.69)	<.001	0.68 (0.58; 0,80)	<.001	1.02 (0.86; 1.21)	0.828
Setting categories								
Nature or close-to-nature setting	1.31 (0.95; 1.80)	.103	1.27 (0.82; 1.98)	.283	1.61 (1.17; 2.22)	.004	1.37 (0.95; 1.97)	.088
Setting designed for therapeutic purpose	1.35 (0.68; 2.66)	.392	2.42 (1.06; 5.52)	.036	1.07 (0.52; 2.19)	.858	2.86 (1.45; 5.66)	.003
Ceremonial, religious, or spiritual setting	1.43 (0.72; 2.87)	.310	1.31 (0.53; 3.25)	.565	1.51 (0.74; 3.06)	.256	1.81 (0.89; 3.69)	.101
Party, concert, or festival	2.32 (1.39; 3.87)	.001	2.21 (1.13; 4.34)	.021	1.43 (0.87; 2.35)	.164	1.31 (0.65; 2.60)	.450

Note. OR = odds ratio; CI = confidence interval.

Table S17. *Odds Ratios Estimated by Multinomial Logistic Regression of Assigned Class Membership (Compared to the Reference Class Profile 5 – Highly Therapeutic Experience) on Context Factors.*

	Profile 1: Moderately therapeutic experience		Profile 2: Problem-focused experience		Profile 3: Resource-focused experience		Profile 4: Non-therapeutic experience	
Predictor	OR (95% CI)	*p*	OR (95% CI)	*p*	OR (95% CI)	*p*	OR (95% CI)	*p*
Use motives								
Therapeutic intention	0.78 (0.66; 0.93)	.005	0.36 (0.28; 0.46)	<.001	0.50 (0.42; 0.60)	<.001	0.23 (0.19; 0.29)	<.001
Hedonic intention	1.18 (1.02; 1.38)	.032	0.91 (0.72; 1.15)	.444	1.82 (1.54; 2.14)	<.001	1.30 (1.07; 1.57)	.009
Escapist intention	0.89 (0.77; 1.02)	.097	1.38 (1.15; 1.66)	<.001	0.67 (0.57; 0.79)	<.001	0.98 (0.83; 1.17)	.828
Setting categories								
Nature or close-to-nature setting	0.95 (0.71; 1.28)	.751	0.93 (0.60; 1.45)	.749	1.17 (0.86; 1.61)	.319	0.73 (0.51; 1.05)	.088
Setting designed for therapeutic purpose	0.47 (0.32; 0.69)	<.001	0.85 (0.45; 1.59)	.604	0.37 (0.24; 0.59)	<.001	0.35 (0.18; 0.69)	.003
Ceremonial, religious, or spiritual setting	0.79 (0.52; 1.21)	.277	0.72 (0.34; 1.52)	.390	0.83 (0.52; 1.34)	.446	0.55 (0.27; 1.12)	.101
Party, concert, or festival	1.78 (0.97; 3.26)	.064	1.70 (0.78; 3.69)	.183	1.09 (0.59; 2.02)	.779	0.77 (0.38; 1.53)	.450

Note. OR = odds ratio; CI = confidence interval.

References

Grawe, K. (2004). *Psychological therapy*. Hogrefe Publishing.

Kenny, D. A., Kaniskan, B., & McCoach, D. B. (2015). The Performance of RMSEA in Models With Small Degrees of Freedom. *Sociological Methods & Research*, *44*(3), 486–507. https://doi.org/10.1177/0049124114543236
