# Supplementary material for: Measuring psychotherapeutic processes in the context of psychedelic experiences: Validation of the General Change Mechanisms Questionnaire (GCMQ)
Source: J Psychopharmacol. 2024 May 14;38(5):432–57. doi: 10.1177/02698811241249698 (PMC11102652; doi:10.1177/02698811241249698)
Supplement: sj-pdf-2-jop-10.1177_02698811241249698 – Supplemental material for Measuring psychotherapeutic processes in the context of psychedelic experiences: Validation of the General Change Mechanisms Questionnaire (GCMQ) [file sj-pdf-2-jop-10.1177_02698811241249698.pdf]

General Change Mechanisms Questionnaire (GCMQ)

Paper-and-Pencil Versions and Scoring Instructions

This document includes three versions of the GCMQ in English and German:

1. The **GCMQ-20** includes four scales Resource Activation (RA), Problem Actuation (PA), Clarification (CL), and Mastery (MA). The scale Relationship (RE) is not included in the GCMQ-20. The GCMQ-20 is therefore applicable to settings where no therapist or supporting person was present (e.g., recreational psychedelic use).
2. The **GCMQ-25-NC** (NC = nonclinical) includes all five scales (RA, PA, CL, MA, and RE). Items in the RE scale do not refer to "my therapist(s)", but instead refer to "the person(s) I was with". The GCMQ-25-NC is therefore applicable outside of formal clinical settings.
3. The **GCMQ-25-CL** (CL = clinical) includes all five scales (RA, PA, CL, MA, and RE). Items in the RE scale refer to "my therapist(s)". The GCMQ-25-CL is therefore applicable to formal clinical settings.

Each item is scored on a 6-point Likert scale ranging from 0 ("not at all") to 5 ("extremely"). Scores for the scales are calculated as the sum of all items of the respective scale. Item positions for the three GCMQ versions are listed in the following:

	**Resource Activation (RA)**	**Problem Actuation (PA)**	**Clarification (CL)**	**Mastery (MA)**	**Relationship (RE)**
**GCMQ-20**	3, 6, 9, 14, 17	1, 8, 11, 15, 20	2, 5, 12, 16, 19	4, 7, 10, 13, 18	—
**GCMQ-25-NC** and **GCMQ-25-CL**	4, 7, 11, 18, 21	1, 9, 14, 19, 25	2, 6, 15, 20, 24	5, 8, 12, 16, 23	3, 10, 13, 17, 22

(cc) BY This work is licensed under a Creative Commons Attribution 4.0 International License

GCMQ-20

Here is a list of statements that may apply to your experience. Please estimate the extent to which these statements apply to your experience.

		not at all	a little	moderately	strongly	very strongly	extremely
1	I felt emotions that are related to my problems.	○	○	○	○	○	○
2	It became clearer to me what the essence of my problems is.	○	○	○	○	○	○
3	I could see myself in a positive light.	○	○	○	○	○	○
4	I found new ways to deal with situations that have caused me problems in the past.	○	○	○	○	○	○
5	I could see my problems in a new light.	○	○	○	○	○	○
6	I became aware of the beautiful things in my life.	○	○	○	○	○	○
7	I gained confidence in my ability to handle strong feelings.	○	○	○	○	○	○
8	I was confronted with my problems.	○	○	○	○	○	○
9	I felt in contact with my strengths.	○	○	○	○	○	○
10	I made progress in coping with my problems.	○	○	○	○	○	○
11	I experienced feelings that I previously avoided.	○	○	○	○	○	○
12	It became clearer to me why I tend to react in certain ways to certain difficult situations.	○	○	○	○	○	○

Please also complete the next page ⟶

This work is licensed under a Creative Commons Attribution 4.0 International License

		not at all	a little	moderately	strongly	very strongly	extremely
13	I learned about new and useful ways to act.	○	○	○	○	○	○
14	I felt connected to things that give my life meaning.	○	○	○	○	○	○
15	I relived painful or fearful memories.	○	○	○	○	○	○
16	I gained a new understanding of the difficulties in my life.	○	○	○	○	○	○
17	I became aware of things that I am thankful for.	○	○	○	○	○	○
18	I learned things that can help me handle difficult situations.	○	○	○	○	○	○
19	I could understand my feelings on a deeper level.	○	○	○	○	○	○
20	I could feel that my vulnerable spots were being touched upon.	○	○	○	○	○	○

This work is licensed under a Creative Commons Attribution 4.0 International License

GCMQ-20

Hier ist eine Liste von Aussagen, die möglicherweise auf Ihre Erfahrung zutreffen. Bitte schätzen Sie den Umfang ein, in dem diese Aussagen auf Ihre Erfahrung zutreffen.

		überhaupt nicht	ein wenig	mäßig	stark	sehr stark	extrem
1	Ich spürte Emotionen, die mit meinen Problemen zusammenhängen.	○	○	○	○	○	○
2	Es wurde mir klarer, was der Kern meiner Probleme ist.	○	○	○	○	○	○
3	Ich konnte mich selbst in einem positiven Licht sehen.	○	○	○	○	○	○
4	Ich habe neue Wege gefunden, mit Situationen umzugehen, die mir in der Vergangenheit Probleme gemacht haben.	○	○	○	○	○	○
5	Ich konnte meine Probleme in einem neuen Licht sehen.	○	○	○	○	○	○
6	Ich bin mir der schönen Dinge in meinem Leben bewusst geworden.	○	○	○	○	○	○
7	Ich habe Vertrauen in meine Fähigkeit gewonnen, mit starken Gefühlen umzugehen.	○	○	○	○	○	○
8	Ich wurde mit meinen Problemen konfrontiert.	○	○	○	○	○	○
9	Ich fühlte mich im Kontakt mit meinen Stärken.	○	○	○	○	○	○
10	Ich habe Fortschritte bei der Bewältigung meiner Probleme gemacht.	○	○	○	○	○	○
11	Ich habe Gefühle erlebt, die ich zuvor vermieden habe.	○	○	○	○	○	○
12	Mir ist klarer geworden, warum ich in bestimmten schwierigen Situationen dazu neige, auf bestimmte Weise zu reagieren.	○	○	○	○	○	○

Bitte füllen Sie auch die nächste Seite aus ⟶

This work is licensed under a Creative Commons Attribution 4.0 International License

		überhaupt nicht	ein wenig	mäßig	stark	sehr stark	extrem
13	Ich habe nützliche neue Handlungsmöglichkeiten kennengelernt.	○	○	○	○	○	○
14	Ich habe mich Dingen verbunden gefühlt, die meinem Leben Sinn geben.	○	○	○	○	○	○
15	Ich habe schmerzhafte oder angstvolle Erinnerungen wiedererlebt.	○	○	○	○	○	○
16	Ich habe ein neues Verständnis für die Schwierigkeiten in meinem Leben gewonnen.	○	○	○	○	○	○
17	Mir wurden Dinge bewusst, für die ich dankbar bin.	○	○	○	○	○	○
18	Ich habe Dinge gelernt, die mir dabei helfen können, mit schwierigen Situationen umzugehen.	○	○	○	○	○	○
19	Ich konnte meine Gefühle auf einer tieferen Ebene verstehen.	○	○	○	○	○	○
20	Ich konnte spüren, dass meine verletzlichen Stellen berührt wurden.	○	○	○	○	○	○

This work is licensed under a Creative Commons Attribution 4.0 International License

GCMQ-25-NC

Here is a list of statements that may apply to your experience. Please estimate the extent to which these statements apply to your experience.

		not at all	a little	moderately	strongly	very strongly	extremely
1	I felt emotions that are related to my problems.	○	○	○	○	○	○
2	It became clearer to me what the essence of my problems is.	○	○	○	○	○	○
3	I could trust the person(s) I was with.	○	○	○	○	○	○
4	I could see myself in a positive light.	○	○	○	○	○	○
5	I found new ways to deal with situations that have caused me problems in the past.	○	○	○	○	○	○
6	I could see my problems in a new light.	○	○	○	○	○	○
7	I became aware of the beautiful things in my life.	○	○	○	○	○	○
8	I gained confidence in my ability to handle strong feelings.	○	○	○	○	○	○
9	I was confronted with my problems.	○	○	○	○	○	○
10	I felt comfortable with the person(s) I was with.	○	○	○	○	○	○
11	I felt in contact with my strengths.	○	○	○	○	○	○
12	I made progress in coping with my problems.	○	○	○	○	○	○

Please also complete the next page ⟶

This work is licensed under a Creative Commons Attribution 4.0 International License

		not at all	a little	moderately	strongly	very strongly	extremely
13	I felt supported by the person(s) I was with.	○	○	○	○	○	○
14	I experienced feelings that I previously avoided.	○	○	○	○	○	○
15	It became clearer to me why I tend to react in certain ways to certain difficult situations.	○	○	○	○	○	○
16	I learned about new and useful ways to act.	○	○	○	○	○	○
17	I felt safe with the person(s) I was with.	○	○	○	○	○	○
18	I felt connected to things that give my life meaning.	○	○	○	○	○	○
19	I relived painful or fearful memories.	○	○	○	○	○	○
20	I gained a new understanding of the difficulties in my life.	○	○	○	○	○	○
21	I became aware of things that I am thankful for.	○	○	○	○	○	○
22	I felt connected to the person(s) I was with.	○	○	○	○	○	○
23	I learned things that can help me handle difficult situations.	○	○	○	○	○	○
24	I could understand my feelings on a deeper level.	○	○	○	○	○	○
25	I could feel that my vulnerable spots were being touched upon.	○	○	○	○	○	○

This work is licensed under a Creative Commons Attribution 4.0 International License

GCMQ-25-NC

Hier ist eine Liste von Aussagen, die möglicherweise auf Ihre Erfahrung zutreffen. Bitte schätzen Sie den Umfang ein, in dem diese Aussagen auf Ihre Erfahrung zutreffen.

		überhaupt nicht	ein wenig	mäßig	stark	sehr stark	extrem
1	Ich spürte Emotionen, die mit meinen Problemen zusammenhängen.	○	○	○	○	○	○
2	Es wurde mir klarer, was der Kern meiner Probleme ist.	○	○	○	○	○	○
3	Ich konnte der Person/den Personen, die bei mir war(en), vertrauen.	○	○	○	○	○	○
4	Ich konnte mich selbst in einem positiven Licht sehen.	○	○	○	○	○	○
5	Ich habe neue Wege gefunden, mit Situationen umzugehen, die mir in der Vergangenheit Probleme gemacht haben.	○	○	○	○	○	○
6	Ich konnte meine Probleme in einem neuen Licht sehen.	○	○	○	○	○	○
7	Ich bin mir der schönen Dinge in meinem Leben bewusst geworden.	○	○	○	○	○	○
8	Ich habe Vertrauen in meine Fähigkeit gewonnen, mit starken Gefühlen umzugehen.	○	○	○	○	○	○
9	Ich wurde mit meinen Problemen konfrontiert.	○	○	○	○	○	○
10	Ich habe mich mit der Person/den Personen, die bei mir war(en), wohl gefühlt.	○	○	○	○	○	○
11	Ich fühlte mich im Kontakt mit meinen Stärken.	○	○	○	○	○	○
12	Ich habe Fortschritte bei der Bewältigung meiner Probleme gemacht.	○	○	○	○	○	○

Bitte füllen Sie auch die nächste Seite aus ⟶

This work is licensed under a Creative Commons Attribution 4.0 International License

		überhaupt nicht	ein wenig	mäßig	stark	sehr stark	extrem
13	Ich habe mich von der Person/den Personen, die bei mir war(en), unterstützt gefühlt.	○	○	○	○	○	○
14	Ich habe Gefühle erlebt, die ich zuvor vermieden habe.	○	○	○	○	○	○
15	Mir ist klarer geworden, warum ich in bestimmten schwierigen Situationen dazu neige, auf bestimmte Weise zu reagieren.	○	○	○	○	○	○
16	Ich habe nützliche neue Handlungsmöglichkeiten kennengelernt.	○	○	○	○	○	○
17	Ich habe mich sicher gefühlt mit der Person/den Personen, die bei mir war(en).	○	○	○	○	○	○
18	Ich habe mich Dingen verbunden gefühlt, die meinem Leben Sinn geben.	○	○	○	○	○	○
19	Ich habe schmerzhafte oder angstvolle Erinnerungen wiedererlebt.	○	○	○	○	○	○
20	Ich habe ein neues Verständnis für die Schwierigkeiten in meinem Leben gewonnen.	○	○	○	○	○	○
21	Mir wurden Dinge bewusst, für die ich dankbar bin.	○	○	○	○	○	○
22	Ich habe mich mit der Person/den Personen, die bei mir war(en), verbunden gefühlt.	○	○	○	○	○	○
23	Ich habe Dinge gelernt, die mir dabei helfen können, mit schwierigen Situationen umzugehen.	○	○	○	○	○	○
24	Ich konnte meine Gefühle auf einer tieferen Ebene verstehen.	○	○	○	○	○	○
25	Ich konnte spüren, dass meine verletzlichen Stellen berührt wurden.	○	○	○	○	○	○

This work is licensed under a Creative Commons Attribution 4.0 International License

GCMQ-25-CL

Here is a list of statements that may apply to your therapy session. Please estimate the extent to which these statements apply to your therapy session.

		not at all	a little	moderately	strongly	very strongly	extremely
1	I felt emotions that are related to my problems.	○	○	○	○	○	○
2	It became clearer to me what the essence of my problems is.	○	○	○	○	○	○
3	I could trust my therapist(s).	○	○	○	○	○	○
4	I could see myself in a positive light.	○	○	○	○	○	○
5	I found new ways to deal with situations that have caused me problems in the past.	○	○	○	○	○	○
6	I could see my problems in a new light.	○	○	○	○	○	○
7	I became aware of the beautiful things in my life.	○	○	○	○	○	○
8	I gained confidence in my ability to handle strong feelings.	○	○	○	○	○	○
9	I was confronted with my problems.	○	○	○	○	○	○
10	I felt comfortable with my therapist(s).	○	○	○	○	○	○
11	I felt in contact with my strengths.	○	○	○	○	○	○
12	I made progress in coping with my problems.	○	○	○	○	○	○

Please also complete the next page ⟶

This work is licensed under a Creative Commons Attribution 4.0 International License

		not at all	a little	moderately	strongly	very strongly	extremely
13	I felt supported by my therapist(s).	○	○	○	○	○	○
14	I experienced feelings that I previously avoided.	○	○	○	○	○	○
15	It became clearer to me why I tend to react in certain ways to certain difficult situations.	○	○	○	○	○	○
16	I learned about new and useful ways to act.	○	○	○	○	○	○
17	I felt safe with my therapist(s).	○	○	○	○	○	○
18	I felt connected to things that give my life meaning.	○	○	○	○	○	○
19	I relived painful or fearful memories.	○	○	○	○	○	○
20	I gained a new understanding of the difficulties in my life.	○	○	○	○	○	○
21	I became aware of things that I am thankful for.	○	○	○	○	○	○
22	I felt connected to my therapist(s).	○	○	○	○	○	○
23	I learned things that can help me handle difficult situations.	○	○	○	○	○	○
24	I could understand my feelings on a deeper level.	○	○	○	○	○	○
25	I could feel that my vulnerable spots were being touched upon.	○	○	○	○	○	○

This work is licensed under a Creative Commons Attribution 4.0 International License

GCMQ-25-CL

Hier ist eine Liste von Aussagen, die möglicherweise auf Ihre Therapiesitzung zutreffen. Bitte schätzen Sie den Umfang ein, in dem diese Aussagen auf Ihre Therapiesitzung zutreffen.

		überhaupt nicht	ein wenig	mäßig	stark	sehr stark	extrem
1	Ich spürte Emotionen, die mit meinen Problemen zusammenhängen.	○	○	○	○	○	○
2	Es wurde mir klarer, was der Kern meiner Probleme ist.	○	○	○	○	○	○
3	Ich konnte meiner Therapeutin/meinem Therapeuten/meinen Therapeut:innen vertrauen.	○	○	○	○	○	○
4	Ich konnte mich selbst in einem positiven Licht sehen.	○	○	○	○	○	○
5	Ich habe neue Wege gefunden, mit Situationen umzugehen, die mir in der Vergangenheit Probleme gemacht haben.	○	○	○	○	○	○
6	Ich konnte meine Probleme in einem neuen Licht sehen.	○	○	○	○	○	○
7	Ich bin mir der schönen Dinge in meinem Leben bewusst geworden.	○	○	○	○	○	○
8	Ich habe Vertrauen in meine Fähigkeit gewonnen, mit starken Gefühlen umzugehen.	○	○	○	○	○	○
9	Ich wurde mit meinen Problemen konfrontiert.	○	○	○	○	○	○
10	Ich habe mich mit meiner Therapeutin/ meinem Therapeuten/meinen Therapeut:innen wohl gefühlt.	○	○	○	○	○	○
11	Ich fühlte mich im Kontakt mit meinen Stärken.	○	○	○	○	○	○
12	Ich habe Fortschritte bei der Bewältigung meiner Probleme gemacht.	○	○	○	○	○	○

Bitte füllen Sie auch die nächste Seite aus ⟶

This work is licensed under a Creative Commons Attribution 4.0 International License

		überhaupt nicht	ein wenig	mäßig	stark	sehr stark	extrem
13	Ich habe mich von meiner Therapeutin/ meinem Therapeuten/meinen Therapeut:innen unterstützt gefühlt.	○	○	○	○	○	○
14	Ich habe Gefühle erlebt, die ich zuvor vermieden habe.	○	○	○	○	○	○
15	Mir ist klarer geworden, warum ich in bestimmten schwierigen Situationen dazu neige, auf bestimmte Weise zu reagieren.	○	○	○	○	○	○
16	Ich habe nützliche neue Handlungsmöglichkeiten kennengelernt.	○	○	○	○	○	○
17	Ich habe mich sicher gefühlt mit meiner Therapeutin/meinem Therapeuten/ meinen Therapeut:innen.	○	○	○	○	○	○
18	Ich habe mich Dingen verbunden gefühlt, die meinem Leben Sinn geben.	○	○	○	○	○	○
19	Ich habe schmerzhafte oder angstvolle Erinnerungen wiedererlebt.	○	○	○	○	○	○
20	Ich habe ein neues Verständnis für die Schwierigkeiten in meinem Leben gewonnen.	○	○	○	○	○	○
21	Mir wurden Dinge bewusst, für die ich dankbar bin.	○	○	○	○	○	○
22	Ich habe mich mit meiner Therapeutin/ meinem Therapeuten/meinen Therapeut:innen verbunden gefühlt.	○	○	○	○	○	○
23	Ich habe Dinge gelernt, die mir dabei helfen können, mit schwierigen Situationen umzugehen.	○	○	○	○	○	○
24	Ich konnte meine Gefühle auf einer tieferen Ebene verstehen.	○	○	○	○	○	○
25	Ich konnte spüren, dass meine verletzlichen Stellen berührt wurden.	○	○	○	○	○	○

This work is licensed under a Creative Commons Attribution 4.0 International License
